# The majority of A-to-I RNA editing is not required for mammalian homeostasis

**DOI:** 10.1186/s13059-019-1873-2

**Published:** 2019-12-09

**Authors:** Alistair M. Chalk, Scott Taylor, Jacki E. Heraud-Farlow, Carl R. Walkley

**Affiliations:** 1 St. Vincent’s Institute of Medical Research, 9 Princes St, Fitzroy, VIC 3065 Australia; 2 Department of Medicine, St. Vincent’s Hospital, Melbourne Medical School, University of Melbourne, Fitzroy, VIC 3065 Australia; 3 Mary MacKillop Institute for Health Research, Australian Catholic University, Melbourne, VIC 3000 Australia

**Keywords:** A-to-I editing, ADAR1, ADAR2, RNA editing, Epitranscriptome, RNA modification

## Abstract

**Background:**

Adenosine-to-inosine (A-to-I) RNA editing, mediated by ADAR1 and ADAR2, occurs at tens of thousands to millions of sites across mammalian transcriptomes. A-to-I editing can change the protein coding potential of a transcript and alter RNA splicing, miRNA biology, RNA secondary structure and formation of other RNA species. In vivo, the editing-dependent protein recoding of GRIA2 is the essential function of ADAR2, while ADAR1 editing prevents innate immune sensing of endogenous RNAs by MDA5 in both human and mouse. However, a significant proportion of A-to-I editing sites can be edited by both ADAR1 and ADAR2, particularly within the brain where both are highly expressed. The physiological function(s) of these shared sites, including those evolutionarily conserved, is largely unknown.

**Results:**

To generate completely A-to-I editing-deficient mammals, we crossed the viable rescued ADAR1-editing-deficient animals (*Adar1*^*E861A/E861A*^*Ifih1*^*−/−*^) with rescued ADAR2-deficient (*Adarb1*^*−/−*^*Gria2*^*R/R*^) animals. Unexpectedly, the global absence of editing was well tolerated. *Adar1*^*E861A/E861A*^*Ifih1*^*−/−*^*Adarb1*^*−/−*^*Gria2*^*R/R*^ were recovered at Mendelian ratios and age normally. Detailed transcriptome analysis demonstrated that editing was absent in the brains of the compound mutants and that ADAR1 and ADAR2 have similar editing site preferences and patterns.

**Conclusions:**

We conclude that ADAR1 and ADAR2 are non-redundant and do not compensate for each other’s essential functions in vivo. Physiologically essential A-to-I editing comprises a small subset of the editome, and the majority of editing is dispensable for mammalian homeostasis. Moreover, in vivo biologically essential protein recoding mediated by A-to-I editing is an exception in mammals.

## Background

The conversion of adenosine to inosine in RNA (A-to-I RNA editing) is a widespread feature of the transcriptome [1], with tens of thousands of A-to-I sites identified in mouse and millions in human [2–4]. Inosine is interpreted as guanosine upon translation or sequencing, meaning A-to-I editing leads to post-transcriptional A-to-G transitions in RNA. Editing occurs within regions of double-stranded RNA (dsRNA), and inosine has different thermodynamic base pairing properties to adenosine, harboring the potential to alter both the RNA code and the secondary structure [5–8]. A-to-I editing levels vary across transcripts, tissues, and throughout development ranging from < 1 to 100% at any given site [4, 9]. Editing can occur at specific adenosines within a transcript, termed site-selective editing, or at many sites within an extended region, termed hyperediting or editing enriched regions [3, 10–12]. The vast majority of editing is weak and occurs within repetitive elements (e.g., Alu elements in humans, SINEs in mice). In mice and humans, A-to-I editing is catalyzed by the adenosine deaminase acting on RNA family members ADAR1 (*Adar*) and ADAR2 (*Adarb1*). The third mammalian ADAR, ADAR3 (*Adarb2*), does not have detectable editing activity [13, 14]. The prevailing view is that site-selective editing is primarily associated with ADAR2, while hyperediting is associated with ADAR1 [2, 4].

Altered expression or mutation of ADARs is associated with several human diseases. Loss of function mutations in *ADAR* causes the infantile encephalopathy Aicardi-Goutières syndrome (AGS) [15]. AGS patients develop a characteristic type I interferonopathy, a transcriptional signature first associated with loss of ADAR1 in the mouse [16, 17]. ADAR1 is overexpressed in a number of cancers which is postulated to contribute to cancer progression and proteome diversity [18, 19]. Recent work identified a number of cancers to be highly sensitive to loss of ADAR1 and depletion of ADAR1 enhanced activity of immunotherapy [20–22]. Reduced ADAR2 activity and overall editing levels have been reported in central nervous system (CNS) diseases, including amyotrophic lateral sclerosis, autism, and brain cancers [23, 24]. While the consequences of mutations in the writers of A-to-I editing are clear, the physiological roles and functions of the majority of editing sites are undetermined.

The most striking outcome of A-to-I editing is protein recoding, where editing directly changes the amino acid sequence of the translated protein from that encoded genomically. Recoding of the ADAR2-specific Q/R site in the glutamate receptor *Gria2* is essential for post-natal viability in mice [25]. *Adarb1*^*−/−*^ *(Adar2*^*−/−*^) animals die several weeks after birth and were rescued by homozygous single residue A-to-G mutation in the genomic DNA at the edited Q/R codon of *Gria2*, mimicking the constitutive recoding at this site [25]. The *Adarb1*^*−/−*^*Gria2*^*R/R*^ rescued animals are remarkably normal indicating that this single editing site accounts for the lethality, with several subtle phenotypes subsequently reported in the viable rescued animals [26, 27]. The contributions of the majority of protein recoding events outside of GRIA2 and the reasons for the evolutionary conservation of a subset of them is largely unknown [28, 29].

Editing can change splice sites, miRNA binding sites, and pre- and mature miRNAs, as well as alter the production of circular RNAs. However, the vast majority of mammalian editing occurs in repetitive elements/retrotransposons such as short interspersed elements (SINEs) and long interspersed elements (LINEs), including primate restricted *Alu* elements, which can form structured long dsRNA [2, 9]. Physiologically, editing by ADAR1 attenuates the immunogenic potential of endogenous dsRNA and prevents an MDA5-mediated innate immune response to self-dsRNA in both human and mouse [30–35]. *Adar*^*−/−*^ (*Adar1*^*−/−*^) or editing-deficient (*Adar1*^*E861A/E861A*^) animals die in utero at E11.75-E12.5 [36, 37] and E13.5 [30], respectively, which can be rescued by loss of the cytosolic dsRNA sensor MDA5 (encoded by *Ifih1*) or its downstream effector MAVS [30–32]. The same genetic pathway is present in human ADAR1-deficient cell lines, with the MDA5/MAVS axis being the principal physiological sensor of unedited endogenous dsRNA [31, 34]. This demonstrates a conserved mammalian response to unedited RNA that is not dependent on primate restricted *Alu* elements. The requirement for editing by Adar1 appears to be distinct to that of ADAR2, and essential ADAR1-dependent protein recoding events, analogous to ADAR2/*Gria2*, have not been identified [38].

Our understanding of the in vivo functions of A-to-I editing in mammals is incomplete. There are detailed maps of the numbers, levels, and tissue distribution of A-to-I editing across multiple species [4]. It is known that ADAR1 and ADAR2 share similar sequence neighbor preferences around the edited adenosine [39] and can compensate for each other on many endogenous substrates or when directed for programmable editing [40–42]. Compensatory editing has the potential to mask physiologically important phenotypes and functions of A-to-I editing in the respective single mutant models. Moreover, with tens of thousands of editing events occurring in vivo during murine development and aging, the contribution of these has not been determined outside of a small number of targets. To address this, we have now generated and assessed compound editing-deficient *Adar1*^*E861A/E861A*^*Adarb1*^*−/−*^ null mice also containing the respective *Ifih1*^*−/−*^ and *Gria2*^*R/R*^ suppressor alleles. We show here that mice completely lacking A-to-I editing were recovered at Mendelian ratio. Although half of editing sites in the brain were shared by ADAR1 and ADAR2, including many that are evolutionarily conserved and within coding regions, mice completely lacking A-to-I editing developed and aged normally. This demonstrates that ADAR1 and ADAR2 do not physiologically compensate by editing additional shared essential sites in vivo.

## Results

### A-to-I editing-deficient animals are recovered at the expected frequency and age normally

**Fig. 1:**
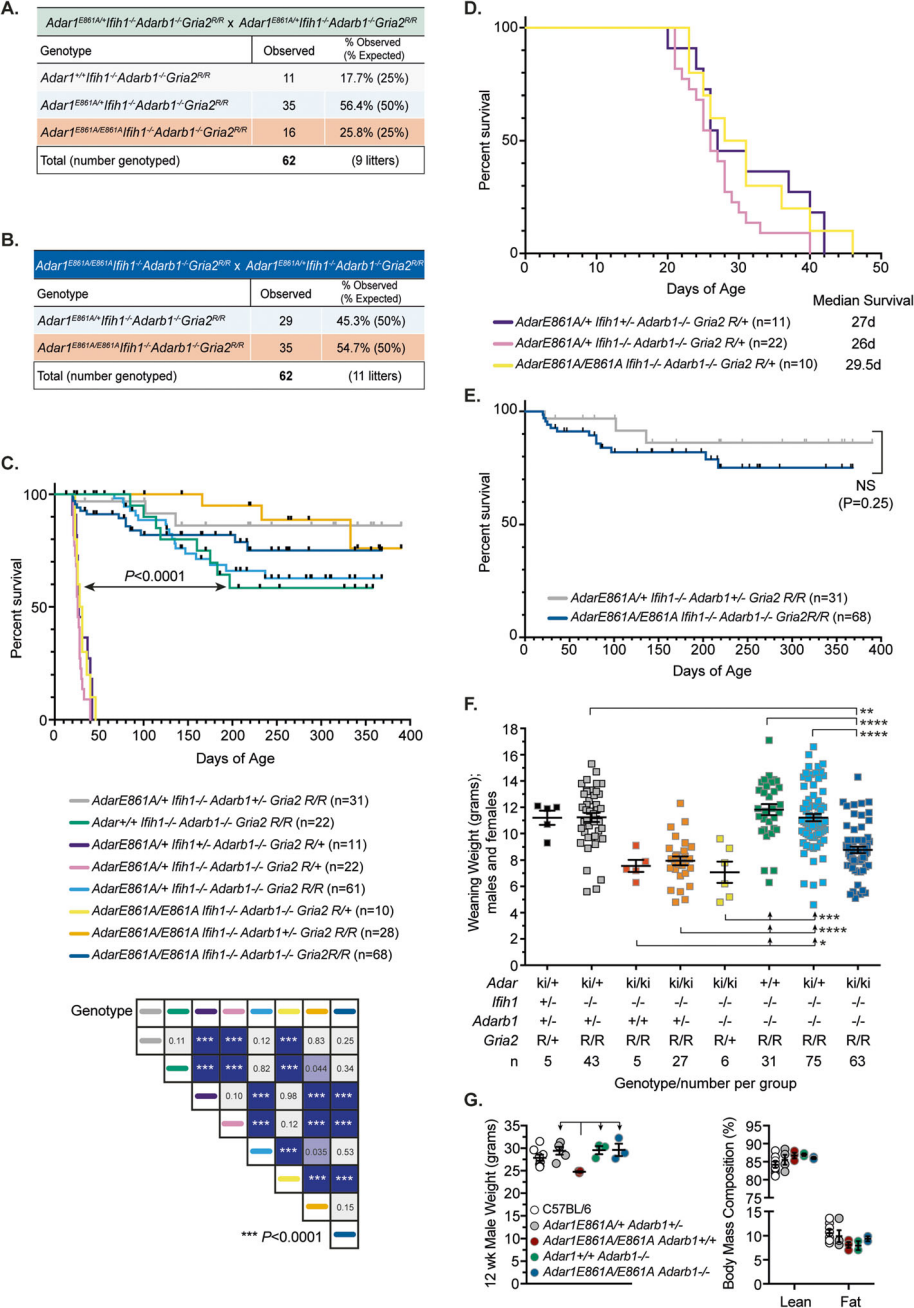
A-to-I editing-deficient mice are viable with a normal lifespan. **a** Breeding data from intercrosses of *Adar1*^*E861A/+*^*Ifih1*^*−/−*^*Adarb1*^*−/−*^*Gria2*^*R/R*^ males and females. **b** Breeding data from intercrosses of *Adar1*^*E861A/E861A*^*Ifih1*^*−/−*^*Adarb1*^*−/−*^*Gria2*^*R/R*^ males with *Adar1*^*E861A/+*^*Ifih1*^*−/−*^*Adarb1*^*−/−*^*Gria2*^*R/R*^ females. **c** Survival data for all genotypes; numbers per genotype and statistical comparison across all genotypes (pairwise log-rank (Mantel-Cox) test); *P* value as indicated or ***P < 0.001. **d** Comparison of survival of *Adarb1*^*−/−*^*Gria2*^*R/+*^ animals with either heterozygous *Adar1*^*E861A/+*^ (purple and pink lines) or homozygous *Adar1*^*E861A/E861A*^ (yellow) and calculated median survival in days. **e** Comparison of survival of *Adar1*^*E861A/+*^*Ifih1*^*−/−*^*Adarb1*^*+/−*^*Gria2*^*R/R*^ (double heterozygous) and *Adar1*^*E861A/E861A*^*Ifih1*^*−/−*^*Adarb1*^*−/−*^*Gria2*^*R/R*^. **f** Weaning weight (~ 20 days of age) of the indicated genotypes; both males and females included. *P < 0.05, **P < 0.01, ***P < 0.001 (ordinary one-way ANOVA with multiple comparisons correction (Tukey’s)). **g** Body weight and body mass composition of 12-week-old males of the indicated genotypes; *n* per genotype: C57Bl/6 = 8 (white circle); dHet (*Adar1*^*E861A/+*^*Ifih1*^*−/−*^*Adarb1*^*+/−*^*Gria2*^*R/R*^) = 5 (gray); *Adar1*^*E861A/E861A*^*Adarb1*^*+/+*^ (*Ifih1*^*−/−*^*Gria2*^*+/+*^) = 3 (red); *Adar1*^*+/+*^*Adarb1*^*−/−*^ (*Ifih1*^*−/−*^*Gria2*^*R/R*^) = 3 (green); *Adar1*^*E861A/E861A*^*Adarb1*^*−/−*^ (*Ifih1*^*−/−*^*Gria2*^*R/R*^) = 3 (blue); *P < 0.05 (ordinary one-way ANOVA with multiple comparisons correction (Tukey’s))

To generate completely A-to-I editing-deficient mammals, we crossed the viable rescued ADAR1-editing-deficient animals (*Adar1*^*E861A/E861A*^*Ifih1*^*−/−*^) [30, 38] with the *Adarb1*^*−/−*^*Gria2*^*R/R*^ animals [25]. From these crosses, we recovered all expected genotypes and assessed their long-term survival (Fig. 1a–e). Surprisingly, completely editing-deficient *Adar1*^*E861A/E861A*^*Ifih1*^*−/−*^*Adarb1*^*−/−*^*Gria2*^*R/R*^ (*Adar1*^*E861A/E861A*^*Adarb1*^*−/−*^) animals were born at the expected Mendelian frequency (Fig. 1a, b) and long-term survival percentages were not significantly different to controls within the available sample size (Fig. 1c–e, Additional file 1: Figure S1). Under standard housing conditions, the *Adar1*^*E861A/E861A*^*Adarb1*^*−/−*^ animals have survived > 1 year of age to date (*n* = 15 > 52 weeks old; *n* = 2 > 80 weeks). Furthermore, the *Adarb1*^*−/−*^*Gria2*^*R/+*^ genotype had reduced post-natal survival consistent with their original description [25] and this was not worsened by the further loss of ADAR1 editing (Fig. 1c, d). This indicates that ADAR1 and ADAR2 are non-redundant during mouse development and lifespan. Strikingly, it also demonstrates that the single adenosine at the Q/R site of *Gria2* represents the only physiologically essential protein recoding event in vivo.

**Fig. 2:**
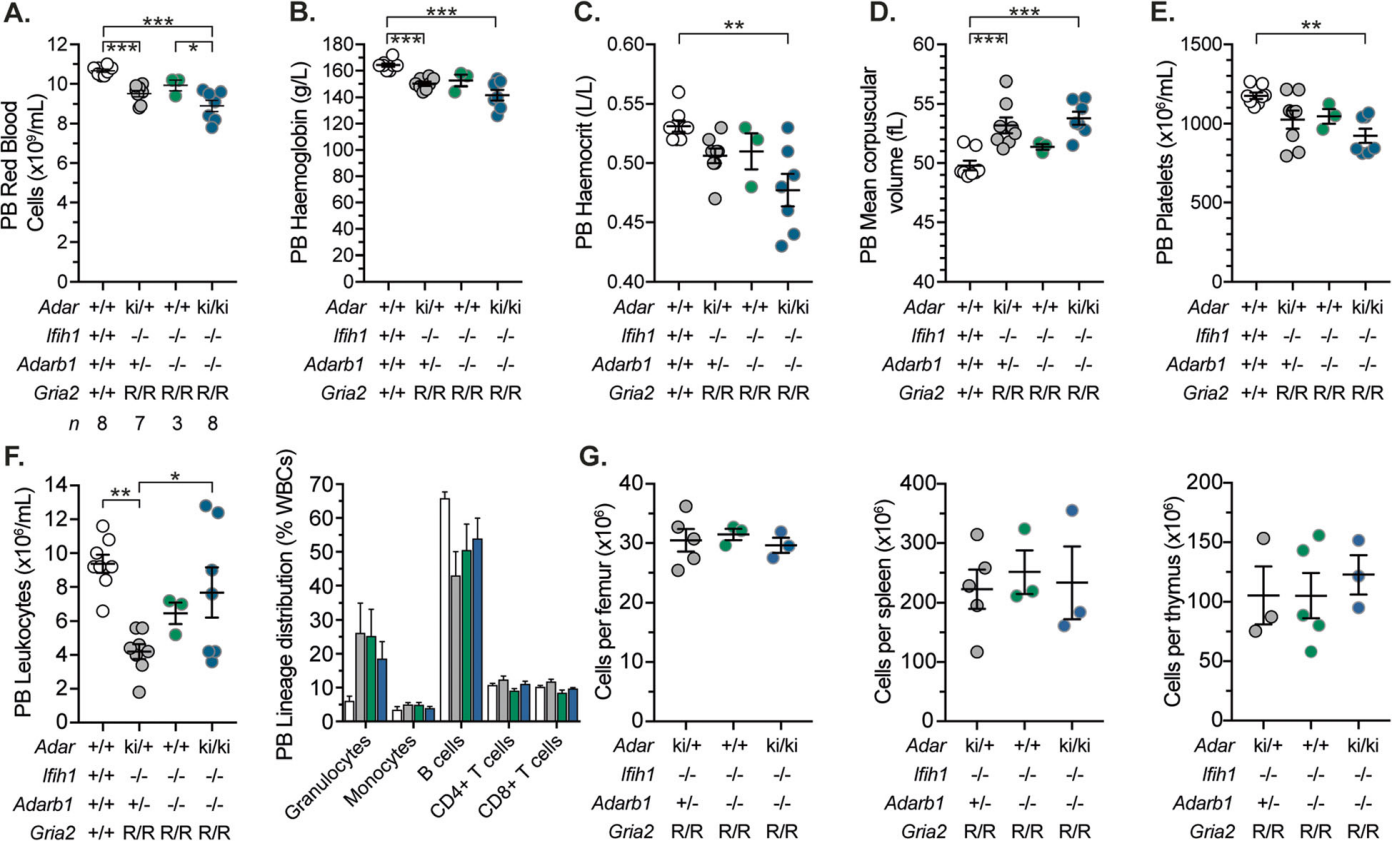
Peripheral blood and hematopoietic parameters of *Adar1*^*E861A/E861A*^*Adarb1*^*−/−*^ mice. **a** Red blood cell counts. **b** Hemoglobin. **c** Hematocrit. **d** Mean corpuscular volume. **e** Platelet count. **f** Peripheral blood leukocyte numbers and lineage distribution from the indicated genotypes, C57Bl/6 = 8 (white circle); dHet (*Adar1*^*E861A/+*^*Ifih1*^*−/−*^*Adarb1*^*+/−*^*Gria2*^*R/R*^) = 8 (gray); *Adar1*^*+/+*^*Adarb1*^*−/−*^ (*Ifih1*^*−/−*^*Gria2*^*R/R*^) = 3 (green); *Adar1*^*E861A/E861A*^*Adarb1*^*−/−*^ (*Ifih1*^*−/−*^*Gria2*^*R/R*^) = 7 (blue), *P < 0.05, **P < 0.01. **g** Cellularity of the femurs, spleen, and thymus from the indicated genotypes, *n* = 3 per genotype. All counts were performed on peripheral blood from 12–18-week-old male animals of the indicated genotypes. Number of animals in each genotype indicated in panel **a**. Statistical analysis: one-way ANOVA with correction for multiple comparisons; *P < 0.05; **P < 0.01; ***P < 0.001

At weaning, *Adar1*^*E861A/E861A*^*Ifih1*^*−/−*^ animals are smaller than controls [38], irrespective of the *Adarb1*/*Gria2* genotype, and this was maintained in *Adar1*^*E861A/E861A*^*Adarb1*^*−/−*^ animals (Fig. 1f). We compared cohorts of adult males from the indicated genotypes to age-/facility-matched C57BL/6 animals. Adult *Adar1*^*E861A/E861A*^*Ifih1*^*−/−*^ animals weighed less than all other genotypes but had a normal body mass composition (Fig. 1g). In contrast, all other genotypes had a normal body weight and body mass composition compared to both C57BL/6 and *Adar1*^*E861A/+*^*Ifih1*^*−/−*^*Adarb1*^*+/−*^*Gria2*^*R/R*^ (double heterozygous; dHet) (Fig. 1g). Therefore, loss of ADAR2 does not modify the initial post-natal runting of *Adar1*^*E861A/E861A*^*Ifih1*^*−/−*^ animals but does enable recovery to normal weight by 12 weeks of age. As hematopoietic cells in the mouse are particularly sensitive to ADAR1 loss, analysis of the hematopoietic populations in the peripheral blood and other hematopoietic organs was performed (Fig. 2). The *Adar1*^*E861A/E861A*^*Adarb1*^*−/−*^ were not significantly different to editing-proficient controls (Fig. 2) [38]. A genotype blinded histological assessment of 20–25-week-old male *Adar1*^*E861A/E861A*^*Adarb1*^*−/−*^ animals and dHets did not find any significant difference between genotypes (Additional file 2: Dataset S1). Therefore, once self-sensing of unedited dsRNA is prevented by loss of MDA5 and the single GRIA2 Q/R site is genomically reinstated, the complete absence of A-to-I editing in vivo is well tolerated. This demonstrates that there are no essential roles of ADAR1 and ADAR2-mediated editing that are compensated for in the single mutants by the other homolog.

### ADAR2 loss does not modify ADAR1-dependent transcriptional signatures.

**Fig. 3:**
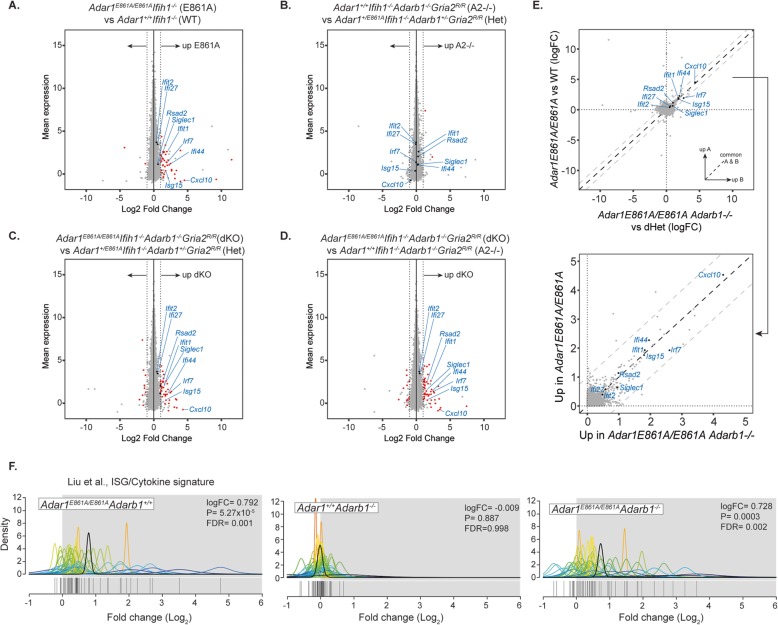
ADAR2 loss does not modify the transcriptional signature associated with the loss of Adar1-mediated RNA editing. Analysis of differential gene expression from 12-week-old male brains of **a** *Adar1*^*+/+*^*Ifih1*^*−/−*^ (WT) compared to *Adar1*^*E861A/E861A*^*Ifih1*^*−/−*^ (E861A); **b** *Adar1*^*+/+*^*Ifih1*^*−/−*^*Adarb1*^*−/−*^*Gria2*^*R/R*^ (ADAR2 null) compared to *Adar1*^*E861A/+*^*Ifih1*^*−/−*^*Adarb1*^*+/−*^*Gria2*^*R/R*^ (dHet); **c** *Adar1*^*E861A/E861A*^*Ifih1*^*−/−*^*Adarb1*^*−/−*^*Gria2*^*R/R*^ (dKO) compared to *Adar1*^*E861A/+*^*Ifih1*^*−/−*^*Adarb1*^*+/−*^*Gria2*^*R/R*^ (dHet); and **d** *Adar1*^*E861A/E861A*^*Ifih1*^*−/−*^*Adarb1*^*−/−*^*Gria2*^*R/R*^ (dKO) compared to *Adar1*^*+/+*^*Ifih1*^*−/−*^*Adarb1*^*−/−*^*Gria2*^*R/R*^ (*Adar2*^*−/−*^); *n* = 3 per genotype; red indicated *FDR* < 0.05. **e** Comparison of the differential gene expression signatures of the E861A (**a**) and dKO (**c**) samples. The increased expression of the transcripts highlighted in blue is shared between murine and human *ADAR1* mutants. Top panel: *y*-axis has the gene expression comparison of the *Adar1*^*E861A/E861A*^ vs WT; *x*-axis has the gene expression comparison of the *Adar1*^*E861A/E861A*^*Adarb1*^*−/−*^ (dKO) vs dHet. Lower panel: *Adar1*^*E861A/E861A*^ compared to *Adar1*^*E861A/E861A*^*Adar2*^*−/−*^ (dKO) with expanded view of the upper right quadrant. **f** QuSAGE pathway analysis of the consensus interferon-stimulated gene (ISG)/cytokine signature defined by Liu et al. [20] for the *Adar1*^*E861A/E861A*^ compared to *Adar1*^*+/+*^*Ifih1*^*−/−*^ (left panel), *Adarb1*^*−/−*^ compared to dHet (center panel), and *Adar1*^*E861A/E861A*^*Adarb1*^*−/−*^ compared to dHet (dKO; right panel); log_2_FC, *P* value and FDR as indicated on each panel

RNA-seq was performed on the whole brain from 12-week-old males to assess changes in gene expression. The brain was chosen as it expresses robust levels of both ADAR1 and ADAR2, unlike many peripheral tissues [38]. For comparison, we included a prior dataset from 12-week-old male *Adar1*^*+/+*^*Ifih1*^*−/−*^ and *Adar1*^*E861A/E861A*^*Ifih1*^*−/−*^ whole brain (Fig. 3a) [38]. Differential gene expression analysis demonstrated that the most significant changes occurred in the *Adar1*^*E861A/E861A*^*Ifih1*^*−/−*^ and *Adar1*^*E861A/E861A*^*Adarb1*^*−/−*^ genotypes where a modest activation of the innate immune/interferon signature was present, including the genes used diagnostically in humans with *ADAR1* mutation (Fig. 3a–e, Additional file 1: Figure S1, Additional file 3: Dataset S2) (15, 20, 38). The loss of ADAR2 editing alone did not significantly impact gene expression (Fig. 3d, e). Notably, the loss of both ADAR1 and ADAR2 editing did not significantly modify gene expression outside of that dependent on the loss of ADAR1 editing (Fig. 3e). Therefore, A-to-I editing is not required for homeostatic gene expression or regulation of gene expression and its absence does not significantly alter the brain transcriptome. Pathway analysis demonstrated that the modest activation of the ISG/cytokine signature is unique to ADAR1-editing-deficient samples, even in the absence of MDA5, and that the loss of ADAR2 does not further exacerbate it (Fig. 3f) [20].

### Transcriptome-wide loss of A-to-I editing is tolerated

**Fig. 4:**
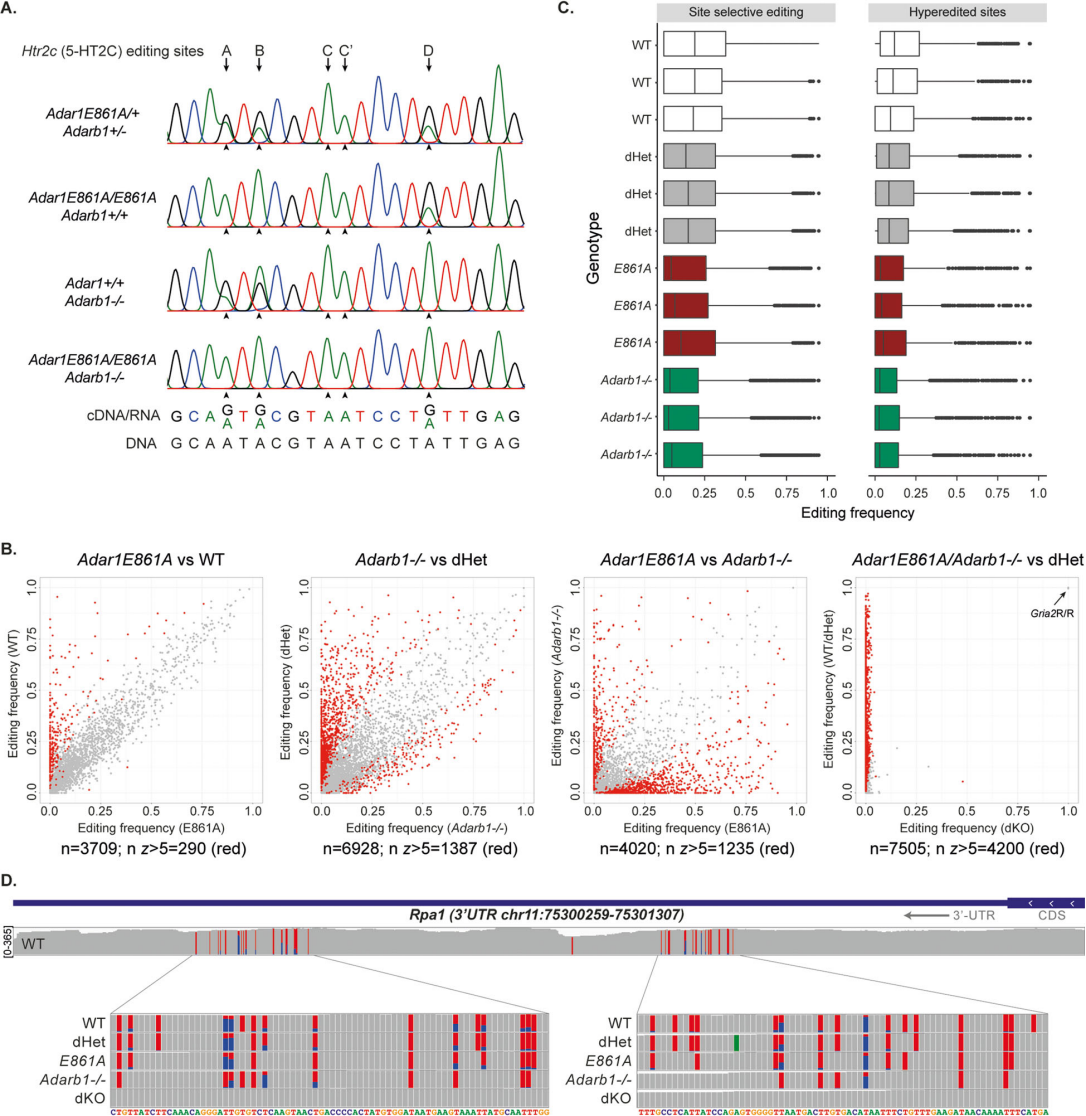
*Adar1*^*E861A/E861A*^*Adarb1*^*−/−*^ have lost A-to-I editing across the transcriptome. **a** Analysis of A-to-I editing of the *Htr2c* receptor at the known sites A–D by Sanger sequencing. Genotypes as indicated. **b** Analysis of editing sites across the genotypes. A dataset of 57,077 murine editing sites was compiled and the datasets assessed for editing at these sites. Sites required ≥ 50 read coverage and an editing rate of ≥ 0.01 (≥ 1%) to be included. The number that passed this threshold for each comparison is listed, and the numbers that are significantly different based on the *z* factor (*z* ≥ 5; Jacusa analysis method) are indicated in red. **c** Editing frequency across coding/site-selective and repetitive/hyperediting sites in the transcriptome in the individual samples from the WT, dHet, *Adar1*^*E861A/E861A*^, and *Adarb1*^*−/−*^. Sites required ≥ 50 read coverage and an editing rate of ≥ 0.01 (≥ 1%) to be considered. Boxplot represents the 25% quantile to 75% quantile with the median indicated. **d** Editing of the 3′UTR of *Rpa1* transcript in each of the indicated genotypes

**Fig. 5:**
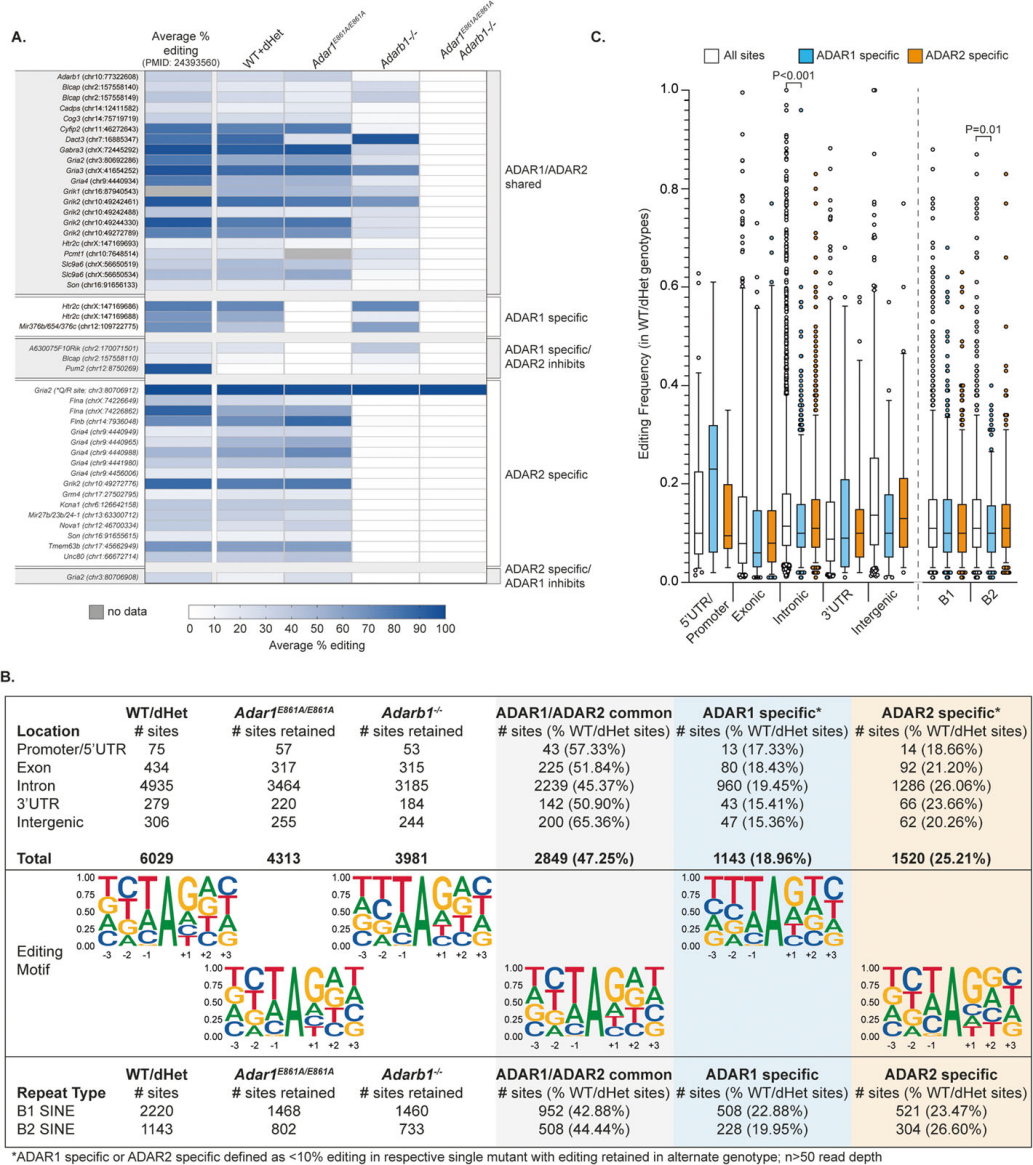
ADAR1- and ADAR2-specific sites are comparable. **a** Analysis of evolutionarily conserved A-to-I editing events across the genotypes. Average editing for each site was calculated plotted with reference to the levels at each site identified by Pinto et al. [29]. ADAR1/ADAR2 shared sites are defined as having > 10% and < 150% editing compared to the average editing rate of the WT and *Adar1*^*E861A/+*^*Adarb1*^*+/−*^ (dHet) samples combined (WT+dHet); ADAR1-specific sites have < 10% editing of this site in the *Adar1*^*E861A/E861A*^ samples and unchanged editing in the *Adarb1*^*−/−*^ compared to WT+dHet; ADAR1-specific/ADAR2 inhibits sites have < 10% editing of this site in the *Adar1*^*E861A/E861A*^ samples and > 150% editing of WT+dHet levels in the *Adarb1*^*−/−*^; ADAR2-specific sites have < 10% editing of this site in the *Adarb1*^*−/−*^ samples and unchanged editing in the *Adar1*^*E861A/E861A*^ compared to WT+dHet; ADAR2-specific/ADAR1 inhibits sites have < 10% editing of this site in the *Adarb1*^*−/−*^ samples and > 150% editing of WT+dHet levels in the *Adar1*^*E861A/E861A*^. **b** Quantitation of the numbers of sites (≥ 50 read coverage and an editing rate of ≥ 0.01 (≥ 1%)) and genomic location across genotypes. ADAR1- or ADAR2-specific sites were defined as having < 10% editing of a site in the one genotype and retained editing in the alternative genotype. The percentage of sites that are ADAR1 or ADAR2 specific is indicated in brackets as a percent of the total number of sites for each location. The sequence context of the editing sites for each classification was derived with Seqlogo. The distribution of editing in B1 and B2 SINEs was mapped from the total sites identified in each genotype. **c** The genomic distribution/repeat type and average editing level for the ADAR1 and ADAR2 sites compared to the all sites observed in the control (WT+dHet genotype combined). Box and whiskers plot with 5–95 percentile shown. No significant difference between genotypes or *P* value as indicated (ANOVA with multiple comparisons correction)

Sanger sequencing of a well-characterized substrate, *Htr2c*, from the 12-week-old male brains confirmed the complete loss of editing at sites A and B in *Adar1*^*E861A/E861A*^ samples, the specific loss of site D from *Adar2*^*−/−*^, and the absence of editing at all sites from the *Adar1*^*E861A/E861A*^*Adarb1*^*−/−*^ (Fig. 4a) [37]. To understand the ADAR1- and ADAR2-dependent editome, we compared the editing sites that were present, absent, or gained in each genotype against a database of 57,077 murine editing sites compiled from published databases (RADAR [8]), previous publications [4, 30, 43], and unpublished murine datasets (JH-F, AMC, and CRW). Analyses of known editing sites demonstrated that a significant proportion could be edited by either ADAR1 or ADAR2 (Fig. 4b), with subsets being ADAR1- or ADAR2-specific (sites clustering on the *y*-axis in the first or second panel, respectively). As anticipated, A-to-I editing at known sites was absent in the *Adar1*^*E861A/E861A*^*Adarb1*^*−/−*^ samples (Fig. 4b; Additional file 1: Figure S3). The loss of A-to-I editing in the double mutant confirmed that there are no alternative enzymes capable of this modification in mouse. Surprisingly, the loss of either ADAR1 or ADAR2 individually had a largely comparable effect on the transcriptome-wide distribution and levels of editing at either site-selective or repetitive/hyperedited regions (hyperediting defined as 10 or more editing sites per 100 bp; Fig. 4c). As an example, *Rpa1* was edited by both ADAR1 and ADAR2 (Fig. 4d). Evolutionarily conserved editing events, except the genomically engineered *Gria2*^*R/R*^, were absent in the *Adar1*^*E861A/E861A*^*Adarb1*^*−/−*^ samples indicating that the global absence of protein recoding does not have a pathogenic effect in vivo, under standard housing conditions (Fig. 5a).

### ADAR1 and ADAR2 do not have unique substrate preferences

Using datasets from all genotypes, we investigated the characteristics of ADAR1- and ADAR2-specific editing events. While from a single tissue and developmental timepoint these datasets are genetically controlled, providing high confidence with which to delineate the characteristics of these sites. When evolutionarily conserved sites were assessed, the majority were either able to be edited equivalently by either ADAR1 or ADAR2 or were ADAR2-specific events. A small number of the conserved sites were ADAR1-specific, and another subset showed a pattern of editing suggesting inhibition of specific editing by the alternative ADAR (Fig. 5a). Such a phenomenon has previously been reported at selected targets in mouse [44] and when assessed transcriptome-wide in *Caenorhabditis elegans* [45]. We then defined the genotype-specific editing events from the entire dataset and assessed the characteristics of these sites. ADAR1-specific and ADAR2-specific sites shared a similar overall number and location/distribution across the transcript (Fig. 5b). Editing of B1 and B2 repeat elements was largely comparable between ADAR1 and ADAR2. When the editing frequency was assessed, ADAR1-specific editing events within B2 elements were edited to a lower average level that the average editing frequency of all sites in B2 elements; however, there was no significant difference between ADAR1 and ADAR2 (Fig. 5c). Despite the single mutant phenotypes, there was no clear preference toward editing of repetitive elements by ADAR1 nor toward site-selective/recoding for ADAR2 (Fig. 5b). Although ADAR2 was responsible for a larger proportion of the conserved editing sites (Fig. 5a), analysis of the sequence context of the edited adenosine did not reveal a strong sequence motif in neighboring nucleotides or difference between ADAR enzymes, consistent with a lack of sequence specificity by dsRNA binding proteins in general (Fig. 5b). The direct comparison of the ADAR1- and ADAR2-specific events did not demonstrate significant differences in editing level by location (Fig. 5c). A recently reported independent analysis of editing sites in a range of ADAR mutant mice also concluded that both ADAR1 and ADAR2 have similar activity and efficiency for both repetitive regions and site-selective editing in vivo [46]. Using definitive genetic controls, we conclude that ADAR1 and ADAR2 have similar editing site distributions and that the sequence context of the edited adenosine does not account for the editing specificity. Cell type-specific expression patterns and cellular localization of ADAR1 and ADAR2, RNA structure, splicing efficiency, *cis*-regulatory elements, or other RNA binding factors may be additional determinants of ADAR specificity [4, 28, 46, 47].

## Discussion

A-to-I editing in the mouse occurs at tens of thousands of sites in both coding and non-coding regions and is developmentally dynamic. Advances in transcriptome sequencing have greatly increased our understanding of the breadth and extent of A-to-I editing across evolution and within a given species. It is now apparent that A-to-I editing is a highly prevalent epitranscriptomic mark in mammals and that it has the potential to influence many important processes including miRNA sequence and biogenesis, miRNA binding sites, RNA splicing, circular RNA biogenesis, and allowing tolerance to repetitive elements and dsRNA structure. While evidence exists for roles of A-to-I editing in all of these processes, the physiological function of the majority of editing sites is unknown.

Mutants of the individual ADAR’s have been reported in the mouse and have provided important insight into the physiological roles of these enzymes. These studies demonstrated that ADAR2 was essential for protein recoding editing [25], particularly in the central nervous system, while ADAR1 was required to prevent sensing of endogenous RNA by MDA5 [3]. These genetic studies have focused attention on a small subset of the tens of thousands of sites potentially edited in the mouse transcriptome during development and aging. The extent of functional redundancy between ADAR1 and ADAR2 to mask important physiological roles of A-to-I editing was unknown, potentially preventing appreciation of meaningful consequences of editing. The complete absence of editing causes phenotypic consequences in both *C. elegans* (chemotaxis) [48, 49] and *Drosophila* (staggering phenotype [50]); however, both species lack an ADAR1 homolog which means the functions of the many sites edited by both ADAR1 and ADAR2 in mammals are not able to be understood [51, 52]. Through interbreeding of the rescued ADAR1-editing-deficient mice and the *Adarb1*^*−/−*^*Gria2*^*R/R*^ animals, we were able to evaluate the organismal requirement for A-to-I editing in mammalian development and homeostasis.

Historically, editing has been most prominently linked to protein recoding, where editing of adenosines within exonic regions changes the genomically encoded amino acid resulting in a protein with altered sequence and, potentially, function. This type of A-to-I editing, particularly in the nervous system, has demonstrated physiologically functional consequences across species [25, 50, 53, 54]. The paradigm for recoding editing remains GRIA2 [25, 55]. More recently, analysis of additional conserved recoding sites including FLNA, NEIL1, and AZIN1 has been reported. The deletion of the conserved editing complementary sequence (ECS) from Filamin A (FLNA) leads to a loss in editing and prevents a Q2341R amino acid change in the mouse. This site is highly edited in both human and mouse cardiovascular tissues and arteries [56]. FLNA^ΔECS^ mice had no apparent abnormalities and normal life expectancy and fertility. However, both isolated aortae and vascular smooth muscle cells had altered function in vitro, and the mice had a mild diastolic hypertension at rest and altered arterial and cardiac remodeling [56]. The editing of NEIL1, a protein implicated in the DNA damage response, leads to a lysine to arginine (K242R) substitution. The two protein forms (unedited K242 and edited R242) have different binding kinetics and affinities for DNA substrates [57]. An increased editing of AZIN1 was identified in hepatocellular carcinoma [58]. The editing of AZIN1 resulted in an S367G recoding event, leading to altered cellular localization of the edited protein and proposed gain-of-function activity. Such studies provide evidence that protein recoding can have specific in vivo functions, demonstrating that these can be important but not essential for viability. It is worth considering that with the exception of the recent in vivo model of FLNA, the conclusions from these studies are derived from the assessment of either 0% or 100% editing in cell lines or as recombinant protein, as this is what can be robustly genetically engineered. The editing levels of these transcripts in vivo yield a mixed population of edited/unedited protein, leading to a more nuanced dynamic between populations of the protein. In vivo analysis of the roles of editing at these evolutionarily conserved sites, such as was undertaken for FLNA, is required to confirm their physiological importance. Rather than focus on the consequences of individual editing events, we have now generated animals completely lacking editing. Strikingly the absence of editing at all sites, including evolutionarily conserved recoding sites, was tolerated in the *Adar1*^*E861A/E861A*^*Adarb1*^*−/−*^ animals. It is important to emphasize that the analysis conducted to date has only assessed a limited number of parameters of the *Adar1*^*E861A/E861A*^*Adarb1*^*−/−*^ animals and these have all been under standard housing conditions. It remains to be determined if functions for editing sites may only be subtle, apparent in vivo under specific conditions or when the *Adar1*^*E861A/E861A*^*Adarb1*^*−/−*^ mice are challenged. While acknowledging that additional phenotypic differences may become apparent with further testing, the current results demonstrate that mammalian development and long-term survival can occur effectively in the absence of A-to-I editing.

By removing editing completely, we can conclude that other proposed consequences of A-to-I editing, such as miRNA recoding/retargeting [59, 60] and involvement in circular RNA biology [61], are not physiologically essential. Furthermore, the datasets generated herein provide a genetically controlled reference set from a single tissue and developmental timepoint for testing computational methods and will provide a resource for further understanding of A-to-I editing in vivo. The direct extrapolation of our findings to humans requires deliberation, particularly as the detailed mapping of editing across species has demonstrated that humans/primates have a significantly greater number of editing sites than rodents [4, 7, 28]. The majority of editing in humans occurs in the primate restricted *Alu* elements. Despite the absolute numerical difference of editing between species, the genetics (MDA5 dependence) and transcriptional consequences (interferonopathy) of loss of function mutations in human *ADAR* are highly conserved with the features of loss of *Adar1*, either completely or the specific inactivation of editing activity, in mouse [30, 31, 62]. The phenotypic similarity suggests that, at least for ADAR1 substrates, the loss of editing of *Alu* elements is not a human/primate constrained driver of innate immune activation. Rather, the genetic results indicate that the consequences of a loss of ADAR1 activity are most likely due to species conserved secondary structures formed by unedited dsRNA that can be bound by MDA5, rather than species unique substrates [35].

A-to-I editing has been postulated to be a mechanism to fine-tune and diversify the output of the genome [1, 2]. The genetic evidence and analysis of the *Adar1*^*E861A/E861A*^*Adarb1*^*−/−*^ animals we provide indicates that mice tolerate being editing deficient surprisingly well, once MDA5-mediated self-sensing of dsRNA is prevented and the single edited site within GRIA2 is provided genomically. The in vivo result demonstrates that ADAR1 and ADAR2’s physiological functions are restricted to distinct pathways despite a significant fraction of editing being mediated interchangeably by either ADAR1 or ADAR2, particularly in the brain. It was particularly unexpected that strongly edited, evolutionarily conserved sites within coding regions do not appreciably affect development or lifespan of the mouse. Furthermore, as most editing occurs at a frequency of less than 20%, it is likely stochastic and is not required for normal mammalian development and homeostasis. Our data do not, however, rule out the possibility of more subtle phenotypic consequences of these editing sites under certain conditions. The genetic result indicates two distinct sets of physiologically essential editing events in vivo: (a) the recoding of the GRIA2 Q/R site by ADAR2 and (b) the unedited transcripts that become MDA5 substrates in the absence of ADAR1-mediated editing. The identity of the substrates that become immunogenic in the absence of ADAR1-mediated editing is an open question and remains a topic of intense interest. The current hypothesis is that they are present within the hyperedited transcript population; however, the number and identity of those that are immunogenic remains to be directly shown. Collectively, these critical events likely comprise a small subset of the editome and for these essential sites ADAR1 and ADAR2 are non-redundant and do not compensate for each other. These results demonstrate that in vivo biologically essential protein recoding mediated by A-to-I editing is an exception in mammals.

## Conclusions

A-to-I editing is one of the most common modifications in the mammalian transcriptome. Despite its abundance, our knowledge of the physiological functions of the vast majority of editing events is unknown. While mapping of the numbers and extent of A-to-I editing in multiple species is approaching saturation, only a handful of substrates have been characterized to date. To address this knowledge gap, we have now generated and characterized mice globally lacking A-to-I editing by crossing ADAR1-editing-deficient animals (*Adar1*^*E861A/E861A*^*Ifih1*^*−/−*^) with rescued *Adarb1*^*−/−*^*Gria2*^*R/R*^ animals. Unexpectedly, mice completely lacking A-to-I editing are strikingly normal when provided with the respective rescue alleles. The absence of additional phenotypes in the compound editing-deficient mice demonstrates that the physiologically essential functions of ADAR1 and ADAR2 do not intersect, despite a substantial degree of overlapping editing capacity by both enzymes. While A-to-I editing has long been associated with protein recoding and proteome diversification, physiologically essential protein recoding is an extremely rare (singular) event in the mouse. An implication of these findings is that a significant proportion of A-to-I editing may be stochastic and its global absence does not result in profound phenotypic consequence to a whole organism in vivo.

## Methods

### Animals

*Adar*^*E861A/+*^ (*Adar1*^*E861A/+*^; MGI allele: *Adar*^*tm1.1Xen*^; MGI:5805648) [30], *Ifih1*^*−/−*^ (MGI: *Ifih1*^*tm1.1Cln*^; MGI:3663677) [30, 63], *Adarb1*^*−/−*^ (*Adar2*^*−/−*^; MGI: *Adarb1*^*tm1.1Phs*^; MGI:2178079) [25], and *Gria2*^*R/R*^ (MGI: *Gria2*^*tm1.1Phs*^; MGI:2178125) [25, 64] mice were on a backcrossed C57BL/6 background as previously described. Animals were housed under standard SPF conditions with food and water ad libitum. Weaning weights were recorded on the day of weaning (~ 20–22 days of age). Nuclear magnetic resonance (NMR) relaxometry (EchoMRI) was performed on 12-week-old male animals of the indicated genotype, including 12-week-old male wild-type (WT) C57BL/6 animals bred and housed in the same facility, as directed by the manufacturer. For histopathology, 3 male *Adar1*^*E861A/+*^*Ifih1*^*−/−*^*Adarb1*^*+/−*^*Gria2*^*R/R*^ (dHet) and *Adar1*^*E861A/E861A*^*Ifih1*^*−/−*^*Adarb1*^*−/−*^*Gria2*^*R/R*^ (dKO) at ~ 20–25 weeks of age were assessed. Tissue collection and histology was performed by the Australian Phenomics Network Histopathology and Organ Pathology Core, University of Melbourne, on tissue listed in the report. The samples were genotype blinded to the pathologists, and sections were assessed by independent pathologists. The full pathology report is available in Additional file 2: Dataset S1.

### Cell counts and flow cytometry analysis of peripheral blood

Peripheral blood was analyzed on a hematological analyzer (Sysmex KX-21 N, Roche Diagnostics). Single cell suspensions from the BM, spleen, and thymus were prepared by passing through a 23G needle (BM) or crushing through a 40-μm cell strainer (spleen/thymus) [38]. Antibodies against murine B220 (APC-eFluor780), CD11b/Mac1 (PE), Gr1 (PE-Cy7), F4/80 (APC), CD4 (eFluor450), and CD8 (PerCP-Cy5.5) were all obtained from eBioscience [30, 38]. Cells were analyzed on a BD LSRIIFortessa (BD Biosciences). Results were analyzed with FlowJo software version 10.0 (Treestar).

### qRT-PCR and Sanger sequencing

Total RNA was isolated from the whole brain of 12-week-old male mice of the indicated genotypes. The tissues were isolated, flash frozen in liquid nitrogen, and then homogenized in Trisure reagent using IKA T10 basic S5 Ultra-turrax Disperser. RNA was extracted using Direct-Zol columns (Zymo Research) as per the manufacturer’s instructions. Complementary DNA (cDNA) was synthesized using Tetro cDNA synthesis kit (Bioline) with a *Htr2c*-specific RT primer (5′-TGTCAACGGGATGAAGAATGCC). The previously defined edited sites in *Htr2c* were identified by Sanger sequencing of PCR product (not further purified or cloned) by the Australian Genome Research Facility, Melbourne (forward primer 5′-GGCCAGCACTTTCAATAGTCGTG, reverse primer 5′-CAATCTTCATGATGGCCTTAGTCC).

### RNA-seq samples and library preparation

Total RNA was isolated from the whole brain from three independent biological replicates from 12-week-old male *Adar1*^*E861A/+*^*Ifih1*^*−/−*^*Adarb1*^*+/−*^*Gria2*^*R/R*^, *Adar1*^*+/+*^*Ifih1*^*−/−*^*Adarb1*^*−/−*^*Gria2*^*R/R*^, and *Adar1*^*E861A/E861A*^*Ifih1*^*−/−*^*Adarb1*^*−/−*^*Gria2*^*R/R*^ mice (*n* = 3/genotype). The tissues were isolated, flash frozen, and then homogenized in Trisure reagent using IKA T10 basic S5 Ultra-turrax Disperser. RNA was extracted using Direct-Zol columns (Zymo Research) as per the manufacturer’s instructions. Post ribosome-depleted RNA was purified and subjected to indexing and library preparation using the Kapa Stranded RNA-seq Library Preparation Kit (Kapa Biosystems) [38] and sequenced using the Illumina platform with 150-bp paired-end reads by Novogene (Novogene (HK), Hong Kong).

### RNA-seq analysis

Reads from two different technologies were used in the analysis: *Adar1*^*E861A/+*^*Ifih1*^*−/−*^*Adarb1*^*+/−*^*Gria2*^*R/R*^ (dHet), *Adar1*^*+/+*^*Ifih1*^*−/−*^*Adarb1*^*−/−*^*Gria2*^*R/R*^ (Adar2^*−/−*^), and *Adar1*^*E861A/E861A*^*Ifih1*^*−/−*^*Adarb1*^*−/−*^*Gria2*^*R/R*^ (dKO) (150-bp paired end) and the previously published *Adar1*^*E861A/E861A*^*Ifih1*^*−/−*^ (E861A) and *Adar1*^*+/+*^*Ifih1*^*−/−*^ (WT) samples (GSE94387) (75 bp paired end).

### Pre-processing

Sequenced reads (150 bp) were trimmed for adaptor sequence and low-quality reads using fastp (v 0.19.5) [65]. Parameters: --trim_front1 10 --trim_front2 10. Sequenced reads (75 bp) from GSE94387 were trimmed for adaptor sequence and low-quality reads using (v 0.19.5) [65]. Parameters: --trim_front1 10 --trim_front2 10 --trim_tail1 1 --trim_tail2 1. Reads mapping to rRNA were removed using Bbmap (parameters: bbsplit.sh minratio = 0.56 minhits = 1 maxindel = 16000) [BBMap – Bushnell B. – sourceforge.net/projects/bbmap/].

### Gene expression

For transcriptome analysis, trimmed reads were aligned using Salmon [66] (version v0.11.3) against mm10 (annotation: gencode.mm10.vM14.annotation.gtf).

Differential gene expression analysis was performed using the Degust analysis tool (http://victorian-bioinformatics-consortium.github.io/degust/). Briefly, genes were only considered with count > 3 and CPM > 1 in at least 3/3 samples of a given genotype. Normalized read counts (moderated log counts per million) and differential expression were generated using edgeR [67]. Each comparison (E861A vs WT, A2KO vs Het, DKO vs Het) was performed separately. See Additional file 3: Dataset S2.

### QuSAGE gene set testing

Quantitative Set Analysis for Gene Expression (QuSAGE) [68] of the consensus interferon-stimulated gene (ISG)/cytokine signature defined by Liu et al. [20] was performed on using the gene expression data. See Additional file 4: Dataset S3.

### Editing analysis

#### Mapping

Trimmed reads were aligned to the MM10/GRCm38 reference genome with transcript annotation (gencode.mm10.vM14.annotation.SEQINS.gtf) with STAR (version 2.6.0c) [69] using the following parameters: --outFilterType BySJout --outSAMattributes NH HI AS NM MD --outFilterMultimapNmax 20 --outFilterMismatchNmax 999 --outFilterMismatchNoverReadLmax 0.04 --alignIntronMin 20 --alignIntronMax 1000000 --alignMatesGapMax 1000000 --alignSJoverhangMin 8 --alignSJDBoverhangMin 1 --sjdbScore 1 --sjdbOverhang 149. Duplicate reads were marked Picard [“Picard Toolkit.” 2019. Broad Institute, GitHub Repository. http://broadinstitute.github.io/picard/; Broad Institute].

#### Known sites

A database of 57,077 murine editing sites was compiled from published databases (RADAR [8]), publications [4, 30, 43], and unpublished murine datasets (JH-F, AMC, and CRW) and the datasets assessed for editing at these sites. Sites were marked as hyperedited if there were > 10 editing sites within 100 bp, and no consideration was made about editing level or if editing occurred in this dataset [10]. See Additional file 5: Dataset S4A.

#### Calling known sites

Editing calling of known sites (RNA vs mm10) was performed using JACUSA 2.0.0-RC5 (70) (https://github.com/dieterich-lab/JACUSA): parameters used: -F 1024 -filterNH_ 99, -filterNM_ 99, -c 3 -P RF-FIRSTSTRAND. Briefly, call-1 was used to determine the RNA editing level for all known sites for each individual sample replicate. Duplicate reads were removed. For sites not called by JACUSA, we added read depth calculated by samtools pileup to reflect the sequence coverage at those positions. The editing rate for each genotype was calculated as the sum of edited reads for three replicates/total read depth for all three replicates. Sites required ≥ 50 read coverage in all samples (a combined read coverage of ≥ 50 for all genotypes was required) of the comparison and an editing rate of ≥ 0.01 (≥ 1%) in the WT and dHet to be considered. See Additional file 5: Dataset S4A.

#### Differential editing of known sites

Calling of differential editing in known sites across genotypes was performed using JACUSA 2.0.0-RC5 [70]. Briefly, call-2 was used to determine the difference in editing level for all known sites (all replicates of genotype A vs all replicates of genotype B). Duplicate reads were removed. Sites required ≥ 50 read coverage and an editing rate of ≥ 0.01 (≥ 1%) to be considered. See Additional file 5: Dataset S4A.

#### Calling novel editing sites

Editing calling of novel sites was performed using JACUSA 2.0.0-RC5 [70]. Briefly, call-2 was used to determine the RNA editing level for all sites within 5 kb of all Ensembl genes (ensembl_genes_96) using all replicates for each genotype. Duplicate reads were removed, and min coverage of 3 per sample (parametres used: -F 1024 -filterNH_99, -filterNM_ 99, -c 3 -P RF-FIRSTSTRAND). A site was considered edited if score z > 5; all sites with score z > 5 in the DKO were removed after manual assessment. See Additional file 6: Dataset S4B.

#### Annotation

Editing sites were annotated with gene, gene part (promoter, Exon, intron, 3′ UTR, or intergenic) using Goldmine [71]. B1 and B2 SINE annotation (mm10) was from UCSC rmsk table [https://www.ncbi.nlm.nih.gov/pubmed/23155063]. See Additional files 5, 6, and 7: Datasets S4A, S4B, S5.

#### Sequence logos

Sequence logos were generated using ggseqlogo [72].

#### Datasets

All datasets related to this work are deposited in GEO. Dataset accession number: GSE132214 (https://www.ncbi.nlm.nih.gov/geo/query/acc.cgi?acc=GSE132214).

#### Statistical analysis

For biological experiments, the significance of results was analyzed using the one-way or two-way ANOVA with multiple comparison corrections unless otherwise stated; calculated as Prism software, *P* < 0.05 was considered significant. Data are presented as mean ± SEM unless otherwise stated.

## Supplementary information

**Additional file 1: Figure S1 (related to Figure 3).** Comparison of gene expression signatures by genotype; data from Panel 3A. Analysis of transcriptional signatures in the 12 week old male brain of each genotype. n=3 independent samples per genotype. The increased expression of the transcripts highlighted in blue is shared between murine and human ADAR1 mutants. (**A**) Y-axis has the gene expression comparison of the Adar2-/- vs the dHet; x-axis has the gene expression comparison of the Adar1E861A/E861A vs WT. Gene expression changes dependent on Adar1 loss occur on the x axis, those dependent on the loss of Adar2 on the y axis. (**B**) Adar2-/-compared to Adar1E861A/E861A Adar2-/- (dKO); (**C**) Adar1E861A/E861A compared to Adar1E861A/E861A Adar2-/- (dKO). **Figure S2.** Comparison of the gene expression signatures by genotypes; data derived from comparisons in Panel 2A. **Figure S3** **(related to Figure 4).** Altered sites identified in analysis of Adar1E861A/E861A Adarb1-/- (dKO); related to Panel 4B. Analysis of sites identified as altered compared to ref seq or batch control in the dKO samples. Individual sites with IGV screenshots and the full list of sites with variants identified in analysis of the double KO samples.

**Additional file 2 Dataset S1.** Full histopathology report from Adar1E861A/+Ifih1-/-Adarb1+/-Gria2R/R (dHet) and Adar1E861A/E861AIfih1-/-Adarb1-/-Gria2R/R (dKO).

**Additional file 3: Dataset S2.** RNA-seq data used for differential gene expression analysis. Samples=12 week old male whole brain; n=3 per genotype. Related to Fig 2 and Fig S2.

**Additional file 4: Dataset S3.** QuSAGE pathway analysis of gene expression datasets. Samples= 12 week old male whole brain; n=3 per genotype. Related to Fig 2 and Fig S2.

**Additional file 5: Dataset S4A.** Editing analysis of the known sites. Related to Fig. 3 and Fig. 4.

**Additional file 6:** **Dataset S4B.** De novo discovery of RNA editing sites in each genotype using JACUSA2.0.0 (transcriptome comparison to C57Bl/6 reference genome). Related to Fig 3 and Fig 4.

**Additional file 7:** **Dataset S5.** ADAR1 and ADAR2 specific editing events – frequency of editing. Related to Fig. 4c.

**Additional file 8:** Review history.

## Data Availability

All datasets described in this work are deposited in GEO under accession code GSE132214 [73]. Mouse strains are available from the Australian Phenome Bank (https://pb.apf.edu.au/phenbank/homePage.html).
