# Supplementary material for: The majority of A-to-I RNA editing is not required for mammalian homeostasis
Source: Genome Biol. 2019 Dec 9;20:268. doi: 10.1186/s13059-019-1873-2 (PMC6900863; doi:10.1186/s13059-019-1873-2)
Supplement: Supplementary file 1 — Additional file 1: Figure S1 (related to Figure 3). Comparison of gene expression signatures by genotype; data from Panel 3A. Analysis of transcriptional signatures in the 12 week old male brain of each genotype. n=3 independent samples per genotype. The increased expression of the transcripts highlighted in blue is shared between murine and human ADAR1 mutants. (A) Y-axis has the gene expression comparison of the Adar2-/- vs the dHet; x-axis has the gene expression comparison of the Adar1E861A/E861A vs WT. Gene expression changes dependent on Adar1 loss occur on the x axis, those dependent on the loss of Adar2 on the y axis. (B) Adar2-/-compared to Adar1E861A/E861A Adar2-/- (dKO); (C) Adar1E861A/E861A compared to Adar1E861A/E861A Adar2-/- (dKO). Figure S2. Comparison of the gene expression signatures by genotypes; data derived from comparisons in Panel 2A. Figure S3 (related to Figure 4). Altered sites identified in analysis of Adar1E861A/E861A Adarb1-/- (dKO); related to Panel 4B. Analysis of sites identified as altered compared to ref seq or batch control in the dKO samples. Individual sites with IGV screenshots and the full list of sites with variants identified in analysis of the double KO samples. [file 13059_2019_1873_MOESM1_ESM.pdf]

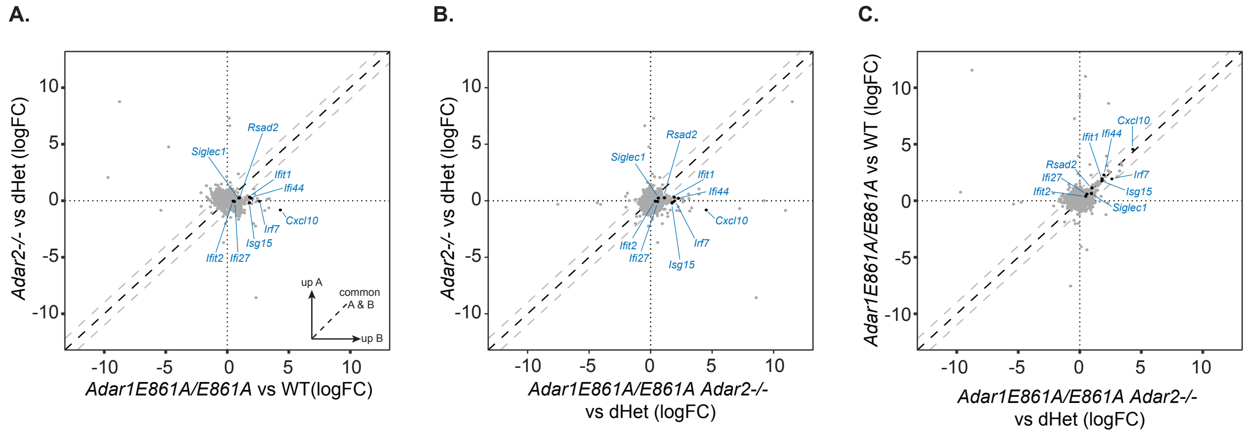

Additional file 1: Figure S1 (related to Figure 3). Comparison of gene expression signatures by genotype; data from Panel 3A. Analysis of transcriptional signatures in the 12 week old male brain of each genotype. n=3 independent samples per genotype.
The increased expression of the transcripts highlighted in blue is shared between murine and human ADAR1 mutants. (**A**) Y-axis has the gene expression comparison of the Adar2-/- vs the dHet; x-axis has the gene expression comparison of the Adar1E861A/E861A vs WT. Gene expression changes dependent on Adar1 loss occur on the x axis, those dependent on the loss of Adar2 on the y axis. (**B**) Adar2-/- compared to Adar1E861A/E861A Adar2-/- (dKO); (**C**) Adar1E861A/E861A compared to Adar1E861A/E861A Adar2-/- (dKO).

Genotype	*AdarE861A/+ Ifih1-/- Adarb1+/- Gria2 R/R* (n=31)	*Adar+/+ Ifih1-/- Adarb1-/- Gria2 R/R* (n=22)	*AdarE861A/+ Ifih1+/- Adarb1-/- Gria2 R/+* (n=11)	*AdarE861A/+ Ifih1-/- Adarb1-/- Gria2 R/+* (n=22)	*AdarE861A/+ Ifih1-/- Adarb1-/- Gria2 R/R* (n=61)	*AdarE861A/E861A Ifih1-/- Adarb1-/- Gria2 R/+* (n=10)	*AdarE861A/E861A Ifih1-/- Adarb1+/- Gria2 R/R* (n=28)	*AdarE861A/E861A Ifih1-/- Adarb1-/- Gria2R/R* (n=68)
AdarE861A/+ Ifih1-/- Adarb1+/- Gria2 R/R (n=31)		0.11	***	***	0.12	***	0.83	0.25
Adar+/+ Ifih1-/- Adarb1-/- Gria2 R/R (n=22)			***	***	0.82	***	0.044	0.34
AdarE861A/+ Ifih1+/- Adarb1-/- Gria2 R/+ (n=11)				0.10	***	0.98	***	***
AdarE861A/+ Ifih1-/- Adarb1-/- Gria2 R/+ (n=22)					***	0.12	***	***
AdarE861A/+ Ifih1-/- Adarb1-/- Gria2 R/R (n=61)						***	0.035	0.53
AdarE861A/E861A Ifih1-/- Adarb1-/- Gria2 R/+ (n=10)							***	***
AdarE861A/E861A Ifih1-/- Adarb1+/- Gria2 R/R (n=28)								0.15
AdarE861A/E861A Ifih1-/- Adarb1-/- Gria2R/R (n=68)								

*** $P<0.0001$

chr11:3159799 : Sfi1 intron. SNP, with ~ 100% reads - ??.

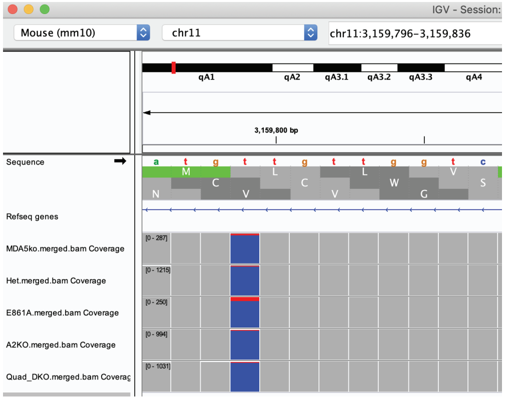

chr18:10020838 : Usp14, intron, low coverage in IGV
(Due to read pair overlapping the region but not aligning in that position).

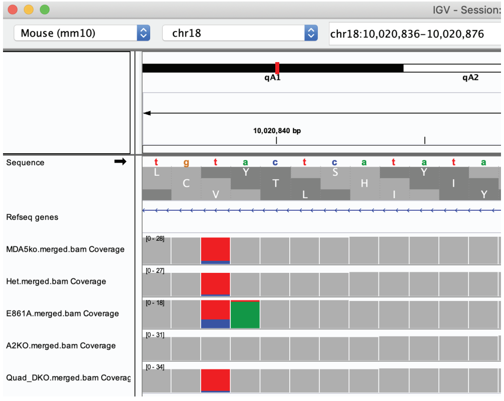

chr3:80706912 : Gria2 site

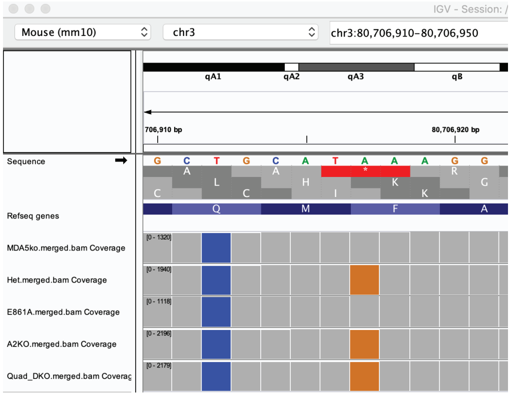

chr5:121803528 : Atxn2. Intron, only 1 read edited. Artifact.

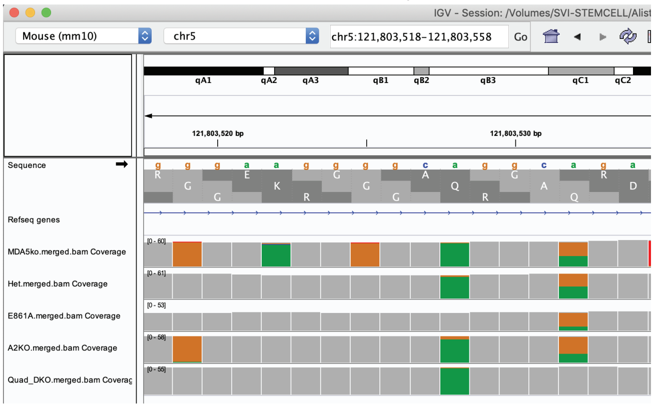

chr2:174046116 : Intergenic. SNP.
chr2:174046360 : Intergenic. SNP. In all genotypes.
chr3:80706912 : Gria2 site.
chr11:3159799 : Sfi1 intron. SNP, with ~ 100% reads - ??.
chr16:33476775 : Zfp148, intron. Low coverage in IGV (Due to read pair overlapping the region but not aligning in that position).
chr1:171243874: Ndufs, intron, low coverage in IGV (Due to read pair overlapping the region but not aligning in that position).
chr17:39844592 : Rn45s. Artifact (T in direction of transcription).
chr4:108492563 : Zcchc11(Tut4), intron, low coverage in IGV (Due to read pair overlapping the region but not aligning in that position).
chr11:107098345 : Bptf, intron, low coverage in IGV (Due to read pair overlapping the region but not aligning in that position).
chr2:6544756 : Celf2. Region of TTTTTTTT. Likely sequencing/alignment error.
chr18:10020838 : Usp14, intron, low coverage in IGV (Due to read pair overlapping the region but not aligning in that position).
chr18:24790652 : Fhod, intron, low coverage in IGV (Due to read pair overlapping the region but not aligning in that position).
chr7:109952366 : Dennd5a intron, low coverage in IGV (Due to read pair overlapping the region but not aligning in that position).
chr5:121803528 : Atxn2. Intron, only 1 read edited. Artifact.
chr4:151711818 : Camta1, intron, low coverage in IGV (Due to read pair overlapping the region but not aligning in that position).
chr13:49048658 : Wnk2, intron, 2 reads edited at position. TTCCTTCCTT.
chr7:19200367 : On reverse strand. Adjacent to 2bp INDEL in majority DKO reads. Artifact.
chr11:75642529 : Inpp5k, intron, low coverage in IGV (Due to read pair overlapping the region but not aligning in that position), 1 edited read.
chr5:123554088 : Vps33a, intron, 2 reads.

Additional FIle 1: Fig S3 (related to Figure 4). Altered sites identified in analysis of Adar1E861A/E861A Adarb1-/- (dKO); related to Panel 4B. Analysis of sites identified as altered compared to ref seq or batch control in the dKO samples. Individual sites with IGV screenshots and the full list of sites with variants identified in analysis of the double KO samples.
