# Supplementary material for: The majority of A-to-I RNA editing is not required for mammalian homeostasis
Source: Genome Biol. 2019 Dec 9;20:268. doi: 10.1186/s13059-019-1873-2 (PMC6900863; doi:10.1186/s13059-019-1873-2)
Supplement: Supplementary file 2 — Additional file 2 Dataset S1. Full histopathology report from Adar1E861A/+Ifih1-/-Adarb1+/-Gria2R/R (dHet) and Adar1E861A/E861AIfih1-/-Adarb1-/-Gria2R/R (dKO). [file 13059_2019_1873_MOESM2_ESM.pdf]

Contacts:

Australian Phenomics Network
Histopathology and Organ Pathology
The University of Melbourne
PARKVILLE, VIC 3010

9.1 Histopathology Report

Case Number
APN18/039 St. Vincent's Institute of Medical Research (Carl Walkley)

Registration Date
Thu 13/12/2018

Animal Details
#629 **(Genotype: $Adar1^{E861A/E861A}Ifih1^{-/-}Adarb1^{-/-}Gria2^{R/R}$)**
DOB: 05/07/2018, 23 weeks old, Male, 30.8g, Black

#634 **(Genotype: $Adar1^{E861A/E861A}Ifih1^{-/-}Adarb1^{-/-}Gria2^{R/R}$)**
DOB: 12/07/2018, 22 weeks old, Male, 28.8g, Black

#635 **(Genotype: $Adar1^{E861A/E861A}Ifih1^{-/-}Adarb1^{-/-}Gria2^{R/R}$)**
DOB: 12/07/2018, 22 weeks old, Male, 31.8g, Black

#640 **(Genotype: $Adar1^{E861A/+}Ifih1^{-/-}Adarb1^{-/-}Gria2^{R/R}$)**
DOB: 21/07/2018, 21 weeks old, Male, 27.8g, Black

#641 **(Genotype: $Adar1^{E861A/+}Ifih1^{-/-}Adarb1^{-/-}Gria2^{R/R}$)**
DOB: 21/07/2018, 21 weeks old, Male, 31.0g, Black

#642 **(Genotype: $Adar1^{E861A/+}Ifih1^{-/-}Adarb1^{-/-}Gria2^{R/R}$)**
DOB: 21/07/2018, 21 weeks old, Male, 32.4g, Black

DoD / Necropsy
Thu 13/12/2018

Death

Origin
St. Vincent's Institute of Medical Research

Treatment
Knock-out
6 live males, currently 8-15 weeks old; 3 controls and three are the genotype of interest).
(C. Walkley 03/12/2018)

None are true WT. The control is a heterozygous allele which we know is normal based on lifespan, breeding, blood analysis, body composition and weights. This is because the 3 mice of interest in the cohort are a quadrupole mutation and it is not possible to generate WT mice from the breeding.
(C. Walkley 13/12/2018)

Species / Breed / Strain
Adar1 E861A/E861A Ifih1-/- Adar2-/- Gria2 R/R

Animal Health Facility
St. Vincent's Bioresources Centre
POSITIVE for Helicobacter hepaticus, Helicobacter spp, Pasteurella pneumotropica, Pasteurellaceae group, Pinworm - Syphacia obvelata, Pinworm - Syphacia spp, Chilomastix bettencourti, Entamoeba muris, Tritrichomonas muris, Mouse Norovirus, and other significant organisms

Organs Examined
Adrenal glands, Bladder, Bone marrow, Brain, Cecum, Colon, Duodenum, Epididymes, Eyes, Gall bladder, Harderian glands, Head, Heart, Hind leg (Long bone, Bone marrow, Synovial joint, Skeletal muscle), Ileum, Jejunum, Kidney, Liver, Lungs, Mesenteric lymph node, Pancreas, Penis, Preputial gland, Prostate glands, Salivary glands and Regional lymph nodes,

Seminal vesicles, Skin, Spinal cord, Spleen, Sternum, Stomach, Tail, Testes, Thymus, Thyroids, Trachea

Macroscopic Observations

Date of transport to the APN: December 13th 2018
Courier/Transportation details:

Body Condition Scoring (BCS): Scale of 1-5
5: The mouse is obese, and bones cannot be felt at all
4: The mouse is well-fleshed, and bones are barely felt
3: The mouse is in optimal condition. The bones are palpable but not prominent
2: The mouse is thin, and bones are prominent
1: Muscle wasting is advanced; fat deposits are gone, and bones are very prominent.

At the time of necropsy, the animals appeared well nourished, well groomed, active/curious and healthy with normal movement and gait (BCS of all animals=3). There were no observable dermal lesions and no nasal/ocular discharges. The gastrointestinal tract contained ample ingesta- the ileal region of animals #640 and #642 felt rigid, and that the gastrointestinal tract of all 6 animals was notably longer than the typical mouse. The thoracic and other abdominal viscera showed no macroscopic abnormalities.
Please refer to individual animals for more macroscopic details.

Blood Report:
Most readings are within the normal mouse reference intervals.
#629: Low platelet count, as the MPV was within range, this is unlikely to be significant. Decreased platelet counts are a common haematologic finding in mice. However this change is often secondary to blood collection difficulties rather than a true decrease in platelet counts. Mouse platelets readily aggregate and instrument generated platelet counts will underestimate true platelet counts in the presence of platelet clumping. In addition, mouse platelet clumps are counted as eosinophils by some automated haematology analysers.
#634: Elevated number of lymphocytes
#635: Elevated WBC count (neutrophils and lymphocytes)
#640: Elevated RBC count, likely associated elevated HGB
All samples show-
Elevated HCT, likely to be a result of mild dehydration.
Elevated eosinophil counts, a common occurrence in mice.
Due to blood collection difficulties, no results can be given for sample #642.
For more details please see the accompanying APN18/039SVI(C. Walkley) Blood Report.

Microscopic Observations

SUMMARY of micromorphological changes:
Experimental treatment groups were not defined and the assessment was completed blind.

All animals showed age related, and/or incidental micromororphological changes (eg. inflammation, degeneration) most notably within the testes, stomach, large intestine (cecum and colon), kidneys, salivary glands, lungs, heart and skin (various regions).

Within the small intestine, animals #629, #634 and #635 showed prominent clusters of apoptotic cells in the gut mucosa. The submandibular glands of these animals also showed less prominent acini. The significance of these changes are unknown.

Also within the small intestine, animals #640, #641 and #642 showed disruption of the typical mucosal micromorphology with mild enterocyte hyperplasia, most notably of goblet cells, prominent immune cells within the lamina propria, and cytoplasmic vacuolation of apical cells.

For more microscopic details, please refer to individual animals in the report below.

Notes:
(1) Accumulation of leukocytes and other cells are common nonneoplastic lesions in many tissues. The term “inflammation” is used when the cell (leukocyte) accumulations are part of an active inflammatory process (typified by concurrent features such as vascular changes, necrosis, fibrosis, and/or tissue disruption). In contrast, cell “infiltration” is used when the cell (e.g., lymphocyte) accumulations are present in tissue without other disruption or pathology.
(2) Focal inflammatory cell aggregates consisting of mononuclear, polymorphonuclear, and/or histiocytic cells are frequently observed in ageing mice (Maranport RR. 1999. Pathology of the Mouse.). These can be present as lymphoid aggregates found in various tissues including the renal pelvis, bladder, lungs, liver (Pettan-Brewer C and Treuting PM. 2011. Practical pathology of aging mice.) and salivary glands (Haines DC, Chattopadhyay S and Ward JM. 2001. Pathology of Aging B6;129 Mice).
(3) Reactive lymph nodes are defined as mild follicular hyperplasia, germinal centre formation and occasional sinus histiocytosis - a common finding in mice.
(4) Hyperplastic lymph node follicles are identified by an increase in number and size of follicles and conversion to secondary follicles. Hyperplasia of the paracortex is characterized by an increase in the cell density and, depending on the degree of hyperplasia, an increase in the paracortical area.
(5) Mild extramedullary haematopoiesis (EMH) identified in the red pulp of all the spleens, a common finding in the mouse. EMH consists of erythroid precursors, myeloid precursors, megakaryocytes or all three. While some degree of extramedullary haematopoiesis is present in normal rodents, especially in mice, increased extramedullary haematopoiesis can result from haematotoxin insult, systemic anaemia, and infections elsewhere in the body. (Suttie AW. 2006. Histopathology of the spleen).
(6) Hyperplasia of the stomach epithelium is defined as an increase in the number of cells in one or more epithelial strata (basal, spinous, or granulosum). Hyperkeratosis (increased thickness of the stratum corneum) is often seen when hyperplasia of the mucosa is present. Hyperplasia of the nonglandular stomach is often characterized as a uniform thickening of all layers of the epithelium without upward or downward projections. However, in more severe cases there are convolutions of the epithelium with downward projections of rete peg-like structures. Focal upward proliferation into the lumen mimicking papilloma may be present in more severe cases of focal hyperplasia and are described as papillary hyperplasia (Kooistra LH, Nyska A. 2014. Stomach, Forestomach, Epithelium – Hyperplasia. In: National Toxicology Program Nonneoplastic Lesion Atlas).
(7) Thymic cysts in the rodent represent either a dilatation of thymic tubular structures or remnants of the thymopharyngeal duct. They are common findings in the involuted and/or atrophied thymus glands of rats and mice. Thymic cyst formation becomes more prominent with age and is associated with involution. (Hobbie K, Elmore SA and Kolenda-Roberts HM. 2015. Thymus – Cyst. In: National Toxicology Program Nonneoplastic Lesion Atlas).
(8) Valvular myxomatous changes or degeneration can be an age-related spontaneous or chemical-induced change. The lesion is characterized by focal or segmental thickening of the subendocardium in the valve leaflets and expansion of the spongiosa of the valve leaflet with extracellular fibromyxoid material composed predominantly of glycosaminoglycans. Occasionally, fibrin deposits or thrombi and collections of neutrophils or mononuclear cells are seen (Johnson CL, Nyska A. 2017. Heart, Valve, – Degeneration. In: National Toxicology Program Nonneoplastic Lesion Atlas).
(9) Germ cell degeneration of the testes is a nonspecific term that generally includes a number of degenerative features, such as tubular vacuolation, partial depletion of germ cells, degenerating (multinucleated or apoptotic) germ cells, and disordered arrangement of the germ cell layers. Chemically induced germ cell degeneration can be multifocal in distribution, but it is most often a bilateral lesion that affects most of the seminiferous tubules to varying degrees. It can also be an incidental background finding in rats and mice of any age, but the incidence increases with age (G.Wilson and K. Y. Cimon “Nonneoplastic Lesion Atlas, Uterus, Testis, Germ cell – Degeneration”, 2015).
(10) Lymphocyte apoptosis is characterized by cell shrinkage, nuclear pyknosis and fragmentation with apoptotic bodies. This type of cell death normally occurs within the germinal centres of secondary follicles where it is an important homeostatic mechanism (Hobbie K, Elmore SA and Kolenda-Roberts HM. 2015. Lymph Node – Apoptosis, Lymphocyte. In: National Toxicology Program Nonneoplastic Lesion Atlas).

#629

Macro Observations

Tail suspension test for neurological defects-negative
BCS: 3

Dentition, tongue and oral cavity was unremarkable
Testes: 10x8x6mm and 9x6x5mm, asymmetrical
Spleen: 23x8x2mm
Kidneys: 18x10x6mm, symmetrical
Thymus: 7x7x2mm
Lungs inflated
Heart: 14x10x8mm
Brain: 15x10x5mm
Pituitary gland identified, macroscopically normal
Tail length: 90mm (straight)
Head harvested for evaluation of auditory and vestibular structures
Hind leg-Bone marrow smear

Micro Observations

Marrow smear: Examination of the smear showed representative cells from the myeloid and erythroid series. Occasional cells from the lymphoid series. Occasional and unremarkable megakaryoblasts.
(71190)

Peripheral blood smear: Examination of the smear showed red blood cells (majority of cells shown), numerous white blood cells including lymphocytes, segmented neutrophils, monocytes and platelets (clumps). No discernible morphological changes or detectable parasites.
(71191)

Micromorphological changes-
Stomach: limiting ridge shows focal epithelial hyperplasia, a nearby small submucosal aggregate of cellular debris (possibly mineralisation) and cluster of mononuclear infiltrates (71177), likely to be incidental findings
-Pathology to comment-
Small intestine: mucosa shows prominent clusters of apoptotic cells (71177, 71164, 71162), significance unknown
-Pathology to comment-
Large intestine: numerous intraluminal protozoan parasites (71162), considered an incidental finding
Kidneys: few small foci of perivascular lymphocytic inflammation (71169), considered an age related finding
Salivary glands: mild multifocal perivascular lymphocytic inflammation (71172, 71173, 71176), considered an age related finding
Salivary glands: acini cells of the submandibular gland appear less prominent (71172, 71173, 71176), significance unknown
-Pathology to comment-
Lungs: few small foci of perivascular lymphocytic inflammation (71166, 71180), considered an incidental finding
Thymus: a small cyst can be seen within the cortex (71166), a common finding in the mouse
Heart: mild myxomatous valvular changes (thickened leaflets) (71180, 71181), considered an age related finding

Testes/Epididymes

Section shows typical convoluted seminiferous tubules at various stages of cycle surrounded by the tunica albuginea. Within the tubules, unremarkable spermatogenic cells including, Sertoli cells, spermatogonia, developing spermatocytes and spermatids. Typical interstitial Leydig cells.
Section also shows unremarkable vas deferens with typical intraluminal sperm.
The architecture of the epididymis is typical, with numerous intraluminal elongated spermatozoa.
(71179)
No lesions of significance

Seminal vesicles

Unremarkable tall columnar epithelium and folded mucosa.
Presence of typical intraluminal eosinophilic secretions.
(71172, 71173, 71176)
No lesions of significance

Prostate glands

Sections show unremarkable dorsal lateral/ventral/coagulating glands with typical intraluminal secretions.
(71172, 71173, 71176)
No lesions of significance

Penis/Preputial gland

Sections show typical penile structures including prepuce, glans, corpus cavernosum and urethra.
Typical preputial glands including basal and secretory cells.
(71171)
No lesions of significance

Urinary Bladder

Sections do not include urinary bladder.

Liver/Gall bladder

Section shows typical liver parenchyma including hepatocytes, Kupffer cells, portal triads and central veins.
Unremarkable Gall bladder.
(71170)
No lesions of significance

Stomach

Section shows representative fore and glandular portions of the stomach. Limiting ridge shows focal epithelial hyperplasia, a nearby small submucosal aggregate of cellular debris (possibly mineralisation) and cluster of mononuclear infiltrates. Likely to be incidental findings.
Section also includes pyloric sphincter and duodenal bulb.
(71177)

Comments:

Pathology to comment

Small Intestine (Duodenum, Jejunum & Ileum)/GALT

Sections show typical mucosal villi and submucosal layers. Mucosa shows conspicuous mitotic figures, a typical finding, and prominent clusters of apoptotic cells, significance unknown.
The Peyer’s patches display mild follicular hyperplasia with germinal centre formation, indicating a reactive state.
(71177, 71164, 71162)

Comments:

Pathology to comment

Cecum/Colon/GALT

Typical mucosal folds and submucosal layers and an occasional lymphoid cluster (Peyer's patch).
Numerous intraluminal protozoan parasites.
(71162)
No lesions of significance

Mesenteric lymph node

Sections show mesenteric lymph node with typical reactive micromorphology including mild follicular hyperplasia, germinal centre formation and mild sinus histiocytosis.
(71172, 71173, 71176)
No lesions of significance

Spleen

Mild follicular hyperplasia with germinal centre formation in the white pulp.
Moderate extramedullary haematopoiesis and hemosiderin laden macrophages identified in the red pulp.
(71172, 71173, 71176)
No lesions of significance

Pancreas

Sections show representative exocrine tissue (serous acini) and endocrine tissue (islets of Langerhans).
(71172, 71173, 71176)
No lesions of significance

Kidney

Sections show a cortex, medulla, and papilla. There is a uniform distribution of glomeruli and accompanying nephron components and the micromorphology of the tubules is unremarkable.
The cuboidal parietal cells lining the Bowman's capsule of the male kidneys are conspicuous.
Few small foci of perivascular lymphocytic inflammation, considered an age related finding.
Sections also include renal lymph nodes with typical reactive nodal histology and mild sinus histiocytosis.
(71169)
No lesions of significance

Adrenal glands

Sections show an adrenal gland with typical cortex/medulla micromorphology.
(71169)
No lesions of significance

Salivary glands and Regional lymph nodes

Sections show unremarkable sublingual and parotid glands.
Submandibular gland shows mild multifocal perivascular lymphocytic inflammation, considered an age related finding. Acini cells of the submandibular gland appear less prominent, significance unknown.
The regional lymph nodes display mild follicular hyperplasia with germinal centre formation, indicating a reactive state.
(71172, 71173, 71176)

Comments:

Pathology to comment

Thyroids

Normal lateral lobes of the thyroid gland with typical colloid secreting follicles lined by cuboidal epithelium. Sections also show a small sheet-like mass of polygonal cells, characteristic of the parathyroid gland.
(71166, 71180)
No lesions of significance

Trachea/Lungs

Sections show typical lung parenchyma/alveoli, bronchioles, and blood vessels.
Few small foci of perivascular lymphocytic inflammation, considered an incidental finding (71166, 71180).
Trachea with unremarkable mucosal epithelial lining and hyaline cartilage.
Oesophagus with typical features including stratified squamous epithelium.
(71166, 71180, 71181)
No lesions of significance

Thymus

Sections show typical medulla/cortex distribution and micromorphology.
A small cyst can be seen within the cortex (71166), a common finding in the mouse.
(71166, 71180, 71181)
No lesions of significance

Heart/chambers/vessels/valves

Typical expected observed in cardiac muscle, chambers, and greater vessels of the heart.
The cardiac muscle fibres demonstrated typical features including central nuclei, branching fibres and striations.
Mild myxomatous valvular changes (thickened leaflets) (71180, 71181), considered an age related finding.
(71166, 71180, 71181)
No lesions of significance

Skin

Typical dermal appendages and distribution. Unremarkable thin layer of striated muscle (panniculus carnosus).
(71174)
No lesions of significance

Tail

Sections show typical tail components including keratinized squamous epithelium, dense regular connective tissue, tendons, caudal vertebra, bone marrow, intervertebral disc, skeletal muscle, nerves and blood vessels.
(71167, 71178)
No lesions of significance

Eyes/Harderian glands

Section shows eyes with unremarkable retina, cornea, iris, ciliary body, lens, sclera and choroid.
Typical branched tubuloalveolar formation of the Harderian gland.
Section also includes portion of unremarkable optic nerve and extraocular muscles.
(71165)
No lesions of significance

Brain

Sections were prepared from the standard levels of the brain:

Level I forebrain: including cortex, corpus callosum, caudate putamen and lateral ventricles (Bregma 0.38mm)
Level II midbrain: including the hippocampus, thalamus, hypothalamus and lateral and third ventricles (Bregma -1.94mm)
Level III hindbrain: includes the cerebellum, pons and fourth ventricle (Bregma -5.80mm)

Sections of brain stained with Haematoxylin and Eosin, Luxol Fast Blue appear symmetrical with no ventricular dilation observed, unremarkable meninges and typical lamination.
The cerebellum appears symmetrical with typical architecture and Purkinje cells.
There was no obvious neuronal loss and the myelination appears normal.
(71161, 71189)

No lesions of significance

Comments:

Neuropathology to comment

Spinal cord

Representative thoracic and lumbar region of spinal cord, vertebral bone, striated muscle, peripheral nerves, brown adipose tissue, and bone marrow.
(71175)
No lesions of significance

Comments:

Neuropathology to comment

(Hind leg) Long bone/Bone marrow/Synovial joint/Skeletal muscle

Sections show unremarkable long bone, striated muscle, examples of nerve fascicles, fibrocartilage of the meniscus, synovial joint and bone marrow. The skeletal muscle shows consistent fibre size with peripheral nuclei.
(71167, 71178)
No lesions of significance

Head

Multiple levels through the head demonstrate dermal appendages, nasal cavity, oral cavity, teeth and tongue including muscle bundles. Sections also show unremarkable pituitary gland including pars intermedia, pars distalis and pars nervosa as well and the trigeminal nerve/ganglia (71183). The outer and middle regions of the ear are discernible. The tympanic membrane is intact and the ossicles are unremarkable and include the stapedial annular ligaments (71185-71187). Typical components of the inner ear including bony labyrinth, organ of corti, stria vascularis and scala cavities are discernible. Based on multiple levels, the organ of corti is unremarkable with no discernible loss of inner/outer hair cells and typical tectorial membrane (71186-71187).
The cochlear nerve and spiral ganglion is also demonstrated and based on several levels, there is no reduction in the density of the spiral ganglion cells. Examples of otolith organs can be seen with typical features such as the hair cells and mineral otoliths. The ampulla including the crista ridge with hair cells is discernible (71186-71187).

No lesions of significance
(71183, 71185-71187)

Sternum

For further evaluation of bone marrow:
Section shows hematopoietic tissue islands surrounded by vascular sinuses interspersed within a meshwork of trabecular bone. The bone marrow morphology demonstrated typical myeloid features including conspicuous megakaryoblasts and lymphoid features.
(71426)
No lesions of significance

#634

Macro Observations

Tail suspension test for neurological defects-negative
BCS: 3
Dentition, tongue and oral cavity was unremarkable
Testes: 10x5x4mm, symmetrical
Spleen: 18x6x2mm
Kidneys: 15x10x6mm, symmetrical
Thymus: 7x7x2mm
Lungs inflated
Heart: 15x10x6mm

Brain: 15x10x5mm
Pituitary gland identified, macroscopically normal
Tail length: 80mm (straight)
Head harvested for evaluation of auditory and vestibular structures
Hind leg-Bone marrow smear

Micro Observations

Marrow smear: Examination of the smear showed representative cells from the myeloid and erythroid series. Occasional cells from the lymphoid series. Occasional and unremarkable megakaryoblasts.
(71252)

Peripheral blood smear: Examination of the smear showed red blood cells (majority of cells shown), numerous white blood cells including lymphocytes, segmented neutrophils, monocytes and platelets (clumps). No discernible morphological changes or detectable parasites.
(71253)

Micromorphological changes-
Testes: bilateral, mild focal testicular degeneration (71246), considered an age related finding
Stomach: of the glandular portion, small focus of accumulated submucosal neutrophils (71244), considered an incidental finding
Small intestine: prominent clusters of apoptotic cells (71244, 71231), significance unknown
-Pathology to comment-
Large intestine: numerous intraluminal protozoan parasites (71229), considered an incidental finding
Kidneys: bilateral, mild multifocal perivascular lymphocytic inflammation (71236), considered an age related finding
Salivary glands: of the submandibular gland, mild multifocal perivascular lymphocytic inflammation (71239, 71240), considered an age related finding
Salivary gland: acini cells of the submandibular gland appear less prominent (71239, 71240), significance unknown
-Pathology to comment-
Head: duct adjacent the nasal turbinate shows mild epithelial hyperplasia with intraluminal content admix cellular debris and red blood cells (71249, 71250), considered an incidental finding
Head: of the bottom lip, few hair follicles show neutrophilic inflammation (71248), considered an incidental finding

Testes/Epididymes

Section shows mostly typical convoluted seminiferous tubules at various stages of cycle surrounded by the tunica albuginea. Within the tubules, unremarkable spermatogenic cells including, Sertoli cells, spermatogonia, developing spermatocytes and spermatids. Typical interstitial Leydig cells.
Bilateral, mild focal testicular degeneration, considered an age related finding.
Section also shows unremarkable vas deferens with typical intraluminal sperm.
The architecture of the epididymis is typical, with numerous intraluminal elongated spermatozoa.
(71246)
No lesions of significance

Seminal vesicles

Unremarkable tall columnar epithelium and folded mucosa.
Presence of typical intraluminal eosinophilic secretions.
(71239, 71240)
No lesions of significance

Prostate glands

Sections show unremarkable dorsal lateral/ventral/coagulating glands with typical intraluminal secretions. Sections also include portion of unremarkable vas deferens.
(71239, 71240)
No lesions of significance

Penis/Preputial gland

Sections show typical penile structures including prepuce, glans, corpus cavernosum, os penis, and urethra.
Preputial glands including basal and secretory cells, with mild ductal ectasia (dilated ducts) and flattened epithelium, a common finding in the mouse.
(71238)
No lesions of significance

Urinary Bladder

Unremarkable bladder with typical urothelium and detrusor muscle.
(71239, 71240)
No lesions of significance

Liver/Gall bladder

Section shows typical liver parenchyma including hepatocytes, Kupffer cells, portal triads and central veins.
Unremarkable Gall bladder.
(71237)
No lesions of significance

Stomach

Section shows unremarkable fore and glandular portions of the stomach with limiting ridge.
Section also includes pyloric sphincter and duodenal bulb.
Of the glandular portion, small focus of accumulated submucosal neutrophils, considered an incidental finding.
(71244)
No lesions of significance

Small Intestine (Duodenum, Jejunum & Ileum)/GALT

Sections show typical mucosal villi and submucosal layers. Mucosa shows conspicuous mitotic figures, a typical finding, and prominent clusters of apoptotic cells, significance unknown.
The Peyer's patches display mild follicular hyperplasia with germinal centre formation, indicating a reactive state.
(71244, 71231)

Comments:

Pathology to comment

Cecum/Colon/GALT

Typical mucosal folds and submucosal layers and an occasional lymphoid cluster (Peyer's patch).
Numerous intraluminal protozoan parasites.
(71229)
No lesions of significance

Mesenteric lymph node

Sections show mesenteric lymph node with typical reactive micromorphology including mild follicular hyperplasia, germinal centre formation and mild sinus histiocytosis.
(71240)
No lesions of significance

Spleen

Mild follicular hyperplasia with germinal centre formation and mild coalescence of the white pulp. Mild extramedullary haematopoiesis and hemosiderin laden macrophages identified in the red pulp.
(71239, 71240)
No lesions of significance

Pancreas

Sections show representative exocrine tissue (serous acini) and endocrine tissue (islets of Langerhans).
(71239, 71240)
No lesions of significance

Kidney

Sections show a cortex, medulla, and papilla. There is a uniform distribution of glomeruli and accompanying nephron components and the micromorphology of the tubules is unremarkable. Bilateral, mild multifocal perivascular lymphocytic inflammation, considered an age related finding.
Sections also include renal lymph nodes with typical reactive nodal histology and mild sinus histiocytosis.
(71236)
No lesions of significance

Adrenal glands

Sections show adrenal glands with typical cortex/medulla micromorphology.
(71236)
No lesions of significance

Salivary glands and Regional lymph nodes

Sections show unremarkable submandibular, sublingual and parotid glands.
Mild multifocal perivascular lymphocytic inflammation, considered an incidental finding. Acini cells of the submandibular gland also appear less prominent.
The regional lymph nodes display mild follicular hyperplasia with germinal centre formation, indicating a reactive state.
(71239, 71240)

Comments:

Pathology to comment

Thyroids

Normal lateral lobes of the thyroid gland with typical colloid secreting follicles lined by cuboidal epithelium. Sections also show a small sheet-like mass of polygonal cells, characteristic of the parathyroid gland.
(71223, 71224, 71233)
No lesions of significance

Trachea/Lungs

Sections show typical lung parenchyma/alveoli, bronchioles, and blood vessels.
Trachea with unremarkable mucosal epithelial lining and hyaline cartilage.
Oesophagus with typical features including stratified squamous epithelium.
(71223, 71224, 71233)
No lesions of significance

Thymus

Sections show typical medulla/cortex distribution and micromorphology.
(71223, 71224, 71233)
No lesions of significance

Heart/chambers/vessels/valves

Typical micromorphology observed in cardiac muscle, chambers, valves and greater vessels of the heart.
The cardiac muscle fibres demonstrated typical features including central nuclei, branching fibres and striations.
(71223, 71224, 71233)
No lesions of significance

Skin

Typical dermal appendages and distribution. Unremarkable thin layer of striated muscle (panniculus carnosus).
(71241)
No lesions of significance

Tail

Sections show typical tail components including keratinized squamous epithelium, dense regular connective tissue, tendons, caudal vertebra, bone marrow, intervertebral disc, skeletal muscle, nerves and blood vessels.
(71234, 71245)
No lesions of significance

Eyes/Harderian glands

Section shows eyes with unremarkable retina, cornea, iris, ciliary body, lens, sclera and choroid.
Typical branched tubuloalveolar formation of the Harderian gland.
Section also includes portion of unremarkable optic nerve and extraocular muscles.
(71232)
No lesions of significance

Brain

Sections were prepared from the standard levels of the brain:

Level I forebrain: including cortex, corpus callosum, caudate putamen and lateral ventricles (Bregma 0.02mm)
Level II midbrain: including the hippocampus, thalamus, hypothalamus and lateral and third ventricles (Bregma -1.70mm)
Level III hindbrain: includes the cerebellum, pons and fourth ventricle (Bregma -5.68mm)

Sections of brain stained with Haematoxylin and Eosin, Luxol Fast Blue appear symmetrical with no ventricular dilation observed, unremarkable meninges and typical lamination.
The cerebellum appears symmetrical with typical architecture and Purkinje cells.
There was no obvious neuronal loss and the myelination appears normal.
(71228, 71251)
No lesions of significance

Comments:

Neuropathology to comment

Spinal cord

Representative thoracic and lumbar region of spinal cord, vertebral bone, striated muscle, peripheral nerves, brown adipose tissue, and bone marrow.
(71242)
No lesions of significance

Comments:

Neuropathology to comment

(Hind leg) Long bone/Bone marrow/Synovial joint/Skeletal muscle

Sections show unremarkable long bone, striated muscle, examples of nerve fascicles, fibrocartilage of the meniscus, synovial joint and bone marrow. The skeletal muscle shows consistent fibre size with peripheral nuclei.
(71234, 71245)
No lesions of significance

Head

Multiple levels through the head demonstrate dermal appendages, nasal cavity, oral cavity, teeth and tongue including muscle bundles. Sections also show unremarkable pituitary gland including pars intermedia, pars distalis and pars nervosa as well and the trigeminal nerve/ganglia (71226). The outer and middle regions of the ear are discernible. The tympanic membrane is intact and the ossicles are unremarkable and include the stapedial annular ligaments (71247-71250). Typical components of the inner ear including bony labyrinth, organ of corti, stria vascularis and scala cavities are discernible. Based on multiple levels, the organ of corti is unremarkable with no discernible loss of inner/outer hair cells and typical tectorial membrane (71247-71250).
The cochlear nerve and spiral ganglion is also demonstrated and based on several levels, there is no reduction in the density of the spiral ganglion cells. Examples of otolith organs can be seen with typical features such as the hair cells and mineral otoliths. The ampulla including the crista ridge with hair cells is discernible (71249-71250).

Duct adjacent the nasal turbinate shows mild epithelial hyperplasia with intraluminal content admix cellular debris and red blood cells. Nearby reactive submucosal lymphoid tissue (71249, 71250).
Of the bottom lip, few hair follicles show neutrophilic inflammation, considered an incidental finding (71248).

No lesions of significance
(71226, 71247-71250)

Comments:

Pathology to comment

Sternum

For further evaluation of bone marrow:
Section shows hematopoietic tissue islands surrounded by vascular sinuses interspersed within a meshwork of trabecular bone. The bone marrow morphology demonstrated typical myeloid features including conspicuous megakaryoblasts and lymphoid features.
(71428)
No lesions of significance

#635

Macro Observations

Tail suspension test for neurological defects-negative
BCS: 3
Dentition, tongue and oral cavity was unremarkable
Testes: 8x5x4mm, symmetrical
Spleen: 15x5x3mm
Kidneys: 12x6x6mm, symmetrical
Thymus: 10x10x2mm
Lungs inflated
Heart: 11x7x6mm
Brain: 14x10x5mm
Pituitary gland identified, macroscopically normal

Tail length: 80mm (straight)
Head harvested for evaluation of auditory and vestibular structures
Hind leg-Bone marrow smear

Placcid/ stood still
Excessive visceral fat - abdomen (+)

Micro Observations

Marrow smear: Examination of the smear showed representative cells from the myeloid and erythroid series. Occasional cells from the lymphoid series. Occasional and unremarkable megakaryoblasts.
(71221)

Peripheral blood smear: Examination of the smear showed red blood cells (majority of cells shown), numerous white blood cells including lymphocytes, segmented neutrophils, monocytes and platelets (clumps). No discernible morphological changes or detectable parasites.
(71222)

Micromorphological changes-
Testis: one tubule shows features of testicular degeneration (71210), considered an age related finding
Small intestine: prominent clusters of apoptotic cells (71208, 71195), significance unknown
-Pathology to comment-
Large intestine: numerous intraluminal protozoan parasites (71193), considered an incidental finding
Kidney: a single dilated protein cast within the medulla (71200), considered an incidental finding
Salivary glands: acini cells of the submandibular gland appear less prominent with scattered clusters of mononuclear cellular infiltrates (71203), significance unknown
-Pathology to comment-
Lungs: small focus of intra-alveolar oedema (71197, 71211, 71212), considered an incidental finding
Heart: mild myxomatous valvular changes (thickened leaflets) (71211), considered to be an age related finding
Heart: small focal mineralisation (calcification) of the aortic wall (71211), considered to be an incidental finding
-Pathology to comment-
Skin: several foci of mild epidermal hyperplasia with local neutrophilic inflammation (71205), considered an incidental finding
Head: mild inflammation of one hair follicle in the upper lip (71216), considered an incidental finding

Testes/Epididymes

Section shows mostly typical convoluted seminiferous tubules at various stages of cycle surrounded by the tunica albuginea. Within the tubules, unremarkable spermatogenic cells including, Sertoli cells, spermatogonia, developing spermatocytes and spermatids. Typical interstitial Leydig cells.
Section also shows unremarkable vas deferens with typical intraluminal sperm.
One tubule shows features of testicular degeneration, considered an age related finding.
The architecture of the epididymis is typical, with numerous intraluminal elongated spermatozoa.
(71210)
No lesions of significance

Seminal vesicles

Unremarkable tall columnar epithelium and folded mucosa.
Presence of typical intraluminal eosinophilic secretions.
(71202, 71204)
No lesions of significance

Prostate glands

Sections show unremarkable dorsal lateral/ventral/coagulating glands with typical intraluminal secretions. Sections also include portion of unremarkable vas deferens.
(71202, 71204)
No lesions of significance

Penis/Preputial gland

Sections do not include penile structures.
Typical preputial glands including basal and secretory cells.
(71202, 71204)
No lesions of significance

Urinary Bladder

Unremarkable bladder with typical urothelium and detrusor muscle.
(71202, 71204)
No lesions of significance

Liver/Gall bladder

Section shows typical liver parenchyma including hepatocytes, Kupffer cells, portal triads and central veins.
Unremarkable Gall bladder.
(71201)
No lesions of significance

Stomach

Section shows unremarkable fore and glandular portions of the stomach with limiting ridge.
Section also includes pyloric sphincter and duodenal bulb.
(71208)
No lesions of significance

Small Intestine (Duodenum, Jejunum & Ileum)/GALT

Sections show typical mucosal villi and submucosal layers. Mucosa shows conspicuous mitotic figures, a typical finding, and prominent clusters of apoptotic cells, significance unknown.
The Peyer's patches display mild follicular hyperplasia with germinal centre formation, indicating a reactive state.
(71208, 71195)

Comments:

Pathology to comment

Cecum/Colon/GALT

Typical mucosal folds and submucosal layers and an occasional lymphoid cluster (Peyer's patch).
Numerous intraluminal protozoan parasites.
(71193)
No lesions of significance

Mesenteric lymph node

Sections show mesenteric lymph node with typical reactive micromorphology including mild follicular hyperplasia, germinal centre formation and mild sinus histiocytosis.
(71203)
No lesions of significance

Spleen

Mild follicular hyperplasia with germinal centre formation.
Mild extramedullary haematopoiesis and hemosiderin laden macrophages identified in the red pulp.
(71202, 71204)
No lesions of significance

Pancreas

Sections show representative exocrine tissue (serous acini) and endocrine tissue (islets of Langerhans).
(71202, 71204)
No lesions of significance

Kidney

Sections show a cortex, medulla, and papilla. There is a uniform distribution of glomeruli and accompanying nephron components and the micromorphology of the tubules is unremarkable.
A single dilated protein cast can be seen within the medulla, considered an incidental finding.
(71200)
No lesions of significance

Adrenal glands

Sections show adrenal glands with typical cortex/medulla micromorphology.
(71200)
No lesions of significance

Salivary glands and Regional lymph nodes

Section shows unremarkable sublingual and parotid glands. Acini cells of the submandibular gland appear less prominent with scattered clusters of mononuclear cellular infiltrates.
The regional lymph nodes display mild follicular hyperplasia with germinal centre formation, indicating a reactive state.
(71203)

Comments:

Pathology to comment

Thyroids

Normal lateral lobes of the thyroid gland with typical colloid secreting follicles lined by cuboidal epithelium.
(71197, 71211, 71212)
No lesions of significance

Trachea/Lungs

Sections show typical lung parenchyma/alveoli, bronchioles, blood vessels and parabronchial lymph node.
Small focus of intra-alveolar oedema, considered an incidental finding.
Trachea with unremarkable mucosal epithelial lining and hyaline cartilage.
Oesophagus with typical features including stratified squamous epithelium.
(71197, 71211, 71212)
No lesions of significance

Thymus

Sections show typical medulla/cortex distribution and micromorphology.
(71197, 71211, 71212)
No lesions of significance

Heart/chambers/vessels/valves

Typical expected micromorphology observed in cardiac muscle, chambers, valves and greater vessels of the heart.
The cardiac muscle fibres demonstrated typical features including central nuclei, branching fibres and striations.
Mild myxomatous valvular changes (thickened leaflets), considered an age related finding, and a small focus of mineralisation (calcification) of the aortic wall (71211), considered to be incidental - occurs more commonly in some mouse strains.
(71197, 71211, 71212)
No lesions of significance

Comments:

Pathology to comment

Skin

Typical dermal appendages and distribution. Unremarkable thin layer of striated muscle (panniculus carnosus).
Several foci of mild epidermal hyperplasia with local neutrophilic inflammation, considered an incidental finding.
(71205)
No lesions of significance

Tail

Sections show typical tail components including keratinized squamous epithelium, dense regular connective tissue, tendons, caudal vertebra, bone marrow, intervertebral disc, skeletal muscle, nerves and blood vessels.
(71198, 71209)
No lesions of significance

Eyes/Harderian glands

Section shows eyes with unremarkable retina, cornea, iris, ciliary body, lens, sclera and choroid.
Typical branched tubuloalveolar formation of the Harderian gland.
Section also includes portion of unremarkable optic nerve and extraocular muscles.
(71196)
No lesions of significance

Brain

Sections were prepared from the standard levels of the brain:

Level I forebrain: including cortex, corpus callosum, caudate putamen and lateral ventricles (Bregma 0.62mm)
Level II midbrain: including the hippocampus, thalamus, hypothalamus and lateral and third ventricles (Bregma -1.70mm)
Level III hindbrain: includes the cerebellum, pons and fourth ventricle (Bregma -5.80mm)

Sections of brain stained with Haematoxylin and Eosin, Luxol Fast Blue appear symmetrical with no ventricular dilation observed, unremarkable meninges and typical lamination.
The cerebellum appears symmetrical with typical architecture and Purkinje cells.
There was no obvious neuronal loss and the myelination appears normal.
(71192, 71220)
No lesions of significance

Comments:

Neuropathology to comment

Spinal cord

Representative thoracic and lumbar region of spinal cord, vertebral bone, intervertebral disc, striated muscle, peripheral nerves, brown adipose tissue, and bone marrow.
(71206)
No lesions of significance

Comments:

Neuropathology to comment

(Hind leg) Long bone/Bone marrow/Synovial joint/Skeletal muscle

Sections show unremarkable long bone, striated muscle, examples of nerve fascicles, fibrocartilage of the meniscus, synovial joint and bone marrow. The skeletal muscle shows consistent fibre size with peripheral nuclei.
(71198, 71209)
No lesions of significance

Head

Multiple levels through the head demonstrate dermal appendages, nasal cavity, oral cavity, teeth and tongue including muscle bundles. Sections also show unremarkable pituitary gland including pars intermedia, pars distalis and pars nervosa as well and the trigeminal nerve/ganglia (71214).
The outer and middle regions of the ear are discernible. The tympanic membrane is intact and the ossicles are unremarkable and include the stapedial annular ligaments (71216-71219).
Typical components of the inner ear including bony labyrinth, organ of corti, stria vascularis and scala cavities are discernible. Based on multiple levels, the organ of corti is unremarkable with no discernible loss of inner/outer hair cells and typical tectorial membrane (71216-71219).
The cochlear nerve and spiral ganglion is also demonstrated and based on several levels, there is no reduction in the density of the spiral ganglion cells. Examples of otolith organs can be seen with typical features such as the hair cells and mineral otoliths. The ampulla including the crista ridge with hair cells is discernible (71218-71219).

Mild inflammation of one hair follicle in the upper lip (71216), considered an incidental finding.

No lesions of significance
(71214, 71216-71219)

Sternum

For further evaluation of bone marrow:
Section shows hematopoietic tissue islands surrounded by vascular sinuses interspersed within a meshwork of trabecular bone. The bone marrow morphology demonstrated typical myeloid features including conspicuous megakaryoblasts and lymphoid features.
(71427)
No lesions of significance

#640

Macro Observations

Tail suspension test for neurological defects-negative
BCS: 3
Dentition, tongue and oral cavity was unremarkable
Testes: 7x4x4mm, symmetrical
Spleen: 13x4x3mm
Kidneys: 12x6x5mm, symmetrical
Thymus: 9x8x3mm
Lungs inflated
Heart: 9x6x5mm
Brain: 13x11x5mm
Pituitary gland identified, macroscopically normal
Tail length: 75mm (straight)

Head harvested for evaluation of auditory and vestibular structures
Hind leg-Bone marrow smear

Ileal region felt rigid.

Micro Observations

Marrow smear: Examination of the smear showed representative cells from the myeloid and erythroid series. Occasional cells from the lymphoid series. Occasional and unremarkable megakaryoblasts.
(71314)

Peripheral blood smear: Examination of the smear showed red blood cells (majority of cells shown), occasional white blood cells including lymphocytes, segmented neutrophils, monocytes and platelets (clumps). No discernible morphological changes or detectable parasites.
(71315)

Micromorphological changes-
Skin (penile area): prominent number of melanocytes within the dermis, seemingly accompanied by numerous infiltrates comprised of mast cells and neutrophils (71292, 71294), considered an incidental finding
-Pathology to comment-
Skin (penile area): mild segmental epithelial hyperplasia with local neutrophilic inflammation at times extending in to the deep dermis (71294, 71297), considered an incidental finding
Preputial glands: unilateral, mild scattered mononuclear inflammation accompanied by neutrophils and mast cells (71292, 71294, 71297), considered an incidental finding
Stomach: focal epithelial hyperplasia of the limiting ridge (71298), considered an incidental finding
-Pathology to comment-
Stomach: focal mild submucosal accumulation of neutrophils within the glandular portion (71298), considered an incidental finding
Small intestine: disruption of typical mucosal micromorphology with mild enterocyte hyperplasia, most notably of goblet cells, prominent immune cells within the lamina propria, and cytoplasmic vacuolation of apical cells (71298, 71285, 71307)
-Pathology to comment-
Large intestine: numerous intraluminal protozoan parasites (71307), considered an incidental finding
Mesenteric lymph node: pronounced plasmocytosis and scattered neutrophils through the medullary cords and sinuses (71293)
-Pathology to comment
Kidney: single cystic glomerulus adjacent mild perivascular lymphocytic inflammation, considered an incidental finding (71290)
Lung: mild focal intra-alveolar oedema (71287, 71301, 71302), considered an incidental finding
Thymus: a small pair of cysts can be seen within the medulla (71301), a common finding in the mouse
Heart: mild myxomatous valvular changes (thickened leaflets) (71302), considered an age related finding
Skin: mild focal epithelial hyperplasia with local inflammation of the superficial dermis (71295), considered an incidental finding
Hind leg: small aggregate of lymphoid and red blood cells between the thigh muscle bundles (71288), considered an incidental finding
Head: small cluster of hair follicles adjacent the oral mucosa show neutrophilic inflammation (71311), considered an incidental finding

Testes/Epididymes

Section shows typical convoluted seminiferous tubules at various stages of cycle surrounded by the tunica albuginea. Within the tubules, unremarkable spermatogenic cells including, Sertoli cells, spermatogonia, developing spermatocytes and spermatids. Typical interstitial Leydig cells. Section also shows unremarkable vas deferens with typical intraluminal sperm.
The architecture of the epididymis is typical, with numerous intraluminal elongated spermatozoa.
(71300)
No lesions of significance

Seminal vesicles

Unremarkable tall columnar epithelium and folded mucosa.
Presence of typical intraluminal eosinophilic secretions.
(71292, 71294, 71297)
No lesions of significance

Prostate glands

Sections show unremarkable dorsal lateral/ventral/coagulating glands with typical intraluminal secretions. Sections also include portion of unremarkable vas deferens.
(71292, 71294, 71297)
No lesions of significance

Penis/Preputial gland

Sections show typical penile structures including prepuce, glans, corpus cavernosum and urethra.
Section of the skin shows prominent number of melanocytes within the dermis, seemingly accompanied by numerous infiltrates comprised of mast cells and neutrophils (71292, 71294), likely to be incidental.
Elsewhere, skin also shows mild segmental epithelial hyperplasia with local neutrophilic inflammation, at times extending in to the deep dermis (71294, 71297), considered an incidental finding.
Mostly typical preputial glands including basal and secretory cells. Unilateral, mild scattered mononuclear inflammation with accompanying neutrophils and mast cells, considered an incidental finding.
(71292, 71294, 71297)

Comments:

Pathology to comment

Urinary Bladder

Unremarkable bladder with typical urothelium and detrusor muscle.
(71292, 71294, 71297)
No lesions of significance

Liver/Gall bladder

Section shows typical liver parenchyma including hepatocytes, Kupffer cells, portal triads and central veins.
Unremarkable Gall bladder.
(71291)
No lesions of significance

Stomach

Section shows unremarkable fore and glandular portions of the stomach. Mild focal epithelial hyperplasia of the limiting ridge, and mild focal submucosal accumulation of neutrophils within the glandular portion, considered to be incidental findings.
Section also includes pyloric sphincter and duodenal bulb.
(71298)

Comments:

Pathology to comment

Small Intestine (Duodenum, Jejunum & Ileum)/GALT

Sections show representative mucosal villi and submucosal layers.
Disruption of typical mucosal micromorphology with mild enterocyte hyperplasia, most notably of goblet cells, prominent immune cells within the lamina propria, and cytoplasmic vacuolation of apical cells. Ileum appears to be most affected (71285, 71307).
The Peyer’s patches display mild follicular hyperplasia with germinal centre formation, indicating

a reactive state.
(71298, 71285, 71307)

Comments:

Pathology to comment

Cecum/Colon/GALT

Typical mucosal folds and submucosal layers and an occasional lymphoid cluster (Peyer's patch). Intraluminal space of the caecum and proximal colon occupied by numerous protozoan parasites.
(71307)
No lesions of significance

Mesenteric lymph node

Sections show mesenteric lymph node with typical reactive micromorphology including mild follicular hyperplasia, germinal centre formation and mild sinus histiocytosis. Medullary cords and sinuses occupied by pronounced plasmocytosis and scattered neutrophils.
(71293)

Comments:

Pathology to comment

Spleen

Mild follicular hyperplasia with germinal centre formation of the white pulp, and mild extramedullary haematopoiesis identified in the red pulp.
(71292, 71294, 71297)
No lesions of significance

Pancreas

Sections show representative exocrine tissue (serous acini) and endocrine tissue (islets of Langerhans).
(71292, 71294, 71297)
No lesions of significance

Kidney

Sections show a cortex, medulla, and papilla. There is a uniform distribution of glomeruli and accompanying nephron components and the micromorphology of the tubules is unremarkable.
The cuboidal parietal cells lining the Bowman's capsule of the male kidneys are conspicuous.
Single cystic glomerulus adjacent mild perivascular lymphocytic inflammation, considered an incidental finding.
Sections also include renal lymph nodes with typical reactive nodal histology and mild sinus histiocytosis.
(71290)
No lesions of significance

Adrenal glands

Sections show adrenal glands with typical cortex/medulla micromorphology.
(71290)
No lesions of significance

Salivary glands and Regional lymph nodes

Sections show unremarkable submandibular, sublingual and parotid glands.
The regional lymph nodes display mild follicular hyperplasia with germinal centre formation, indicating a reactive state.
(71293)
No lesions of significance

Thyroids

Normal lateral lobes of the thyroid gland with typical colloid secreting follicles lined by cuboidal epithelium.
(71287, 71301, 71302)
No lesions of significance

Trachea/Lungs

Sections show mostlytypical lung parenchyma/alveoli, bronchioles, blood vessels and parabronchial lymph node.
Mild focal intra-alveolar oedema, considered to be an incidental finding.
Trachea with unremarkable mucosal epithelial lining and hyaline cartilage.
Oesophagus with typical features including stratified squamous epithelium.
(71287, 71301, 71302)
No lesions of significance

Thymus

Sections show typical medulla/cortex distribution and micromorphology.
A pair of small medullary cysts can be seen (71301), a common finding in the mouse.
(71287, 71301, 71302)
No lesions of significance

Heart/chambers/vessels/valves

Typical expected micromorphology observed in cardiac muscle, chambers, valves and greater vessels of the heart.
The cardiac muscle fibres demonstrated typical features including central nuclei, branching fibres and striations.
Mild myxomatous valvular changes (thickened leaflets) (71302), considered an age related finding.
(71287, 71301, 71302)
No lesions of significance

Skin

Typical dermal appendages and distribution. Unremarkable thin layer of striated muscle (panniculus carnosus).
Mild focal epithelial hyperplasia with local inflammation of the superficial dermis, considered an incidental finding.
(71295)
No lesions of significance

Tail

Sections show typical tail components including keratinized squamous epithelium, dense regular connective tissue, tendons, caudal vertebra, bone marrow, intervertebral disc, skeletal muscle, nerves and blood vessels.
(71288, 71299)
No lesions of significance

Eyes/Harderian glands

Section shows eyes with unremarkable retina, cornea, iris, ciliary body, lens, sclera and choroid.
Typical branched tubuloalveolar formation of the Harderian gland.
Section also includes portion of extraocular muscles.
(71286)
No lesions of significance

Brain

Sections were prepared from the standard levels of the brain:

Level I forebrain: including cortex, corpus callosum, caudate putamen and lateral ventricles (Bregma -0.10mm)
Level II midbrain: including the hippocampus, thalamus, hypothalamus and lateral and third ventricles (Bregma -1.58mm)
Level III hindbrain: includes the cerebellum, pons and fourth ventricle (Bregma N/A)

Sections of brain stained with Haematoxylin and Eosin, Luxol Fast Blue appear symmetrical with no ventricular dilation observed, unremarkable meninges and typical lamination.
The cerebellum appears symmetrical with typical architecture and Purkinje cells.
There was no obvious neuronal loss and the myelination appears normal.
(71306, 71313)
No lesions of significance

Comments:

Neuropathology to comment

Spinal cord

Representative thoracic and lumbar region of spinal cord, vertebral bone, striated muscle, peripheral nerves, brown adipose tissue, and bone marrow.
(71296)
No lesions of significance

Comments:

Neuropathology to comment

(Hind leg) Long bone/Bone marrow/Synovial joint/Skeletal muscle

Sections show unremarkable long bone, striated muscle, examples of nerve fascicles, fibrocartilage of the meniscus, synovial joint and bone marrow. The skeletal muscle shows consistent fibre size with peripheral nuclei.
Small aggregate admix lymphoid and red blood cells located between the thigh muscle bundles (71288), considered an incidental finding.
(71288, 71299)
No lesions of significance

Head

Multiple levels through the head demonstrate dermal appendages, nasal cavity, oral cavity, teeth and tongue including muscle bundles. Sections also show unremarkable pituitary gland including pars intermedia, pars distalis and pars nervosa as well and the trigeminal nerve/ganglia (71303).
The outer and middle regions of the ear are discernible. The tympanic membrane is intact and the ossicles are unremarkable and include the stapedial annular ligaments (71309-71311).
Typical components of the inner ear including bony labyrinth, organ of corti, stria vascularis and scala cavities are discernible. Based on multiple levels, the organ of corti is unremarkable with no discernible loss of inner/outer hair cells and typical tectorial membrane (71309-71311).
The cochlear nerve and spiral ganglion is also demonstrated and based on several levels, there is no reduction in the density of the spiral ganglion cells. Examples of otolith organs can be seen with typical features such as the hair cells and mineral otoliths. The ampulla including the crista ridge with hair cells is discernible (71310-71311).

Small cluster of hair follicles adjacent the oral mucosa show neutrophilic inflammation (71311), considered an incidental finding.

No lesions of significance
(71303, 71309-71311)

Sternum

For further evaluation of bone marrow:
Section shows hematopoietic tissue islands surrounded by vascular sinuses interspersed within a meshwork of trabecular bone. The bone marrow morphology demonstrated typical myeloid features including conspicuous megakaryoblasts and lymphoid features.
(71430)
No lesions of significance

#641

Macro Observations

Tail suspension test for neurological defects-negative
BCS: 3
Dentition, tongue and oral cavity was unremarkable
Testes: 10x6x5mm, symmetrical
Spleen: 14x8x2mm
Kidneys: 15x10x6mm, symmetrical
Thymus: 7x7x2mm
Lungs inflated
Heart: 14x11x7mm
Brain: 15x10x5mm
Pituitary gland identified, macroscopically normal
Tail length: 80mm (straight)
Head harvested for evaluation of auditory and vestibular structures
Hind leg-Bone marrow smear

Micro Observations

Marrow smear: Examination of the smear showed representative cells from the myeloid and erythroid series. Occasional cells from the lymphoid series. Occasional and unremarkable megakaryoblasts.
(71283)

Peripheral blood smear: Examination of the smear showed red blood cells (majority of cells shown), occasional white blood cells including lymphocytes, segmented neutrophils, monocytes and platelets (clumps). No discernible morphological changes or detectable parasites.
(71284)

Micromorphological changes-
Preputial glands: scattered perivascular neutrophils (71264), considered an incidental finding
Small intestine: disruption of typical mucosal micromorphology with mild enterocyte hyperplasia, most notably of goblet cells, prominent immune cells within the lamina propria, and cytoplasmic vacuolation of apical cells (71257, 71255)
-Pathology to comment-
Large intestine: numerous intraluminal protozoan parasites (71255), considered an incidental finding
Mesenteric lymph node: medullary cords and sinuses occupied by pronounced plasmocytosis and scattered neutrophils (71255, 71265, 71266, 71269)
-Pathology to comment-
Thymus: small cyst within the cortex (71273), considered an incidental finding
Heart: mild myxomatous valvular changes (thickened leaflets) (71273, 71274), considered an age related finding
Head: neutrophilic inflammation of a single hair follicle adjacent the oral cavity (71280), considered an incidental finding
Sternum: disruption of typical intersternebral joint micromorphology (71429), likely to be artefactual
-Pathology to comment-

Testes/Epididymes

Section shows typical convoluted seminiferous tubules at various stages of cycle surrounded by the tunica albuginea. Within the tubules, unremarkable spermatogenic cells including, Sertoli cells, spermatogonia, developing spermatocytes and spermatids. Typical interstitial Leydig cells.
Section also shows unremarkable vas deferens with typical intraluminal sperm.
The architecture of the epididymis is typical, with numerous intraluminal elongated spermatozoa.
(71272)
No lesions of significance

Seminal vesicles

Unremarkable tall columnar epithelium and folded mucosa.
Presence of typical intraluminal eosinophilic secretions.
(71265, 71266, 71269)
No lesions of significance

Prostate glands

Sections show unremarkable dorsal lateral/ventral/coagulating glands with typical intraluminal secretions. Sections also include portion of unremarkable vas deferens.
(71265, 71266, 71269)
No lesions of significance

Penis/Preputial gland

Sections show typical penile structures including prepuce, glans, corpus cavernosum and urethra.
Typical preputial glands including basal and secretory cells. Mild ductal ectasia (dilated ducts), a common finding in the mouse. Scattered perivascular neutrophils, considered an incidental finding.
(71264)
No lesions of significance

Urinary Bladder

Unremarkable bladder with typical urothelium and detrusor muscle.
(71266, 71269)
No lesions of significance

Liver/Gall bladder

Section shows typical liver parenchyma including hepatocytes, Kupffer cells, portal triads and central veins.
Unremarkable Gall bladder.
(71263)
No lesions of significance

Stomach

Section shows unremarkable fore and glandular portions of the stomach with limiting ridge.
Section also includes pyloric sphincter and duodenal bulb.
(71270)
No lesions of significance

Small Intestine (Duodenum, Jejunum & Ileum)/GALT

Sections show representative mucosal villi and submucosal layers.
Disruption of typical mucosal micromorphology with mild enterocyte hyperplasia, most notably of goblet cells, prominent immune cells within the lamina propria, and cytoplasmic vacuolation of apical cells. Ileum appears to be most affected (71257, 71255).
The Peyer’s patches display mild follicular hyperplasia with germinal centre formation, indicating a reactive state.
(71270, 71257, 71255)

Comments:

Pathology to comment

Cecum/Colon/GALT

Typical mucosal folds and submucosal layers and an occasional lymphoid cluster (Peyer's patch). Numerous intraluminal protozoan parasites.
(71255)
No lesions of significance

Mesenteric lymph node

Sections show mesenteric lymph node with reactive micromorphology including mild follicular hyperplasia, germinal centre formation and mild sinus histiocytosis. Medullary cords and sinuses occupied by pronounced plasmocytosis and scattered neutrophils.
(71255, 71265, 71266, 71269)

Comments:

-Pathology to comment-

Spleen

Unremarkable follicular pattern identified with typical red and white pulp micromorphology.
Mild extramedullary haematopoiesis identified in the red pulp of the spleen, a common finding in the mouse.
(71265, 71266, 71269)
No lesions of significance

Pancreas

Sections show representative exocrine tissue (serous acini) and endocrine tissue (islets of Langerhans).
(71265, 71266, 71269)
No lesions of significance

Kidney

Sections show a cortex, medulla, and papilla. There is a uniform distribution of glomeruli and accompanying nephron components and the micromorphology of the tubules is unremarkable.
The cuboidal parietal cells lining the Bowman's capsule of the male kidneys are conspicuous.
Sections also include renal lymph nodes with typical reactive nodal histology and mild sinus histiocytosis.
(71262)
No lesions of significance

Adrenal glands

Sections show an adrenal gland with typical cortex/medulla micromorphology.
(71262)
No lesions of significance

Salivary glands and Regional lymph nodes

Sections show unremarkable submandibular, sublingual and parotid glands.
The regional lymph nodes display mild follicular hyperplasia with germinal centre formation, indicating a reactive state.
(71265, 71266, 71269)
No lesions of significance

Thyroids

Normal lateral lobes of the thyroid gland with typical colloid secreting follicles lined by cuboidal epithelium. Sections also show a small sheet-like mass of polygonal cells, characteristic of the parathyroid gland.
(71273, 71274)
No lesions of significance

Trachea/Lungs

Sections show typical lung parenchyma/alveoli, bronchioles, and blood vessels.
Trachea with unremarkable mucosal epithelial lining and hyaline cartilage.
Oesophagus with typical features including stratified squamous epithelium.
(71259, 71273, 71274)
No lesions of significance

Thymus

Sections show typical medulla/cortex distribution and micromorphology.
A small cyst can be seen within the cortex (71273), considered an incidental finding.
(71259, 71273, 71274)
No lesions of significance

Heart/chambers/vessels/valves

Typical expected micromorphology observed in cardiac muscle, chambers, valves and greater vessels of the heart.
The cardiac muscle fibres demonstrated typical features including central nuclei, branching fibres and striations.
Mild myxomatous valvular changes (thickened leaflets) (71273, 71274), considered an age related finding.
(71259, 71273, 71274)
No lesions of significance

Skin

Typical dermal appendages and distribution. Unremarkable thin layer of striated muscle (panniculus carnosus).
(71267)
No lesions of significance

Tail

Sections show typical tail components including keratinized squamous epithelium, dense regular connective tissue, tendons, caudal vertebra, bone marrow, intervertebral disc, skeletal muscle, nerves and blood vessels.
(71260, 71271)
No lesions of significance

Eyes/Harderian glands

Section shows eyes with unremarkable retina, cornea, iris, ciliary body, lens, sclera and choroid.
Typical branched tubuloalveolar formation of the Harderian gland.
Section also includes portion of unremarkable optic nerve and extraocular muscles.
(71258)
No lesions of significance

Brain

Sections were prepared from the standard levels of the brain:

Level I forebrain: including cortex, corpus callosum, caudate putamen and lateral ventricles (Bregma -0.46mm)
Level II midbrain: including the hippocampus, thalamus, hypothalamus and lateral and third ventricles (Bregma -2.06mm)

Level III hindbrain: includes the cerebellum, pons and fourth ventricle (Bregma -5.52mm)

Sections of brain stained with Haematoxylin and Eosin, Luxol Fast Blue appear symmetrical with no ventricular dilation observed, unremarkable meninges and typical lamination.
The cerebellum appears symmetrical with typical architecture and Purkinje cells.
There was no obvious neuronal loss and the myelination appears normal.
(71254, 71282)
No lesions of significance

Comments:

Neuropathology to comment

Spinal cord

Representative thoracic and lumbar region of spinal cord, vertebral bone, intervertebral disc, striated muscle, peripheral nerves, brown adipose tissue, and bone marrow.
(71268)
No lesions of significance

Comments:

Neuropathology to comment

(Hind leg) Long bone/Bone marrow/Synovial joint/Skeletal muscle

Sections show unremarkable long bone, striated muscle, examples of nerve fascicles, fibrocartilage of the meniscus, synovial joint and bone marrow. The skeletal muscle shows consistent fibre size with peripheral nuclei.
(71260, 71271)
No lesions of significance

Head

Multiple levels through the head demonstrate dermal appendages, nasal cavity, oral cavity, teeth and tongue including muscle bundles. Sections also show unremarkable pituitary gland including pars intermedia, pars distalis and pars nervosa as well and the trigeminal nerve/ganglia (71276).
The outer and middle regions of the ear are discernible. The tympanic membrane is intact and the ossicles are unremarkable and include the stapedial annular ligaments (71278-71280).
Typical components of the inner ear including bony labyrinth, organ of corti, stria vascularis and scala cavities are discernible. Based on multiple levels, the organ of corti is unremarkable with no discernible loss of inner/outer hair cells and typical tectorial membrane (71278-71280).
The cochlear nerve and spiral ganglion is also demonstrated and based on several levels, there is no reduction in the density of the spiral ganglion cells. Examples of otolith organs can be seen with typical features such as the hair cells and mineral otoliths. The ampulla including the crista ridge with hair cells is discernible (71279-71280).

Neutrophilic inflammation of a single hair follicle adjacent the oral cavity (71280), considered an incidental finding.

No lesions of significance
(71276, 71278-71280)

Sternum

For further evaluation of bone marrow:
Section shows hematopoietic tissue islands surrounded by vascular sinuses interspersed within a meshwork of trabecular bone. The bone marrow morphology demonstrated typical myeloid features including conspicuous megakaryoblasts and lymphoid features.
Multifocal disruption of typical intersternebral joint micromorphology, likely to be artefactual.
(71429)
No lesions of significance

Comments:

Pathology to comment

#642

Macro Observations

Tail suspension test for neurological defects-negative
BCS: 3
Dentition, tongue and oral cavity was unremarkable
Testes: 8x4x4mm, symmetrical
Spleen: 14x4x3mm
Kidneys: 12x6x5mm, symmetrical
Thymus: 10x8x3mm
Lungs inflated
Heart: 10x6x5mm
Brain: 15x10x5mm
Pituitary gland identified, macroscopically normal
Tail length: not measured (straight)
Head harvested for evaluation of auditory and vestibular structures
Hind leg-Bone marrow smear

Excessive visceral fat - abdomen (+)
Ileal region felt rigid.

Micro Observations

Marrow smear: Examination of the smear showed representative cells from the myeloid and erythroid series. Occasional cells from the lymphoid series. Occasional and unremarkable megakaryoblasts.
(71345)

Peripheral blood smear: Examination of the smear showed red blood cells (majority of cells shown), occasional white blood cells including lymphocytes, segmented neutrophils, monocytes and platelets (clumps). No discernible morphological changes or detectable parasites.
(71346)

Micromorphological changes-
Testes: bilateral, mild multifocal testicular degeneration (71334), considered an age related finding
Preputial glands: bilateral, mild scattered inflammation comprised of mononuclear cells and neutrophils (71326), considered an incidental finding
Stomach: limiting ridge shows mild epithelial hyperplasia (71332), considered an incidental finding
-Pathology to comment-
Stomach: few foci of mild submucosal neutrophilic inflammation within the glandular portion (71332), considered an incidental finding
Small intestine: primarily within the ileum- disruption of typical mucosal micromorphology with mild enterocyte hyperplasia, most notably of goblet cells, and prominent immune cells within the lamina propria (71323)
-Pathology to comment-
Salivary glands: mild multifocal perivascular lymphocytic inflammation within the submandibular gland (71327), considered an age related finding
Thymus: a small cyst can be seen within the medulla (71336), a common finding in the mouse
Heart: mild myxomatous valvular changes (thickened leaflets) (71321, 71335, 71336), considered an age related finding

Testes/Epididymes

Section shows mostly typical convoluted seminiferous tubules at various stages of cycle surrounded by the tunica albuginea. Within these these tubules are unremarkable spermatogenic cells including, Sertoli cells, spermatogonia, developing spermatocytes and spermatids. Typical interstitial Leydig cells.
Bilateral, mild multifocal testicular degeneration, considered to be an age related finding.

Section also shows unremarkable vas deferens with typical intraluminal sperm.
The architecture of the epididymis is typical, with discernible intraluminal elongated spermatozoa.
(71334)
No lesions of significance

Seminal vesicles

Unremarkable tall columnar epithelium and folded mucosa.
Presence of typical intraluminal eosinophilic secretions.
(71328, 71331)
No lesions of significance

Prostate glands

Sections show unremarkable dorsal lateral/ventral/coagulating glands with typical intraluminal secretions. Sections also include portion of unremarkable vas deferens.
(71328, 71331)
No lesions of significance

Penis/Preputial gland

Sections show typical penile structures including prepuce, glans, corpus cavernosum and urethra.
Representaive sections of preputial glands including basal and secretory cells. Bilateral, mild scattered inflammation comprised of mononuclear cells and neutrophils, considered an incidental finding.
(71326)
No lesions of significance

Urinary Bladder

Unremarkable bladder with typical urothelium and detrusor muscle.
(71328, 71331)
No lesions of significance

Liver/Gall bladder

Section shows typical liver parenchyma including hepatocytes, Kupffer cells, portal triads and central veins.
Unremarkable Gall bladder.
(71325)
No lesions of significance

Stomach

Section shows unremarkable fore and glandular portions of the stomach. Limiting ridge shows mild epithelial hyperplasia.
Few foci of mild submucosal neutrophilic inflammation within the glandular portion, considered an incidental finding.
Section also includes pyloric sphincter and duodenal bulb.
(71332)

Comments:

Pathology to comment

Small Intestine (Duodenum, Jejunum & Ileum)/GALT

Sections show representative mucosal villi and submucosal layers. Disruption of typical mucosal micromorphology with mild enterocyte hyperplasia, most notably of goblet cells, and prominent immune cells within the lamina propria. Ileum appears to be most affected (71323).
The Peyer’s patches display mild follicular hyperplasia with germinal centre formation, indicating a reactive state.
(71332, 71323)

Comments:

Pathology to comment

Cecum/Colon/GALT

Typical mucosal folds and submucosal layers and an occasional lymphoid cluster (Peyer's patch).
(71317)
No lesions of significance

Mesenteric lymph node

Sections show mesenteric lymph node with typical reactive micromorphology including mild follicular hyperplasia, germinal centre formation and mild sinus histiocytosis.
(71327)
No lesions of significance

Spleen

White pulp shows mild follicular hyperplasia with germinal centre formation and marked lymphocytic apoptosis, and mild coalescence.
Mild extramedullary haematopoiesis identified in the red pulp.
(71328, 71331)
No lesions of significance

Pancreas

Sections show representative exocrine tissue (serous acini) and endocrine tissue (islets of Langerhans).
(71328, 71331)
No lesions of significance

Kidney

Sections show a cortex, medulla, and papilla. There is a uniform distribution of glomeruli and accompanying nephron components and the micromorphology of the tubules is unremarkable.
The cuboidal parietal cells lining the Bowman’s capsule of the male kidneys are conspicuous.
(71324)
No lesions of significance

Adrenal glands

Sections show adrenal glands with typical cortex/medulla micromorphology.
(71324)
No lesions of significance

Salivary glands and Regional lymph nodes

Sections show unremarkable sublingual and parotid glands.
Mild multifocal perivascular lymphocytic inflammation within the submandibular gland, considered an age related finding.
The regional lymph nodes display mild follicular hyperplasia with germinal centre formation, indicating a reactive state.
(71327)
No lesions of significance

Thyroids

Normal lateral lobes of the thyroid gland with typical colloid secreting follicles lined by cuboidal epithelium.
(71321, 71335)
No lesions of significance

Trachea/Lungs

Sections show typical lung parenchyma/alveoli, bronchioles, and blood vessels.
Trachea with unremarkable mucosal epithelial lining and hyaline cartilage.
Oesophagus with typical features including stratified squamous epithelium.
(71321, 71335, 71336)
No lesions of significance

Thymus

Sections show typical medulla/cortex distribution and micromorphology.
A small cyst can be seen within the medulla (71336), considered an incidental finding.
(71321, 71335, 71336)
No lesions of significance

Heart/chambers/vessels/valves

Typical expected micromorphology observed in cardiac muscle, chambers, valves and greater vessels of the heart.
The cardiac muscle fibres demonstrated typical features including central nuclei, branching fibres and striations.
Mild myxomatous valvular changes (thickened leaflets), considered an age related finding.
(71321, 71335, 71336)
No lesions of significance

Skin

Typical dermal appendages and distribution. Unremarkable thin layer of striated muscle (panniculus carnosus).
(71329)
No lesions of significance

Tail

Sections show typical tail components including keratinized squamous epithelium, dense regular connective tissue, tendons, caudal vertebra, bone marrow, intervertebral disc, skeletal muscle, nerves and blood vessels.
(71322, 71333)
No lesions of significance

Eyes/Harderian glands

Section shows eyes with unremarkable retina, cornea, iris, ciliary body, lens, sclera and choroid.
Typical branched tubuloalveolar formation of the Harderian gland.
Section also includes portion of unremarkable optic nerve and extraocular muscles.
(71320)
No lesions of significance

Brain

Sections were prepared from the standard levels of the brain:

Level I forebrain: including cortex, corpus callosum, caudate putamen and lateral ventricles (Bregma -0.34mm)
Level II midbrain: including the hippocampus, thalamus, hypothalamus and lateral and third ventricles (Bregma -1.94mm)
Level III hindbrain: includes the cerebellum, pons and fourth ventricle (Bregma -5.80mm)

Sections of brain stained with Haematoxylin and Eosin, Luxol Fast Blue appear symmetrical with no ventricular dilation observed, unremarkable meninges and typical lamination.
The cerebellum appears symmetrical with typical architecture and Purkinje cells.
There was no obvious neuronal loss and the myelination appears normal.
(71316, 71344)
No lesions of significance

Comments:

Neuropathology to comment

Spinal cord

Representative thoracic and lumbar region of spinal cord, vertebral bone, striated muscle, peripheral nerves, brown adipose tissue, and bone marrow.
(71330)
No lesions of significance

Comments:

Neuropathology to comment

(Hind leg) Long bone/Bone marrow/Synovial joint/Skeletal muscle

Sections show unremarkable long bone, striated muscle, examples of nerve fascicles, synovial joint and bone marrow. The skeletal muscle shows consistent fibre size with peripheral nuclei.
(71322, 71333)
No lesions of significance

Head

Multiple levels through the head demonstrate dermal appendages, nasal cavity, oral cavity, teeth and tongue including muscle bundles. Sections also show unremarkable pituitary gland including pars intermedia, pars distalis and pars nervosa as well and the trigeminal nerve/ganglia (71337).
The outer and middle regions of the ear are discernible. The tympanic membrane is intact and the ossicles are unremarkable and include the stapedial annular ligaments (71340-71743).
Typical components of the inner ear including bony labyrinth, organ of corti, stria vascularis and scala cavities are discernible. Based on multiple levels, the organ of corti is unremarkable with no discernible loss of inner/outer hair cells and typical tectorial membrane (71340-71743).
The cochlear nerve and spiral ganglion is also demonstrated and based on several levels, there is no reduction in the density of the spiral ganglion cells. Examples of otolith organs can be seen with typical features such as the hair cells and mineral otoliths. The ampulla including the crista ridge with hair cells is discernible (71341-71743).

No lesions of significance
(71337, 71340-71743)

Sternum

For further evaluation of bone marrow:
Section shows hematopoietic tissue islands surrounded by vascular sinuses interspersed within a meshwork of trabecular bone. The bone marrow morphology demonstrated typical myeloid features including conspicuous megakaryoblasts and lymphoid features.
(71431)
No lesions of significance

Comment / Plan

Case APN18/039SVI will be referred for comment.

30th January, 2019

Supplementary Pathology Report

#629

Stomach (71177): limiting ridge with orthokeratotic hyperkeratosis. Focal mineralisation also in ridge.

Small intestine (71164, 71162): numerous apoptotic bodies, especially in basal regions of crypts, with some villous atrophy. Resembles a radiomimetic effect such as that associated with irradiation or anti-cancer chemotherapeutic drugs.

Salivary gland (71173): in some areas, glandular epithelium seems atrophic with higher nucleus to cytoplasmic ratio. Ducts appear unaffected.

#634

Small intestine (71244, 71231): numerous apoptotic bodies, especially in basal regions of crypts, with some villous atrophy. Resembles a radiomimetic effect such as that associated with irradiation or anti-cancer chemotherapeutic drugs.

Salivary gland (00071240): in some areas, glandular epithelium seems atrophic with higher nucleus to cytoplasmic ratio with mild lymphocytic infiltrate.

Head (71250): in skin, adnexal gland duct is markedly dilated with proteinaceous material and lining epithelium is either hyperplastic or attenuated with robust paraductal lymphocytic infiltrate.

#635

Small intestine (71208, 71195): numerous apoptotic bodies, especially in basal regions of crypts, with some villous atrophy. Resembles a radiomimetic effect such as that associated with irradiation or anti-cancer chemotherapeutic drugs. Some regenerative activity present with more enterocytes in mitosis.

Salivary gland (71203): atrophy of acinar elements.

Heart (71211): focal bone mass in aorta at origin near heart – appears to be mature bone with osteocytes in lacunae and well-mineralised matrix (? osseous metaplasia).

#640

Skin (71292): increased numbers of heavily pigmented dermal melanocytes and mastocytosis.

Skin (71294): as above - with attendant eosinophilia in some sites.

Prepucial gland (71292, 71294): focal mast cell, polymorphonuclear, and plasma cell infiltrate of stroma.

Mesenteric lymph node (71297): sinus histiocytosis with increased neutrophil and plasma cell numbers.

Stomach (71298): limiting ridge shows orthokeratotic hyperkeratosis. It appears elongated, but likely plane of section artefact.

Small intestine (71285, 71307): mild acute enteritis with increased numbers of neutrophils and plasma cells in lamina propria and enterocyte hyperplasia.

#641

Small intestine (71255, 71257): mild acute enteritis with increased numbers of neutrophils and plasma cells in lamina propria and enterocyte hyperplasia.

Mesenteric lymph node (71255): sinus histiocytosis with increased neutrophil and plasma cell numbers.

Mesenteric lymph node (71266): similar features as above – with more abundant plasma cells, some showing increased immunoglobulin production in the form of cytoplasmic Russell bodies.

Sternum (71429): appears to be loss of matrix staining in articular cartilage with some loss of ground substance (necrotic), possibly due to physical trauma.

#642

Stomach (71332): at glandular/non-glandular junction, orthokeratotic hyperkeratosis of keratinocytes.

Small intestine (71323): mild acute enteritis with increased numbers of neutrophils and plasma cells in lamina propria and enterocyte hyperplasia.

Summary

In the small intestine of three animals (#629, #634 and #635) there are numerous apoptotic bodies, especially in basal regions of the crypts, with some villous atrophy. These changes resemble a radiomimetic effect such as that associated with irradiation or anti-cancer chemotherapeutic drugs. Of the same animals, the salivary glands seem to have varying degrees of acinar atrophy.

The small intestine of the remaining three animals (#640, #641 and #642) show mild acute enteritis with increased numbers of neutrophils and plasma cells in lamina propria and enterocyte hyperplasia.

For more comments, please see above report.

5th February, 2019

Supplementary Neuropathology Report

#629

Representative H&E stained sections of brain and spinal cord were examined.
No lesions of significance.

#634

Representative H&E stained sections of brain and spinal cord were examined.
No lesions of significance.

#635

Representative H&E stained sections of brain and spinal cord were examined.
No lesions of significance.

#640

Representative H&E stained sections of brain and spinal cord were examined.
No lesions of significance.

#641

Representative H&E stained sections of brain and spinal cord were examined.
No lesions of significance.

#642

Representative H&E stained sections of brain and spinal cord were examined.
No lesions of significance.

Summary

Sections of brain and spinal cord show no significant neuropathology.

5th February, 2019

The Australian Phenomics Network advises all research groups that images or results obtained through the services offered by the APN are to be acknowledged in resultant publications. Example acknowledgement: "This study utilised the Australian Phenomics Network Histopathology and Organ Pathology Service, University of Melbourne."

APN18-039SVI(C. Walkley)
Macro Images

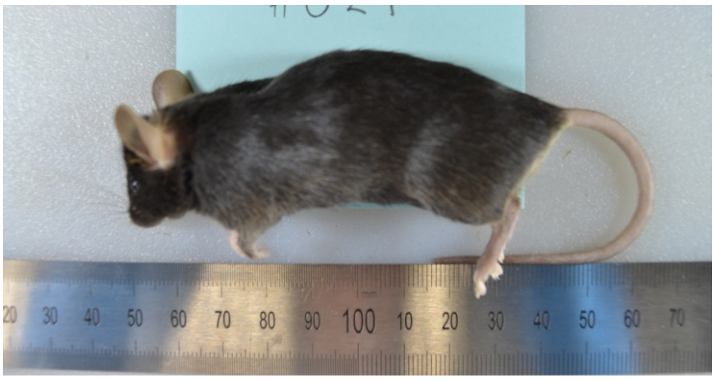

#629: Sideview

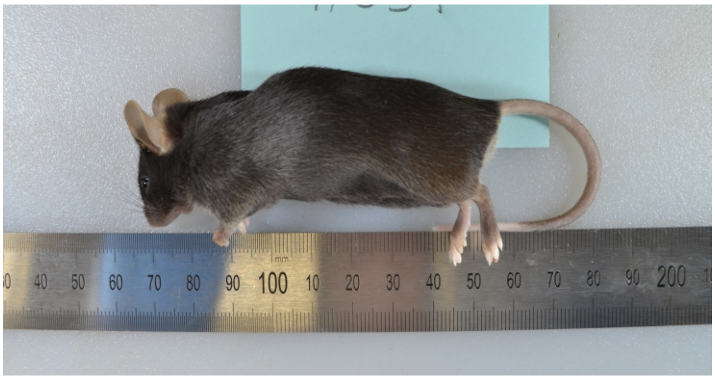

#634: Sideview

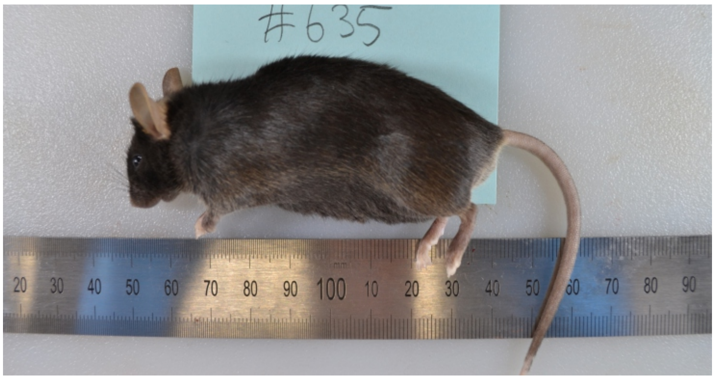

#635: Sideview

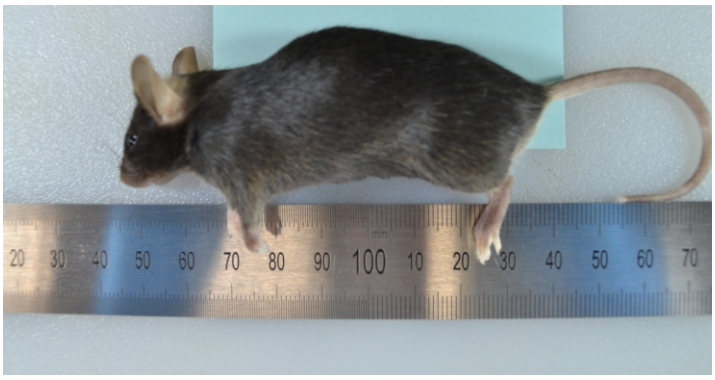

#640: Sideview

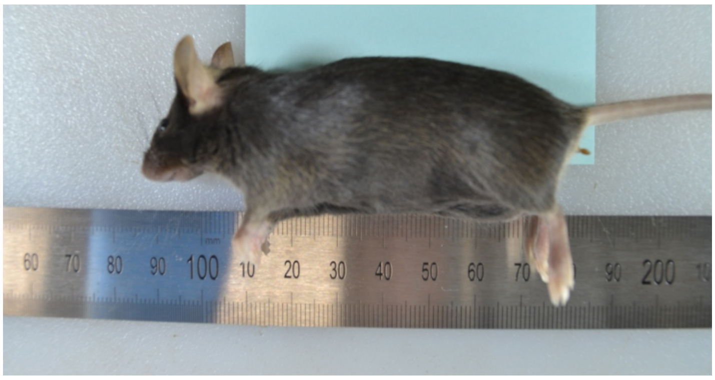

#641: Sideview

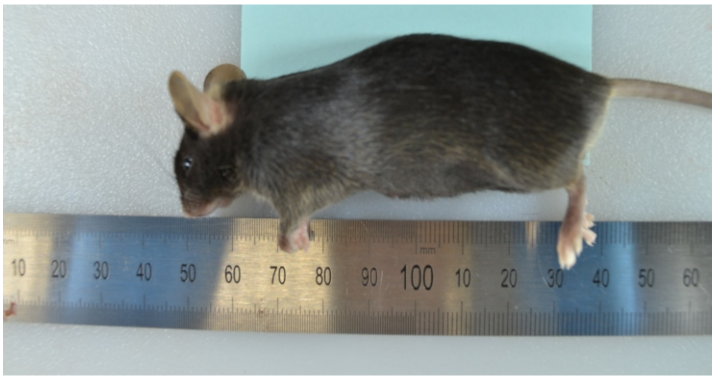

#642: Sideview

APN18/039SVI
Micro Images

Carl Walkley

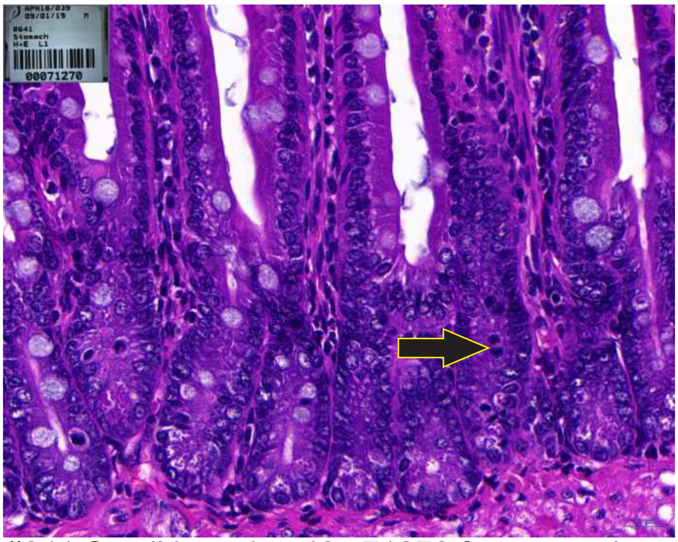

#641 Small intestine 40x 71270 for comparison
Mitotic figures

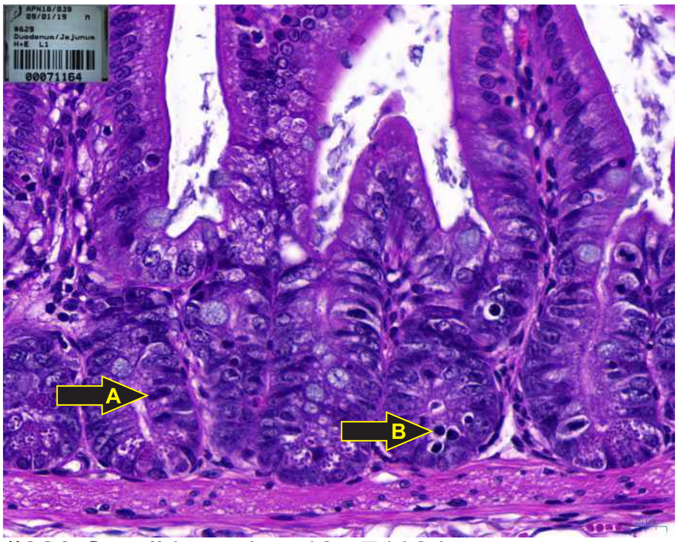

#629 Small intestine 40x 71164
Mitotic figures (A), apoptotic cells (B)

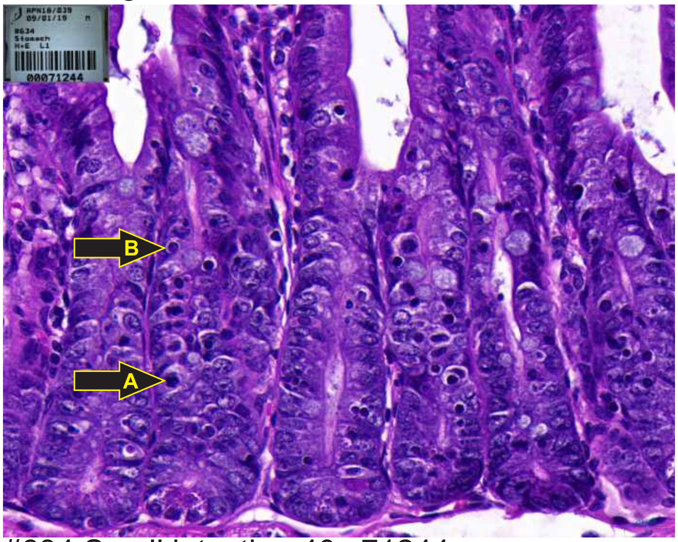

#634 Small intestine 40x 71244
Mitotic figures (A), apoptotic cells (B)

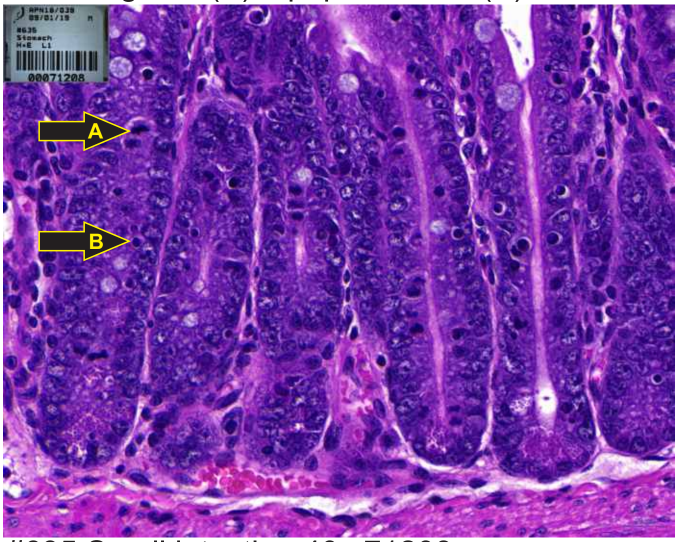

#635 Small intestine 40x 71208
Mitotic figures (A), apoptotic cells (B)

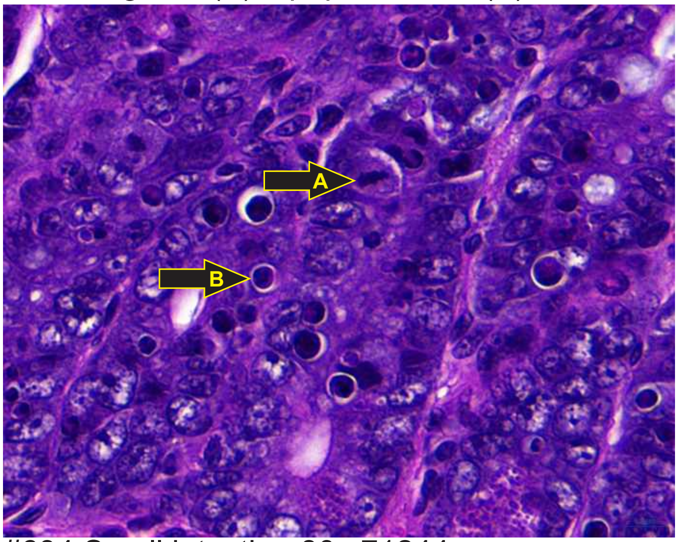

#634 Small intestine 80x 71244
Mitotic figure (A), apoptotic cells (B)

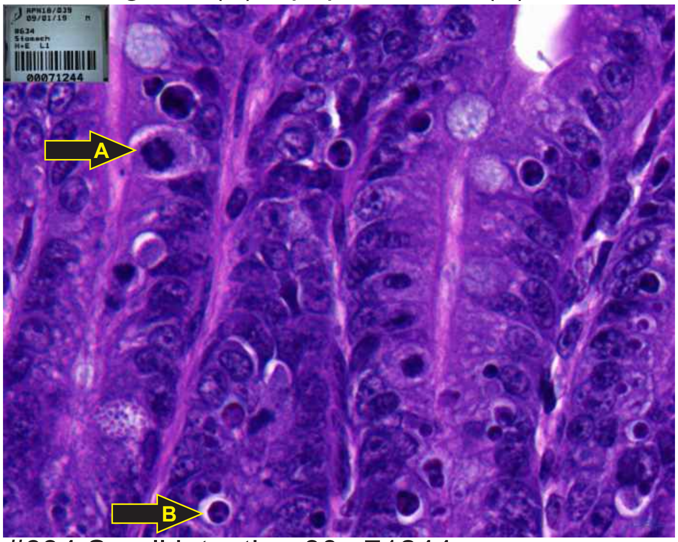

#634 Small intestine 80x 71244
Mitotic figures (A), apoptotic cells (B)

APN18/039SVI
Micro Images

Carl Walkley

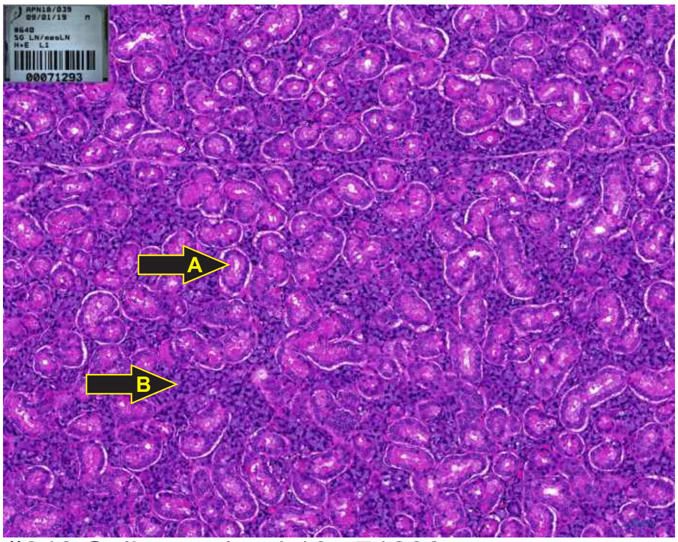

#640 Salivary gland 10x 71293
Normal- ducts (A), acini (B)

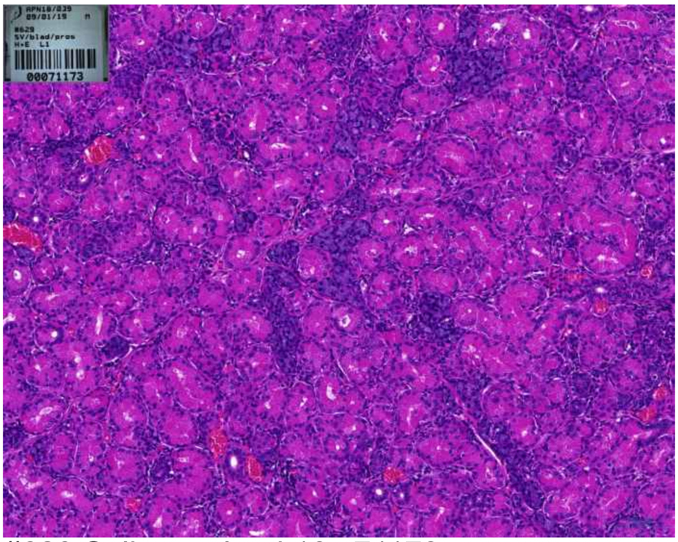

#629 Salivary gland 10x 71173
Less prominent acini

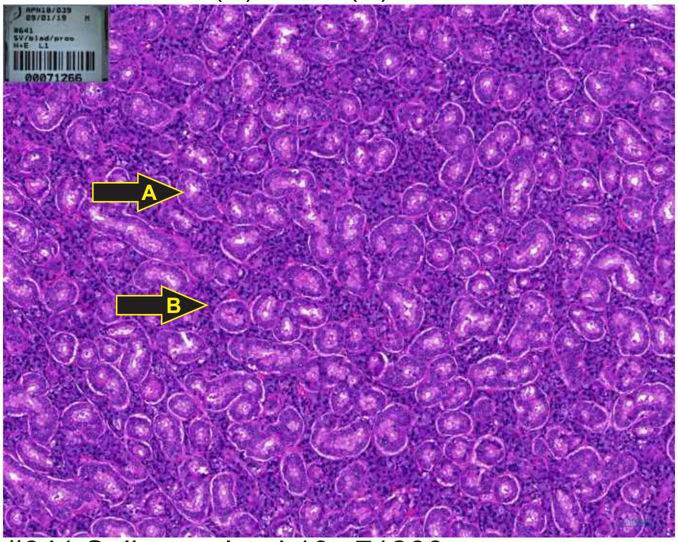

#641 Salivary gland 10x 71266
Normal- ducts (A), acini (B)

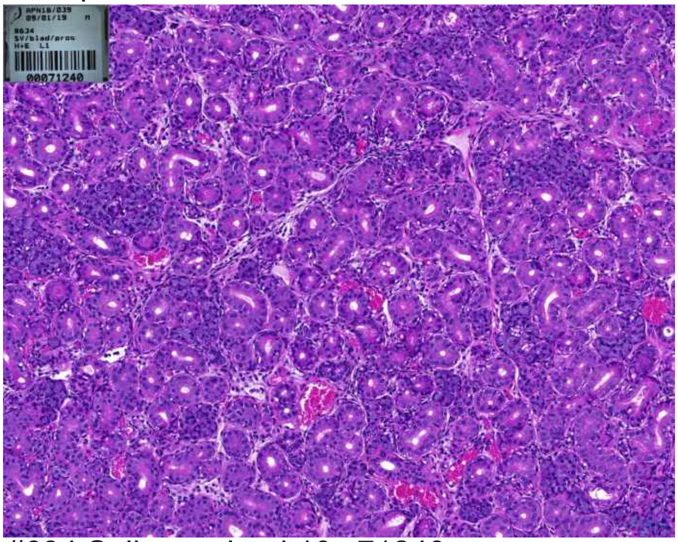

#634 Salivary gland 10x 71240
Less prominent acini

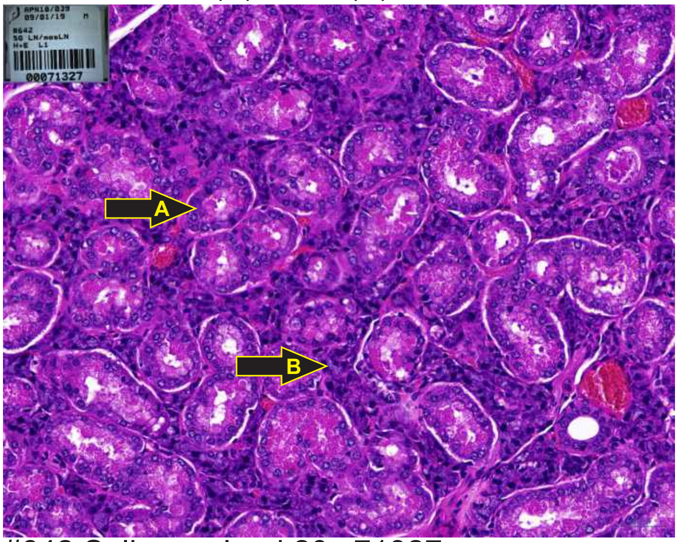

#642 Salivary gland 20x 71327
Normal- ducts (A), acini (B)

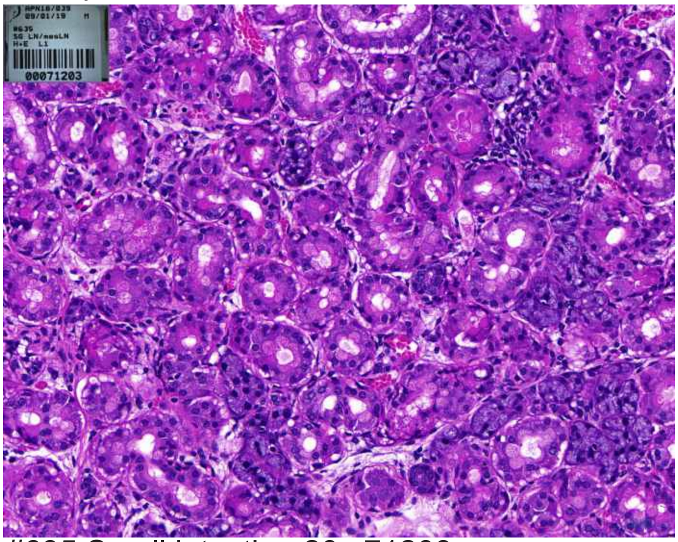

#635 Small intestine 20x 71203
Less prominent acini

APN18/039SVI
Micro Images

Carl Walkley

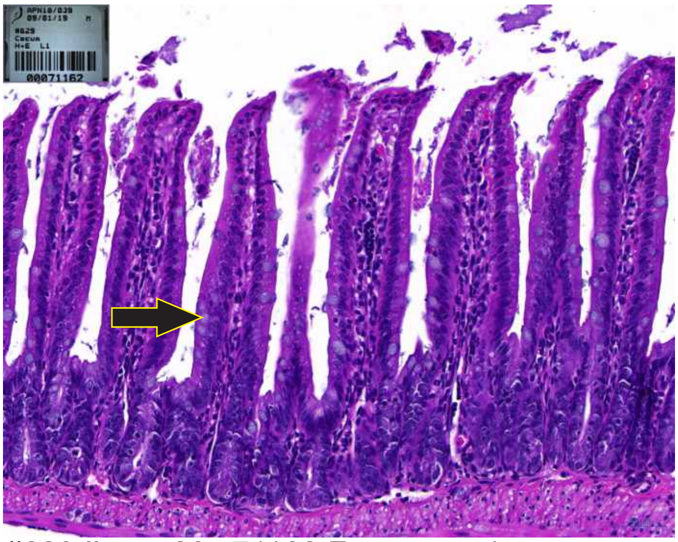

#629 Ileum 20x 71162 For comparison
Goblet cells (GC)

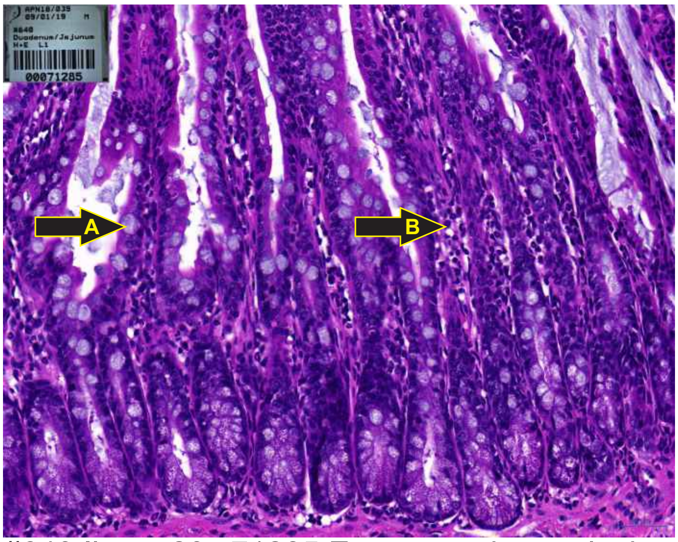

#640 Ileum 20x 71285 Enterocyte hyperplasia
Goblet cells (A), increased immune cells (B)

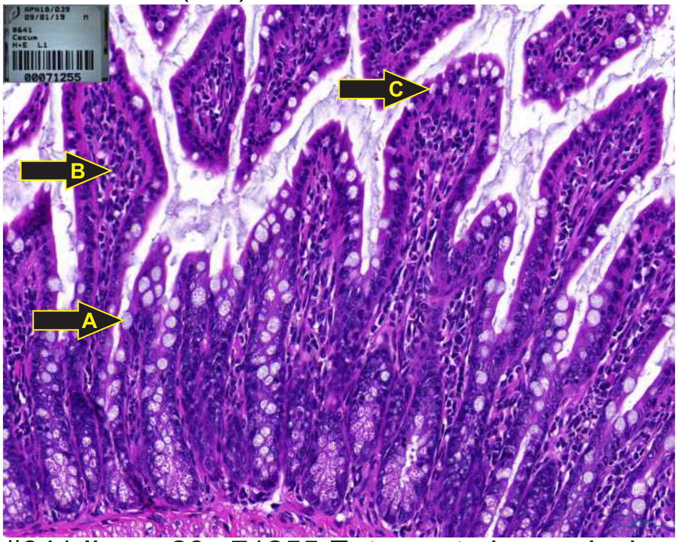

#641 Ileum 20x 71255 Enterocyte hyperplasia
GC (A), increased immune cells (B), vacuolation (C)

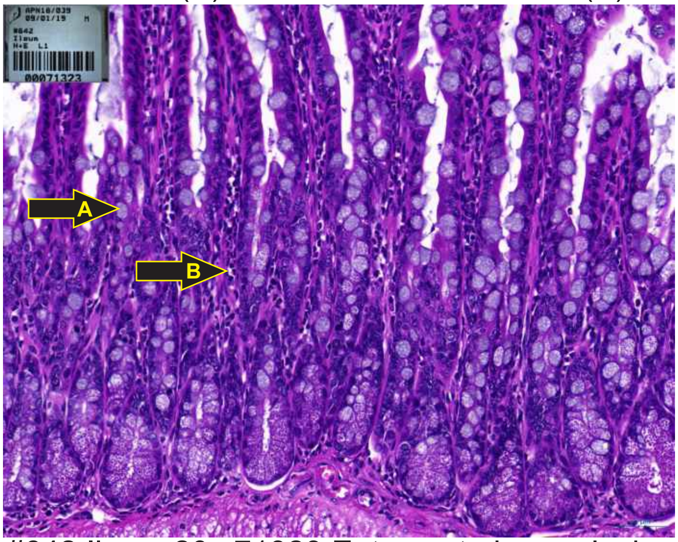

#642 Ileum 20x 71323 Enterocyte hyperplasia
Goblet cells (A), increased neutrophils (B)

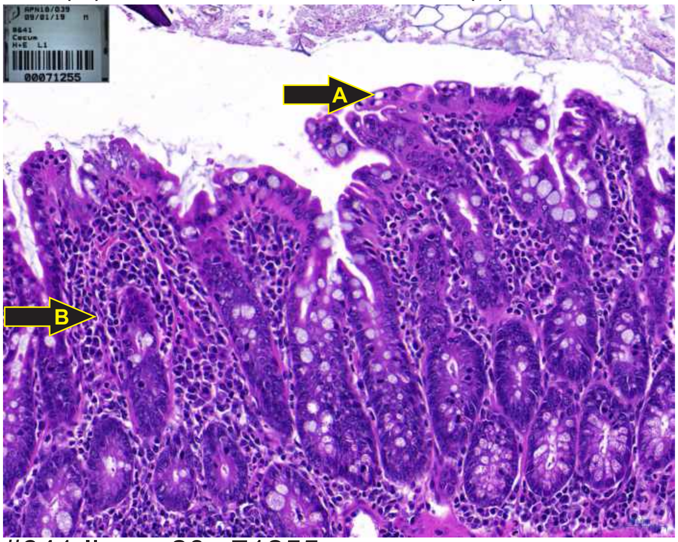

#641 Ileum 20x 71255
Vacuolation (A), increased immune cells (B)

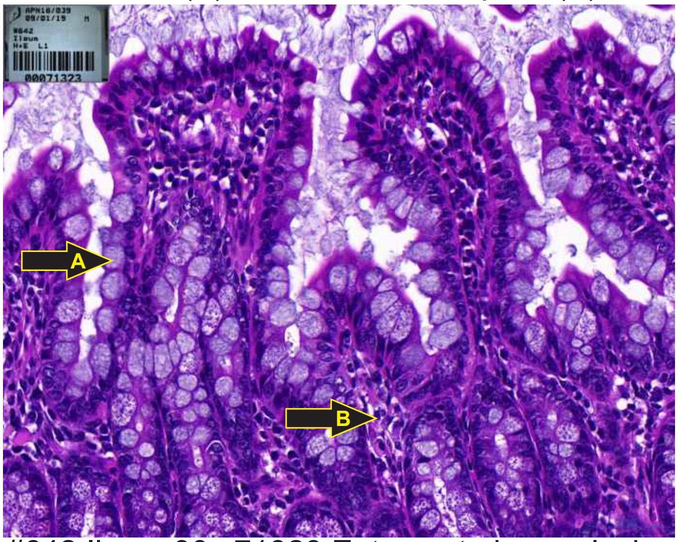

#642 Ileum 30x 71323 Enterocyte hyperplasia
Goblet cells (A), increased immune cells (B)

APN18/039SVI
Micro Images

Carl Walkley

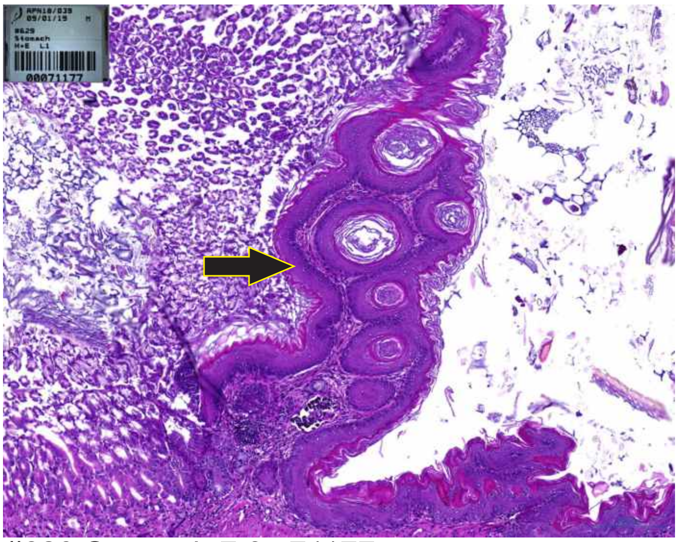

#629 Stomach 7.0x 71177
Limiting ridge - epithelial hyperplasia

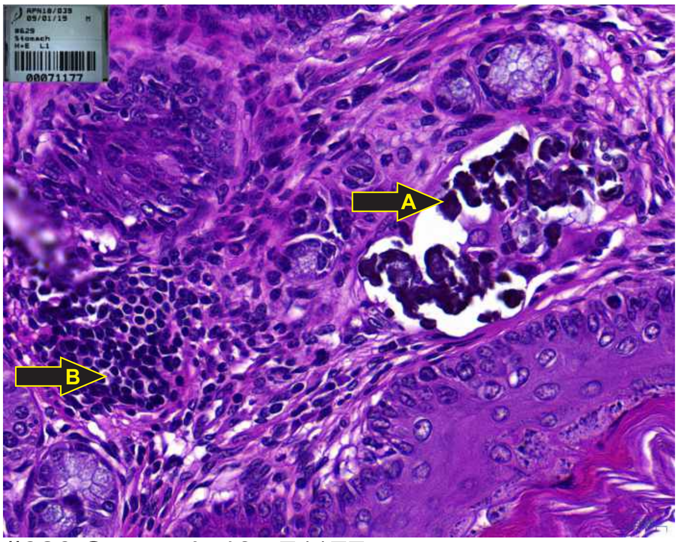

#629 Stomach 40x 71177
Cellular debris (A), mononuclear infiltrates (B)

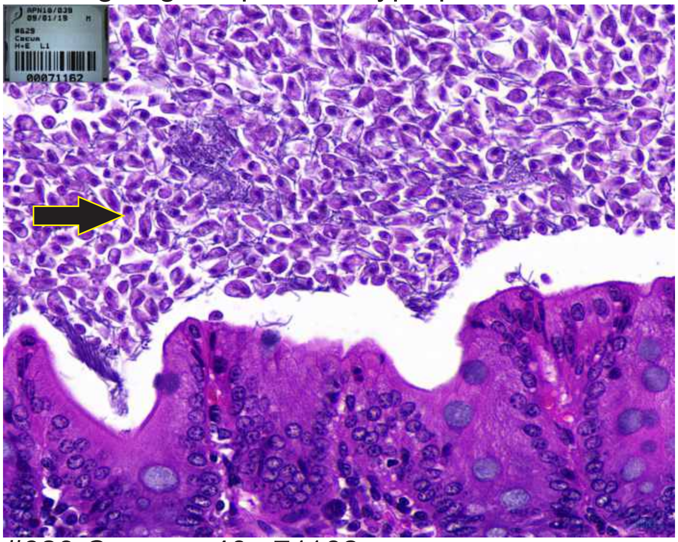

#629 Caecum 40x 71162
Protozoan parasites

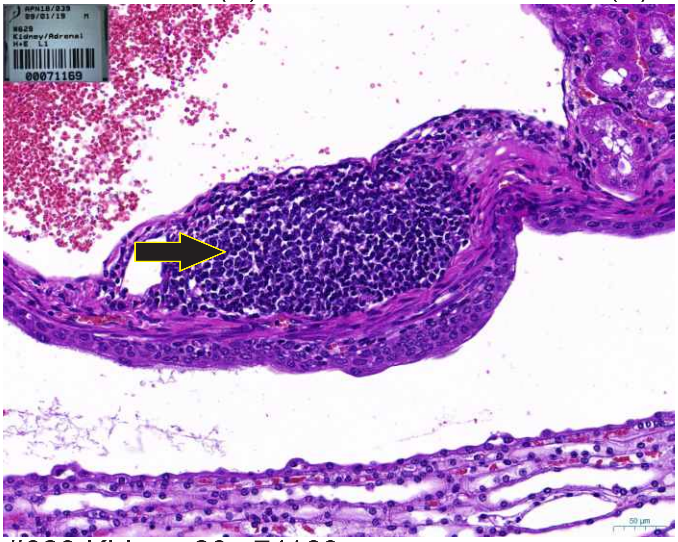

#629 Kidney 20x 71169
Lymphocytic inflammation

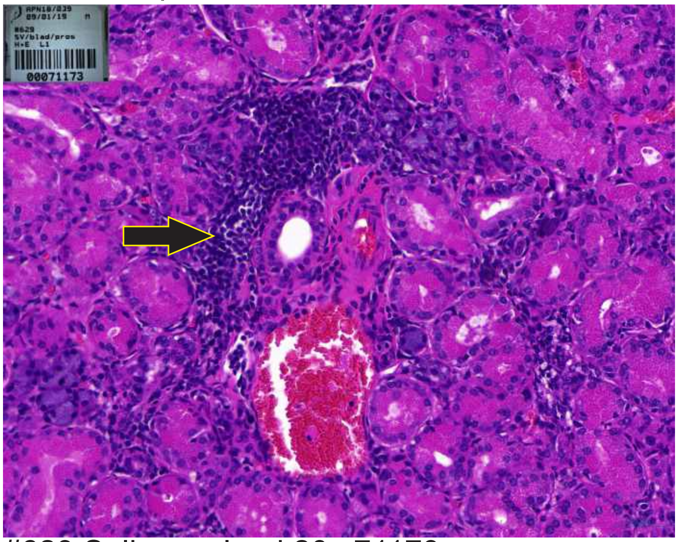

#629 Salivary gland 20x 71173
Lymphocytic inflammation

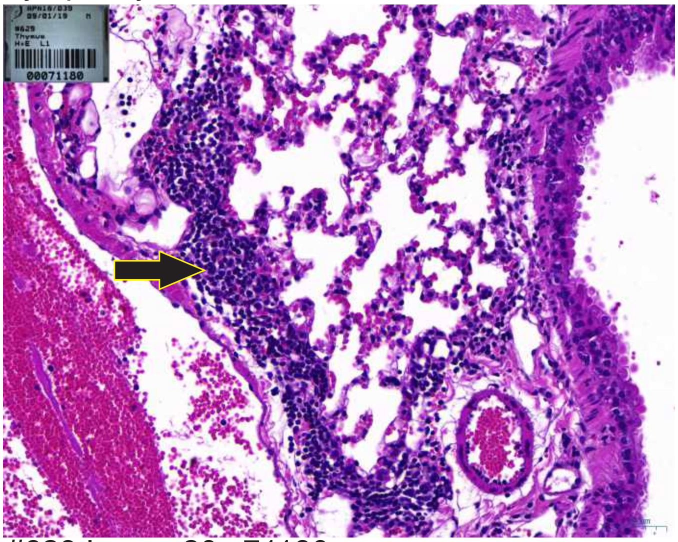

#629 Lungs 20x 71180
Lymphocytic inflammation

APN18/039SVI
Micro Images

Carl Walkley

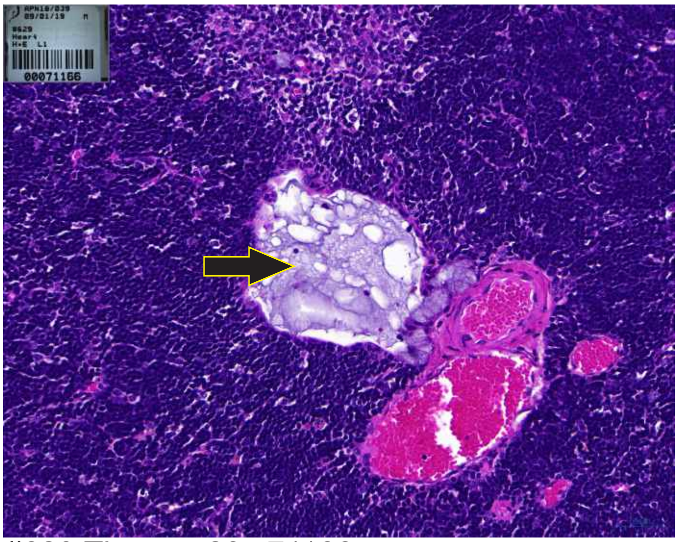

#629 Thymus 20x 71166
Cyst

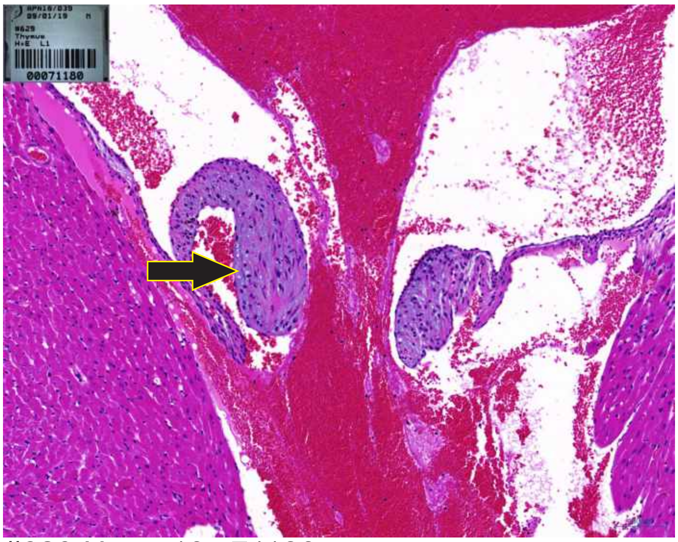

#629 Heart 10x 71180
Myxomatous changes (thickened leaflets)

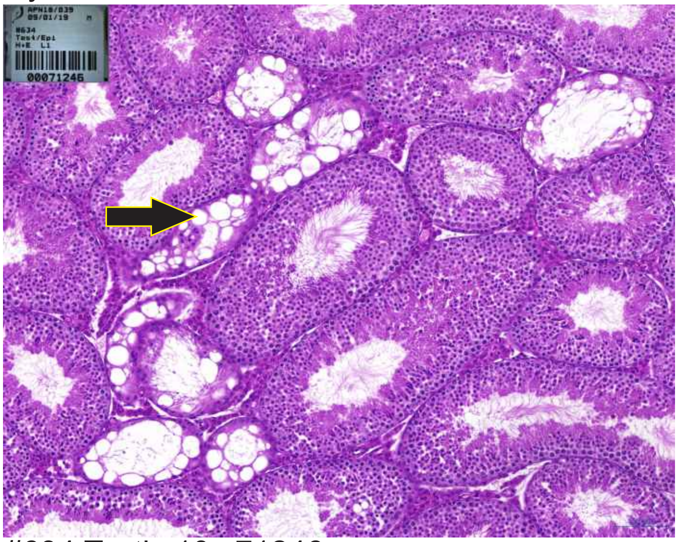

#634 Testis 10x 71246
Testicular degeneration

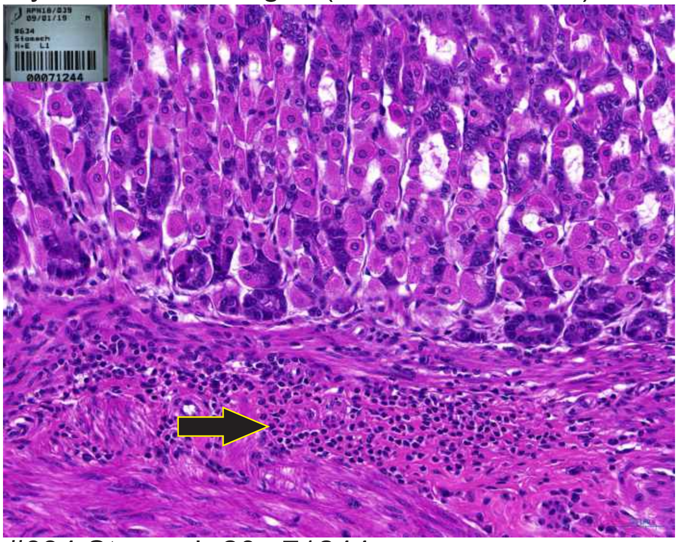

#634 Stomach 20x 71244
Accumulated neutrophils

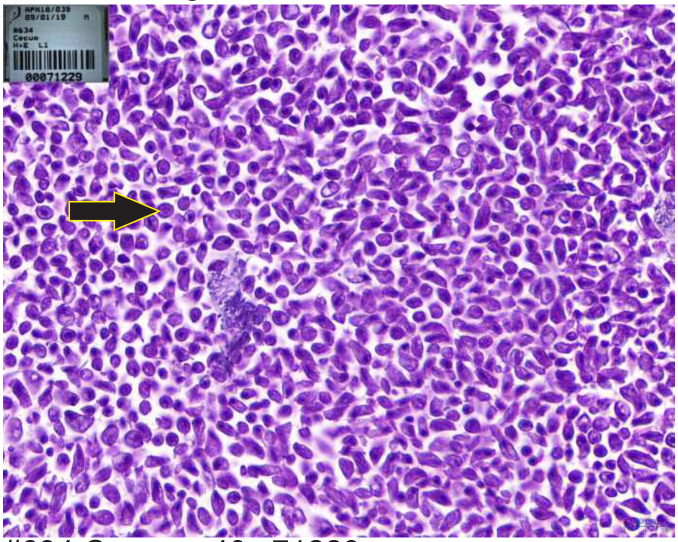

#634 Caecum 40x 71229
Protozoan parasites

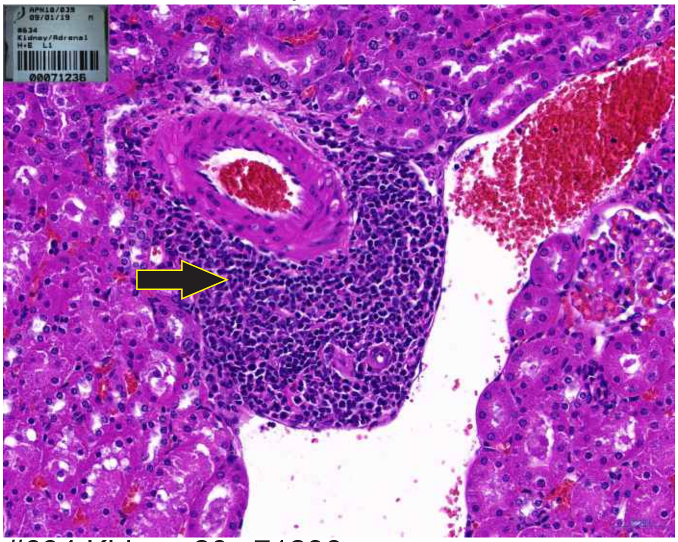

#634 Kidney 20x 71236
Lymphocytic inflammation

APN18/039SVI
Micro Images

Carl Walkley

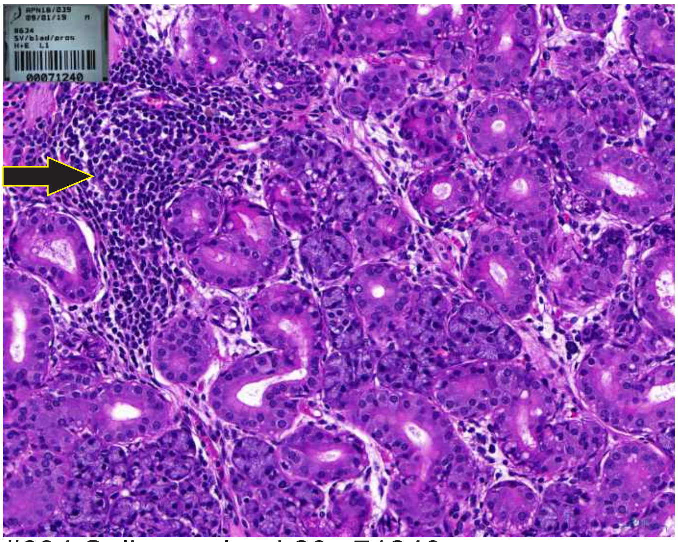

#634 Salivary gland 20x 71240
Lymphocytic inflammation

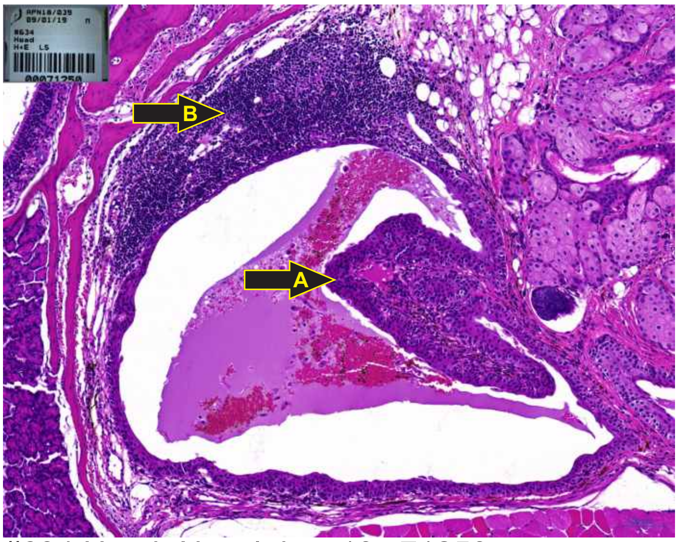

#634 Head- Nasal duct 10x 71250
Epithelial hyperplasia (A), lymphoid tissue (B)

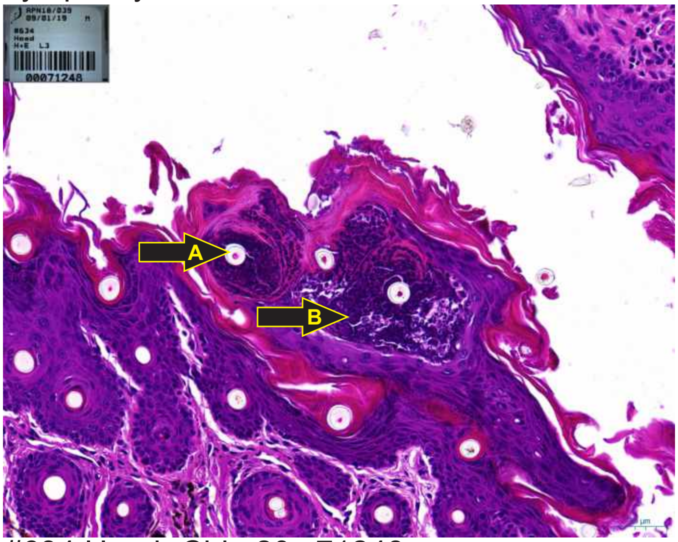

#634 Head- Chin 20x 71248
Hair follicles (A), neutrophilic inflammation (B)

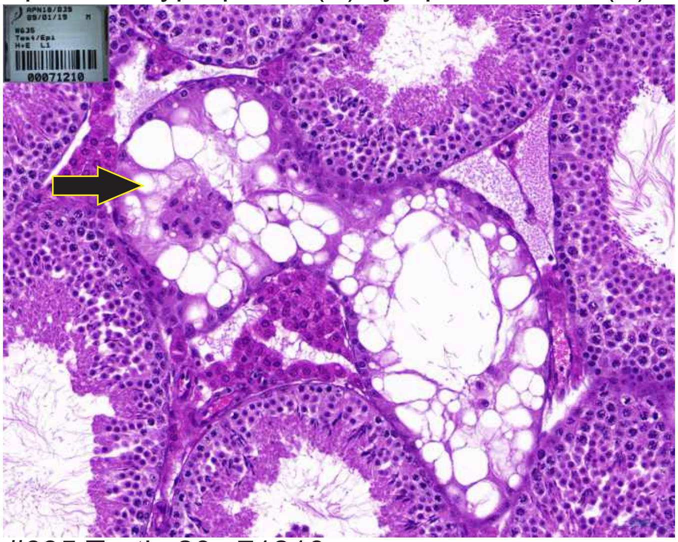

#635 Testis 20x 71210
Testicular degeneration

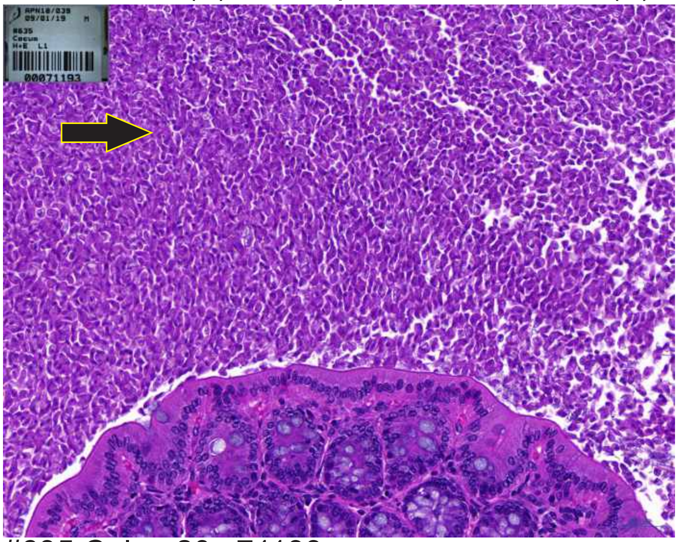

#635 Colon 20x 71193
Protozoan parasites

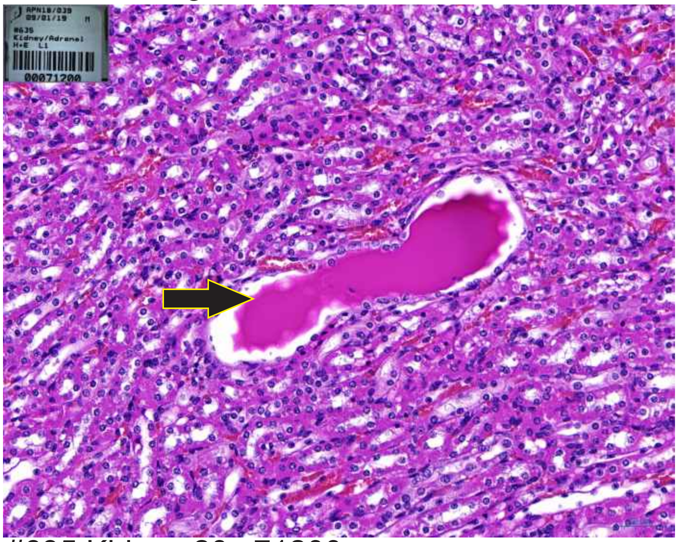

#635 Kidney 20x 71200
Protein cast

APN18/039SVI
Micro Images

Carl Walkley

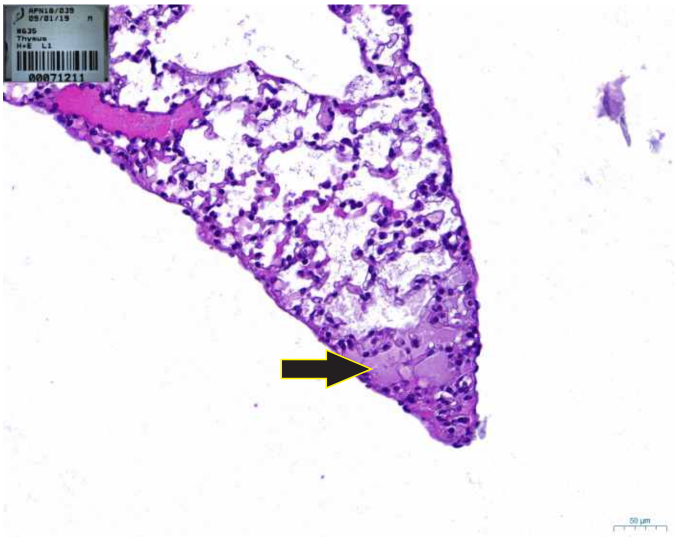

#635 Lung 20x 71211
Intra-alveolar oedema

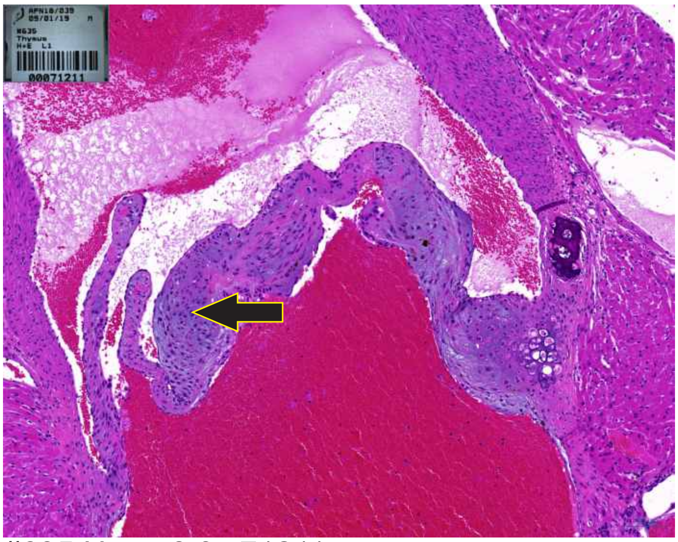

#635 Heart 8.0x 71211
Myxomatous changes (thickened leaflet)

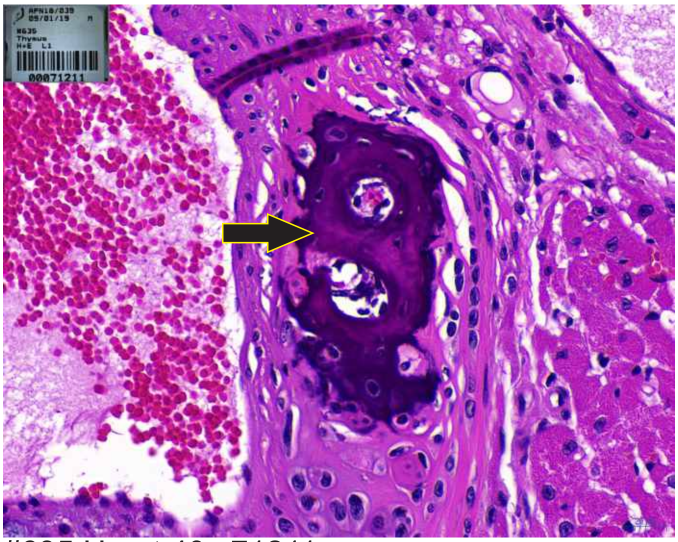

#635 Heart 40x 71211
Mineralisation (calcification)

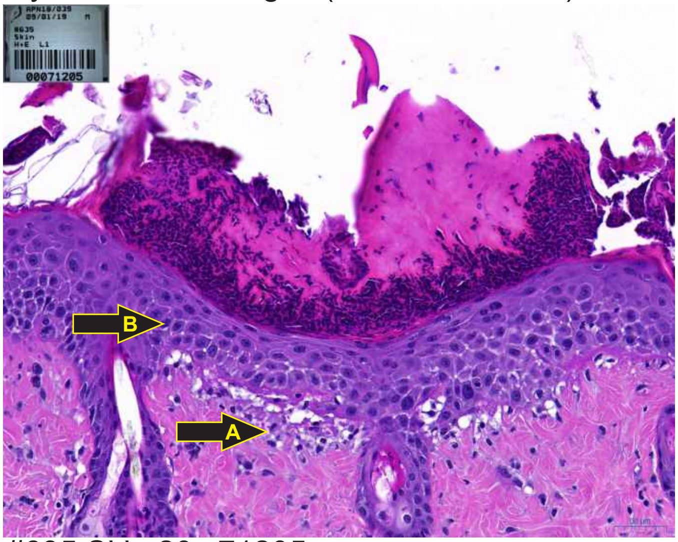

#635 Skin 20x 71205
Inflammation (A), epithelial hyperplasia (B)

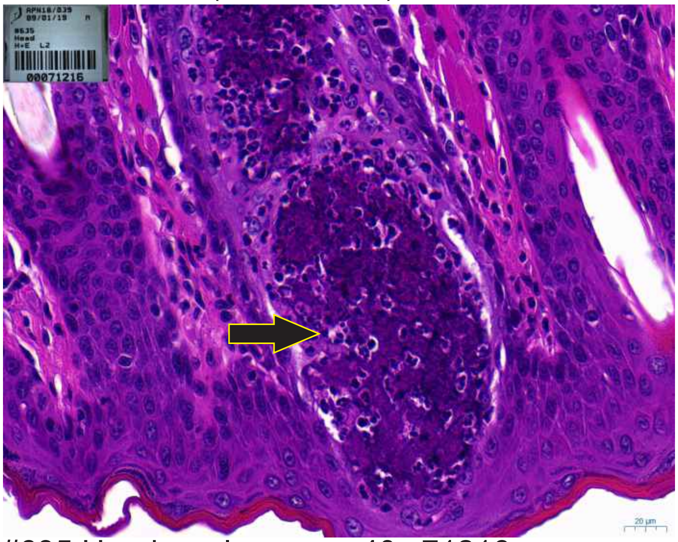

#635 Head- oral mucosa 40x 71216
Neutrophilic inflammation

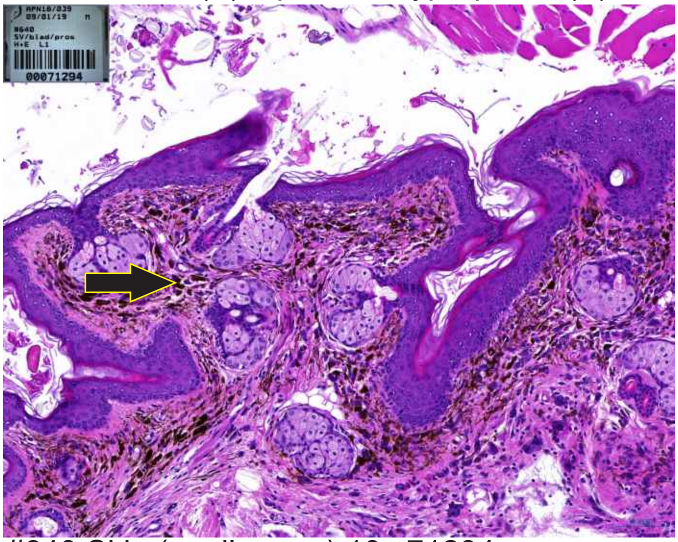

#640 Skin (penile area) 10x 71294
Numerous melanocytes

APN18/039SVI
Micro Images

Carl Walkley

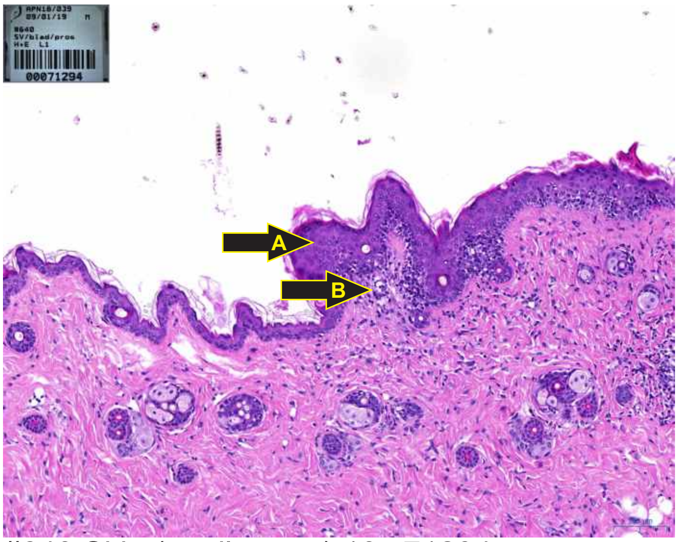

#640 Skin (penile area) 10x 71294
Epithelial hyperplasia (A), inflammation (B)

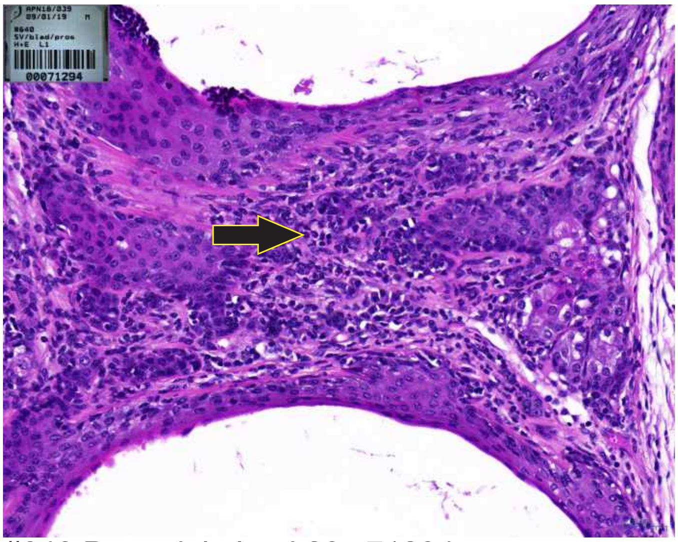

#640 Preputial gland 20x 71294
Inflammation

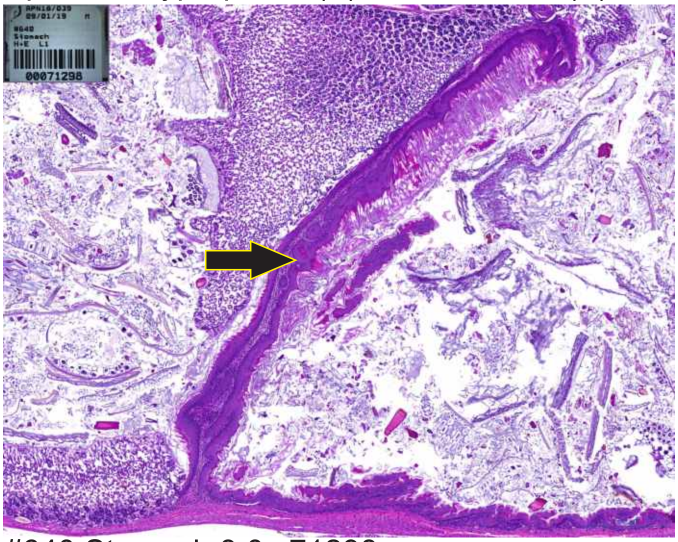

#640 Stomach 3.0x 71298
Epithelial hyperplasia (limiting ridge)

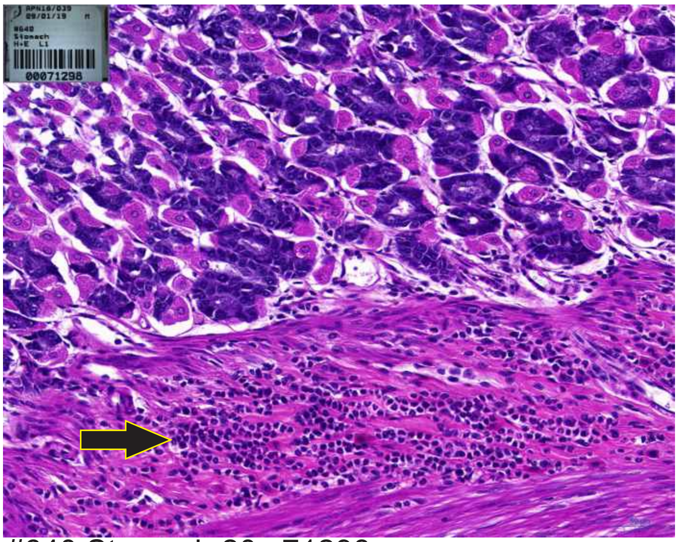

#640 Stomach 20x 71298
Accumulated neutrophils

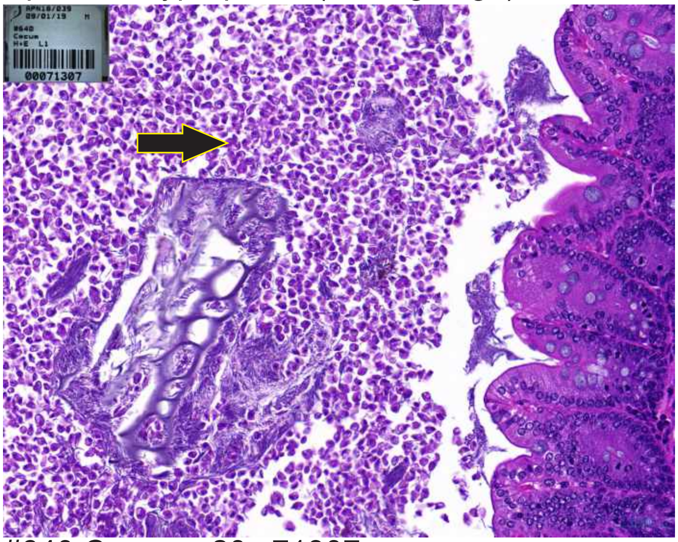

#640 Caecum 20x 71307
Protozoan parasites

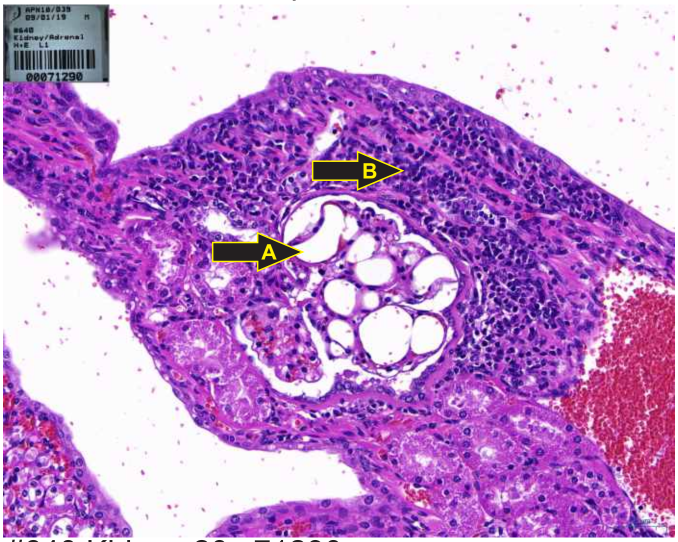

#640 Kidney 20x 71290
Glomerulus- cystic (A), inflammation (B)

APN18/039SVI
Micro Images

Carl Walkley

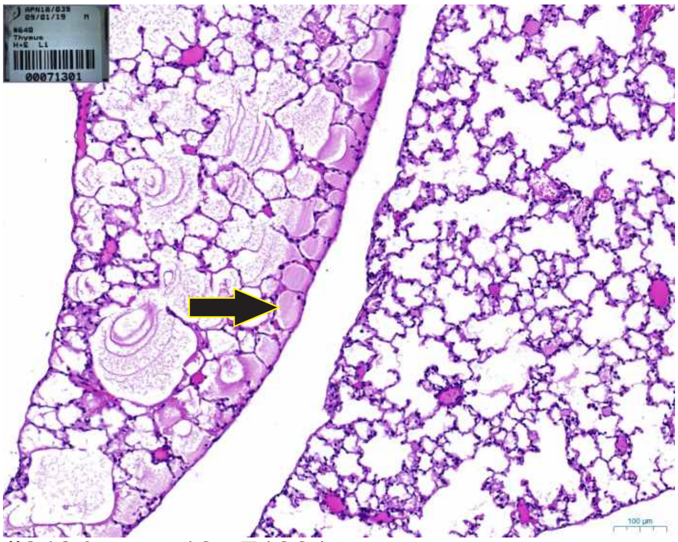

#640 Lungs 10x 71301
Intra-alveolar oedema

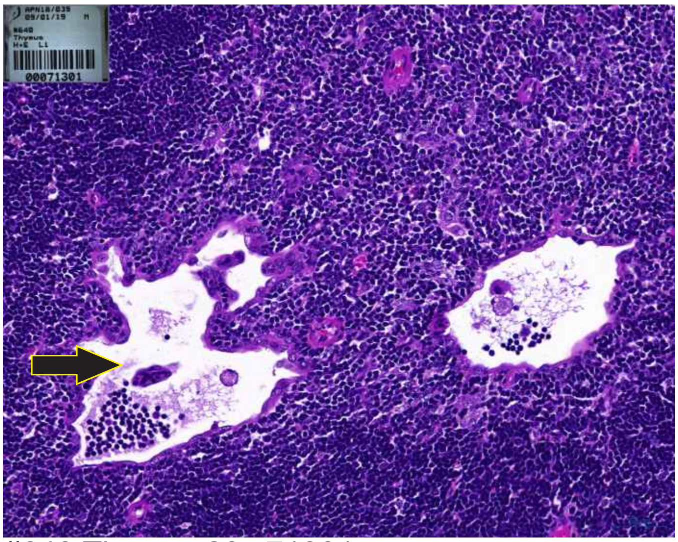

#640 Thymus 20x 71301
Cysts

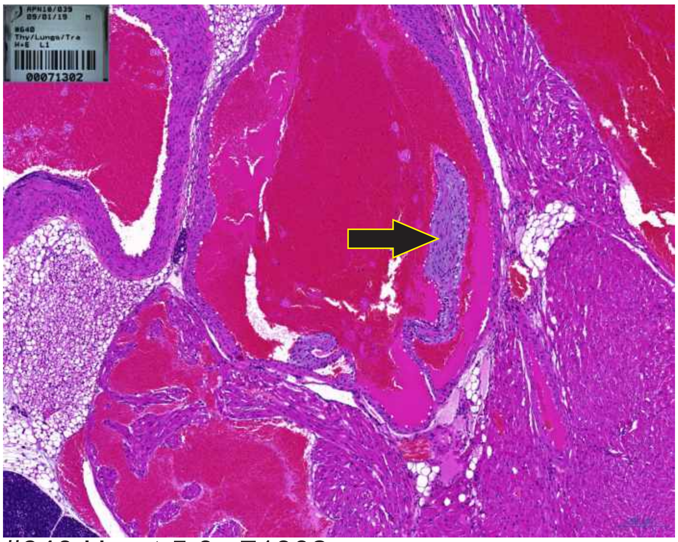

#640 Heart 5.0x 71302
Myxomatous valvular changes (thickened leaflet)

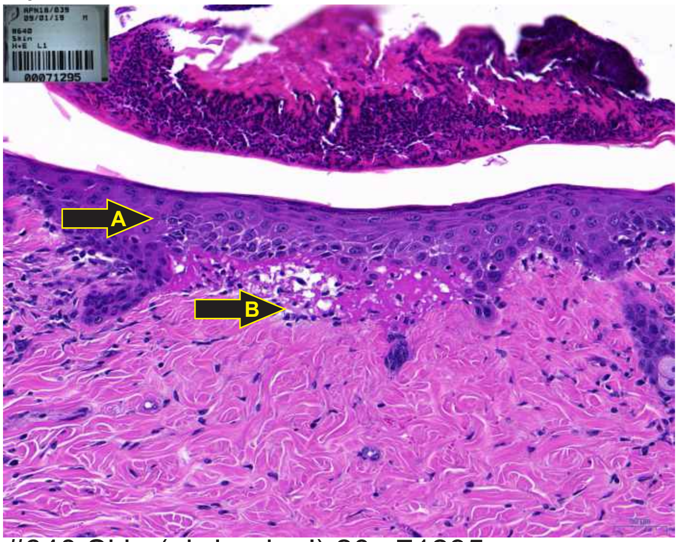

#640 Skin (abdominal) 20x 71295
Epithelial hyperplasia (A), inflammation (B)

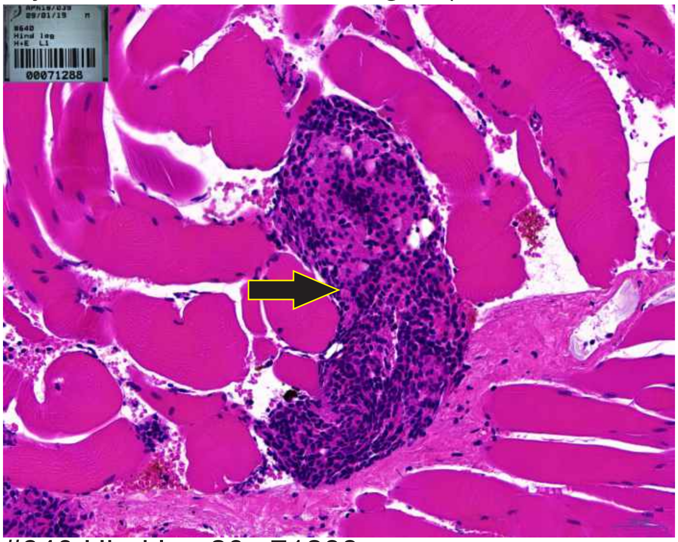

#640 Hind leg 20x 71288
Lymphoid and red blood cells aggregate

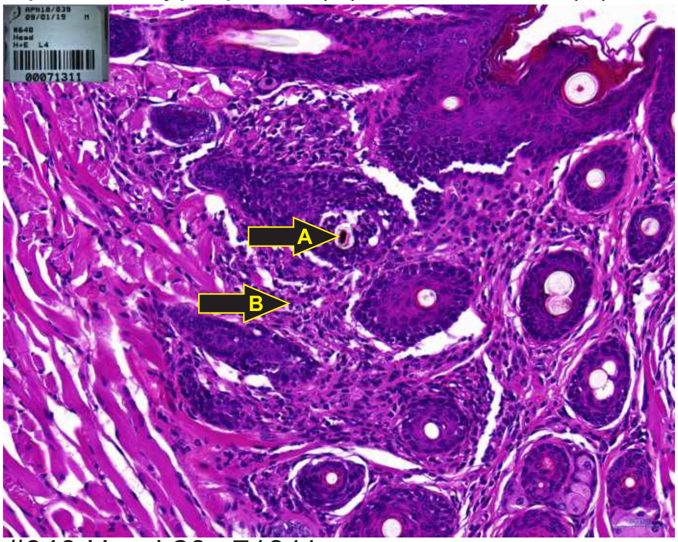

#640 Head 20x 71311
Hair follicle (A), neutrophilic inflammation (B)

APN18/039SVI
Micro Images

Carl Walkley

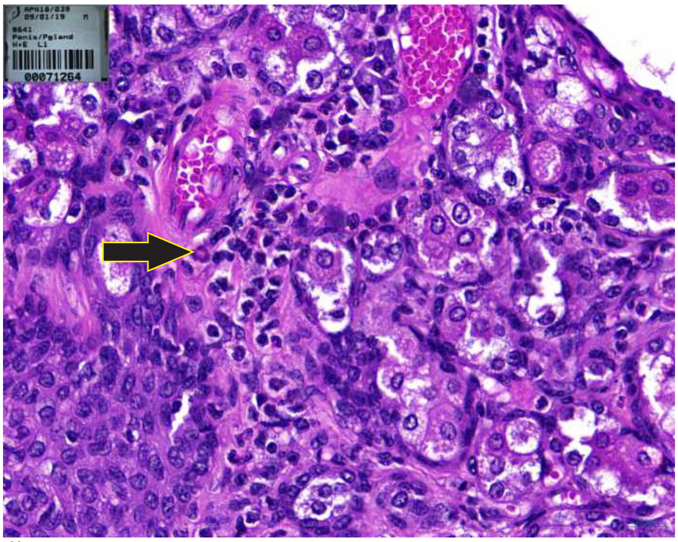

#641 Preputial glands 40x 71264
Neutrophils

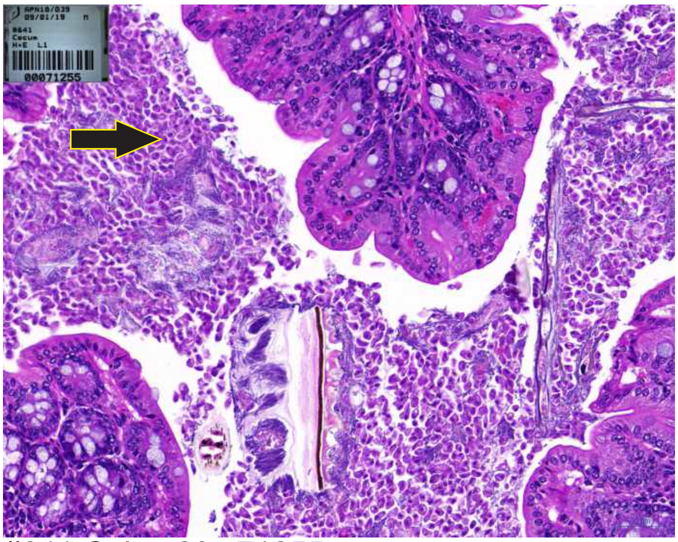

#641 Colon 20x 71255
Protozoan parasites

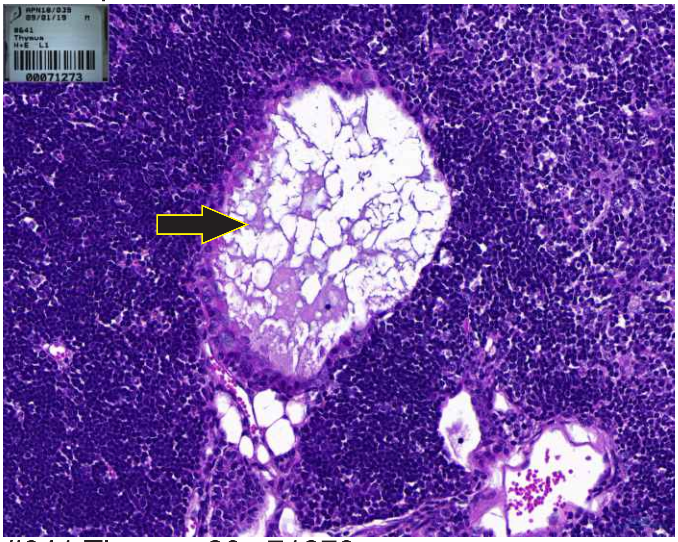

#641 Thymus 20x 71273
Cyst

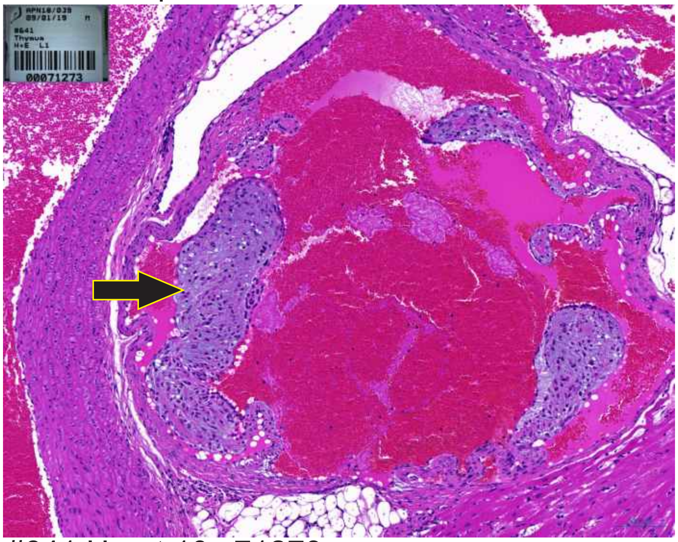

#641 Heart 10x 71273
Myxomatous valvular changes (thickened leaflets)

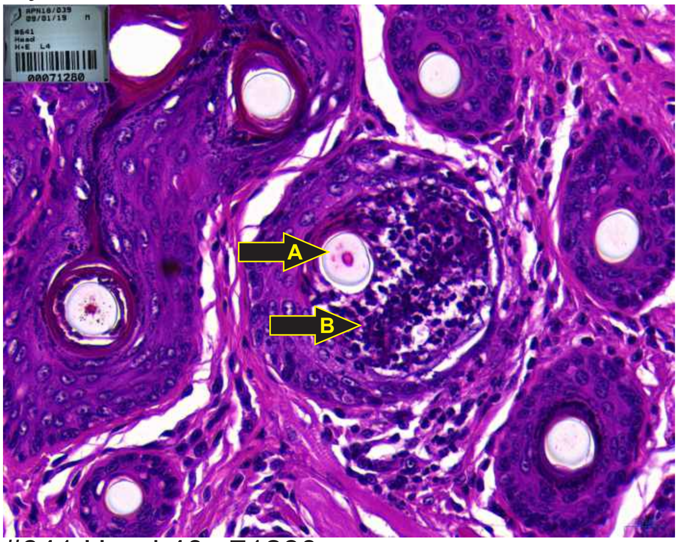

#641 Head 40x 71280
Hair follicle (A), neutrophilic inflammation (B)

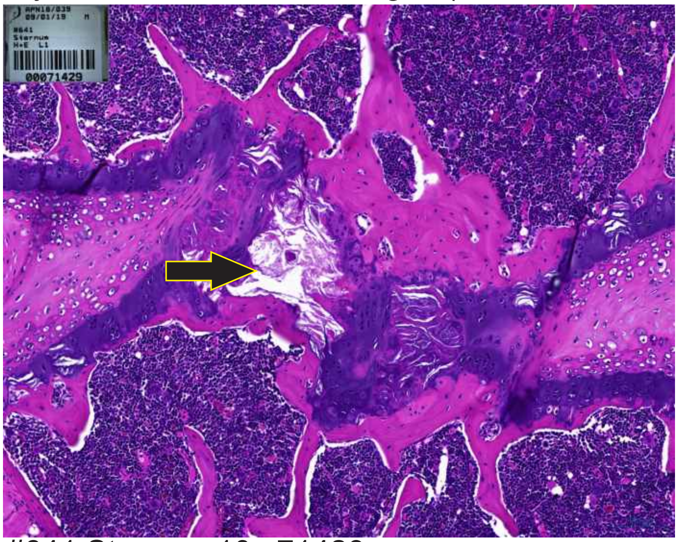

#641 Sternum 10x 71429
Query artefactual disruption of intersternebral joint

APN18/039SVI
Micro Images

Carl Walkley

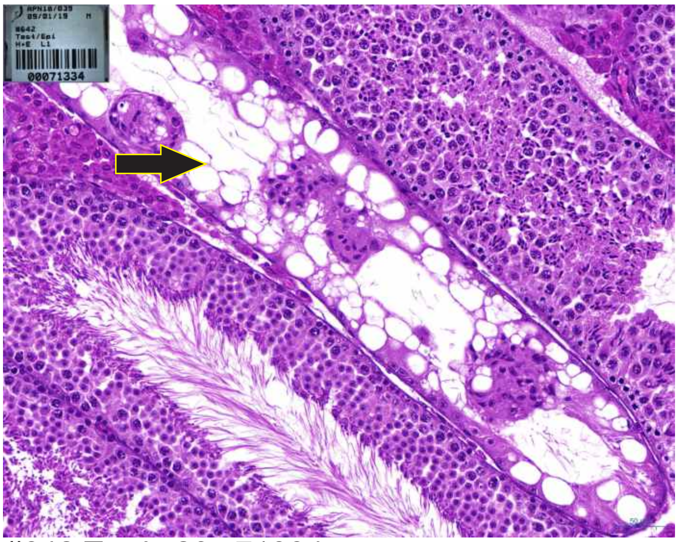

#642 Testis 20x 71334
Testicular degeneration

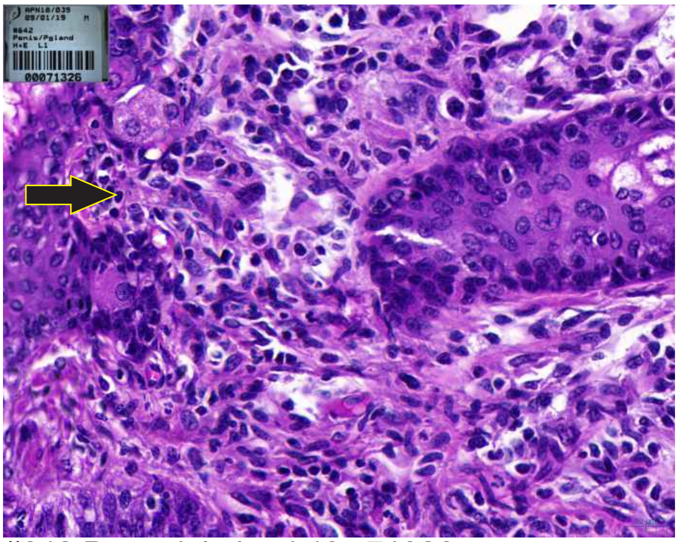

#642 Preputial gland 40x 71326
Inflammation

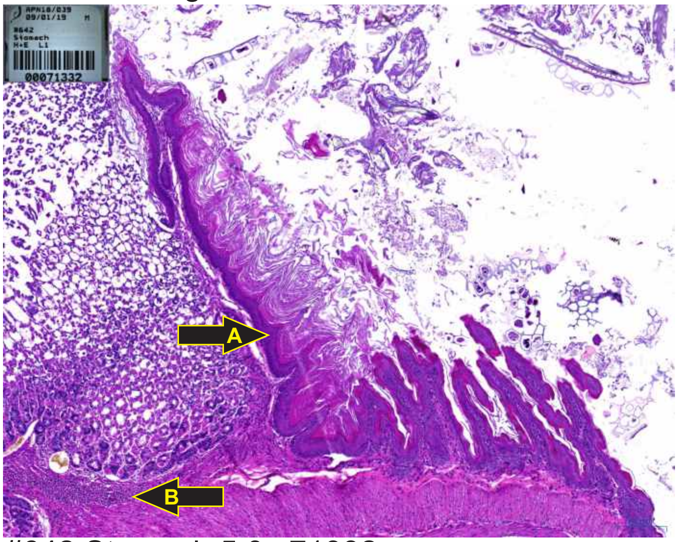

#642 Stomach 5.0x 71332
Epithelial hyperplasia (A), inflammation (B)

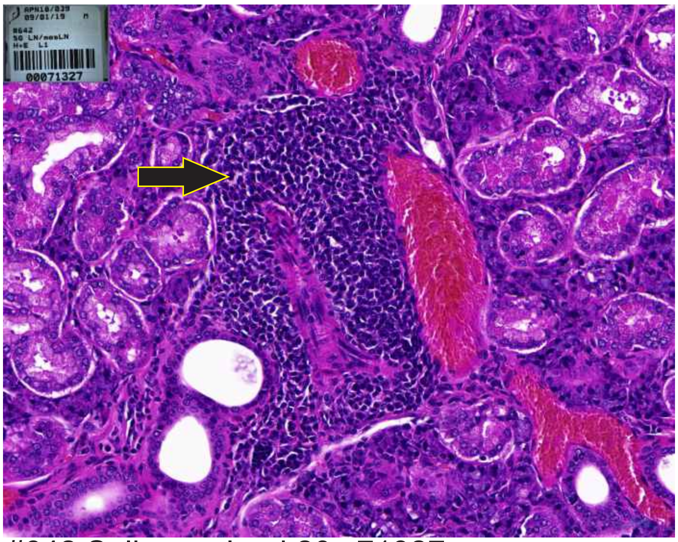

#642 Salivary gland 20x 71327
Lymphocytic inflammation

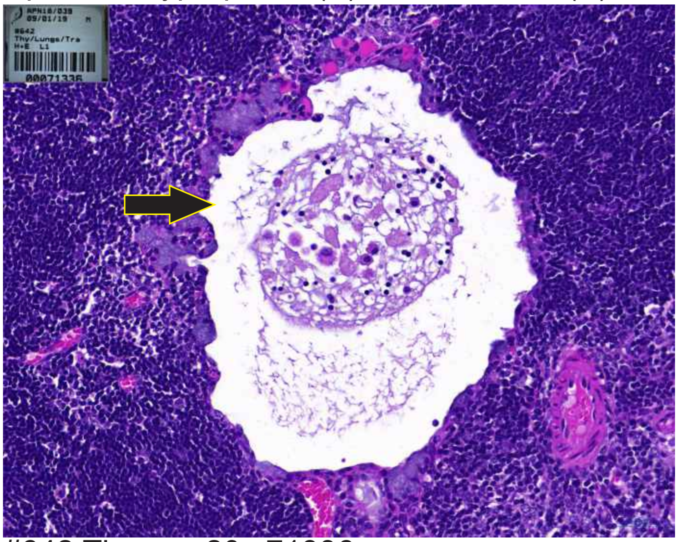

#642 Thymus 20x 71336
Cyst

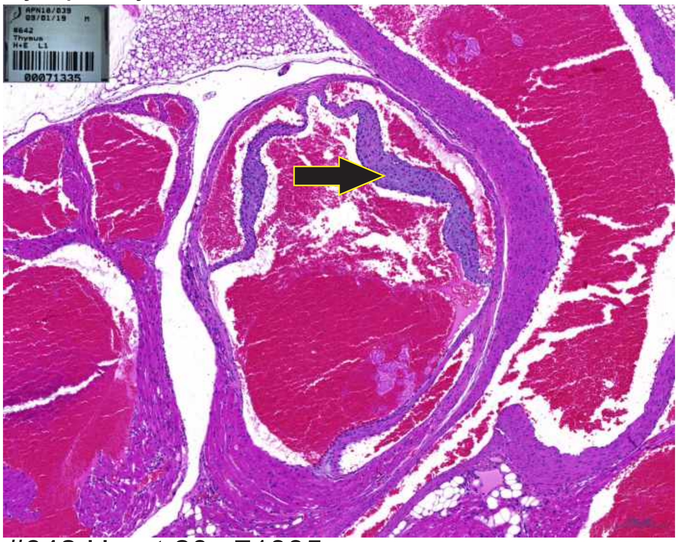

#642 Heart 20x 71335
Myxomatous changes (thickened leaflets)
