# Supplementary material for: The majority of A-to-I RNA editing is not required for mammalian homeostasis
Source: Genome Biol. 2019 Dec 9;20:268. doi: 10.1186/s13059-019-1873-2 (PMC6900863; doi:10.1186/s13059-019-1873-2)
Supplement: Supplementary file 8 — Additional file 8: Review history. [file 13059_2019_1873_MOESM8_ESM.docx]

Review History

**First round of review**

**Reviewer 1**

**Are you able to assess all statistics in the manuscript, including the appropriateness of statistical tests used?**

Yes, and I have assessed the statistics in my report.

**Comments to author:**

The manuscript "The majority of A-to-I RNA editing is not required for mammalian homeostasis" is the summary of an elegant experiment that show that the main function of A-to-I RNA editing is to inhibit false activation of the innate immunity by editing endogenous dsRNAs. Main conclusion of the manuscript is that beside GLUR2, there are no additional essential sites in mammalian.

The paper is easy to read and clearly summarized the research. It is defiantly an important contribution to the field.

While the conclusion is a direct outcome of the findings, I think it will be fair to claim that it is well possible that some of RNA editing sites are important but not essential - some very nice examples were published in the last few years and I believe they should be discussed (NEIL1, FLNA,AZIN1). It will be very strange to find that the evolutionary conserved sites are not important.

Moreover, mouse has an order of magnitude less editing events than primates, thus it is well possible that the picture will be different in human- I believe this point should be discussed.

Additional few suggestions:

Line 114: "The physiological contribution of other protein recoding events outside of Gria2 and the reasons for the evolutionary conservation of a subset of them is unknown". I don't sure I fully agree to this statement, several sites such as NEIL1, FLNA,AZIN1 has some very important function, as revealed in the last few years.

Line 120: "inosine is most abundant in brain mRNA (43)." Recent literature show that artery is probably the most edited tissue (Jain et al EMBO J. 2018)

Line 268: "Such a phenomena has previously been reported in C.elegans"- it was reported in the past for few cases in mouse as well by the group of M. Jantsch (PMID: 18430892)

Line 286: "We then defined the genotype specific editing events from the entire dataset and assessed the characteristics of these sites". One needs to compare current results to previous studies who also reported the ADAR responsible for editing of sites (e.g Tan, jantsch )- I think there are some conflicts (e.g BLCAP) between current results and the published dada.

Line 278: "Using definitive genetic controls, we conclude that Adar1 and Adar2 have similar editing site distributions" - The data show clearly that there are set of conserved site that edited only by ADAR2- how this fit to the said conclusion?

The author can use their data to evaluate how accurate the RNA editing databases are, and how many of the deposited sites are indeed real editing events. This will be of high importance for the community, specifically about the coding sites

-The nomenclature is not consistent - the authors use both names of the enzyme, (adarb1 and adar2) throughout the manuscript. In order to avoid confusion- one name should be selected.

**Reviewer 2**

**Are you able to assess all statistics in the manuscript, including the appropriateness of statistical tests used?**

Yes, and I have assessed the statistics in my report.

**Comments to author:**

This work is an important contribution to our understanding of the role of editing in mammalian physiology. I would like very much to see it published, and believe it is going to be a well-cited reference for all the RNA editing community.

Therefore, I think it is of utmost importance to be very careful about the results and their interpretation. In addition, the data (which will be certainly further used by many) should be presented as fully as possible. Accordingly, I would like to make the following comments/requests (in no particular order):

1. A general comment: the authors show that ADAR-deficient animals seem to live and age normally under standard housing conditions. This shows that there are no essential editing sites (except for the GluRb Q/R one). However, this does not mean that "the majority of A-to-I editing is stochastic" (as stated in the conclusion), certainly not for recoding sites. It is very well possible that the phenotype of editing are subtle or required only under specific consequences. In fact, Horsch et al (Ref. 26) have identified some changes due to the absence of ADAR2, Jain et al (EMBO J 2018) have shown the effect of impaired FLNA editing, and so forth. Please discuss your results in the context of the above results, as well as the conservation of multiple recoding sites AND their ECS, and the enrichment of non-synonymous conserved recoding sites, both being a strong signal for positive selection.

Similar statements are scattered throughout the paper (e.g. "the global absence of protein recoding does not have a deleterious physiological effect in vivo"). Please rephrase your conclusions and interpretations to avoid over-interpretation.

2. " Editing can occur at specific adenosines within a transcript, termed site selective editing, or at many sites within clusters, termed hyperediting or editing enriched regions" -- "clusters of sites" may mean different things. Most noncoding editing loci harbor clusters of sites, in the sense that there are multiple potentially-edited sites within a small region. However, the typical RNA molecule transcribed from these regions is edited in only one (or none) of these locations. The term hyper-editing usually refers to cases where a single molecule harbors multiple inosines. Anyway, please define clearly the terms you use.

3. Fig 1a-b shows the Mendelian ratios for ADAR1-het; ADAR2-null mice against themselves, or against ADAR1-null; ADAR2-null. The reader would like to see the full picture, including crossing ADAR1-het; ADAR2-het with themselves and all other combinations for which data is available. The methods should provide more information on the route through which the dKO mice were produced, beyond the general statement "we crossed the viable rescued Adar1-editing deficient animals (Adar1E861A/E861AIfih1-/-) (30, 38) with the Adar2-/-Gria2R/R animals (25)".

4. Fig 1c - data for control mice (Ifih-/-, but having both ADARs functional) is missing. The triangular showing p-values should be rearranged to make the clustering into two groups more obvious. The same ordering should then be used in the legend above (to my color-vision challenged eyes, it is not so easy to connect the colors to the descriptions).

5. Figure 1E - What is the p-value? (it might be low but NS, suggesting a bigger sample size could show a difference). Again, add data for Ifih-/-ADAR+/+ADAR2+/+ (if available), or at least for WT mice.

6. Figure 3 - again, various interesting comparisons are missing. Can you show the diff. expression of dKO against E861A (indirectly shown in 1E) ? This could highlight the effect of ADAR2 editing over the background of immune system changes. Other combinations could be interesting too. Four of the ten comparisons (each of the 5 genotypes against all others) appear in Figure 3, please calculate the others and then choose which one should appear in main text and the put the others as supp. figure.

7. The above comparisons do show a small number of genes that change substantially (in each case). Please elaborate.

8. Figure 4b: a few dots show (apparent) editing for the dKO. Please identify the sites and explain.

9. Please explain (in Methods or elsewhere) how you decide a site to be selective or hyper-edited (see also point #2 above)

10. "Despite the single mutant phenotypes, there was no clear trend toward editing preference for repetitive elements for sites edited by Adar1 nor toward site-selective/recoding for Adar2 (Fig 5b)" However, Figure 5b and 5c do not provide data on repetitive elements. Please add numbers for B1 and B2 regions, known to harbor much of the mouse editing sites.

11. "This same activity is now being exploited for targeted reprogramming of transcripts for potential therapeutic benefit (24, 42)." This sentence seems out of place. I don't see how it connects to what follows.

12. " Although one third of editing sites in brain were shared by Adar1 and Adar2, including many that are evolutionarily conserved and within coding regions, mice completely lacking A-to-I editing developed and aged normally. This demonstrates that Adar1 and Adar2 do not physiologically compensate in vivo." I think I understand what these sentences try to say: one could have thought that there are some essential sites among the many sites edited by both ADARs, but we now see there aren't. However, the sentences are not clear, please rephrase. In particular, what you show is not that "Adar1 and Adar2 do not physiologically compensate" (actually they do, in the sense of editing a list of shared substrates), but rather that there is no essential need in compensation. So either the shared substrates have no function at all, and then there is no need in compensation, or they do have a function that is not essential under normal housing, and then they do compensate.

13. "Physiologically essential A-to-I editing comprises a small subset of the editome and that the majority of editing is dispensable for mammalian homeostasis. Moreover, in vivo biologically essential protein recoding mediated by A-to-I editing is an exception in mammals." -- if by "a small subset" you refer to the GluRB Q/R site only, I think you should be more specific. In addition, some caution should be taken when extrapolating from mouse to all mammals.

**Authors Response**

**Point-by-point responses to the reviewers’ comments:**

Reviewer #1: The manuscript "The majority of A-to-I RNA editing is not required for mammalian homeostasis" is the summary of an elegant experiment that show that the main function of A-to-I RNA editing is to inhibit false activation of the innate immunity by editing endogenous dsRNAs. Main conclusion of the manuscript is that beside GLUR2, there are no additional essential sites in mammalian.

The paper is easy to read and clearly summarized the research. It is defiantly an important contribution to the field.

While the conclusion is a direct outcome of the findings, I think it will be fair to claim that it is well possible that some of RNA editing sites are important but not essential - some very nice examples were published in the last few years and I believe they should be discussed (NEIL1, FLNA,AZIN1). It will be very strange to find that the evolutionary conserved sites are not important.

Moreover, mouse has an order of magnitude less editing events than primates, thus it is well possible that the picture will be different in human - I believe this point should be discussed.

*We thank the reviewer for their constructive, positive assessment of our work. We would like to clarify that we believe there are two sets of essential editing sites: Gria2 Q/R site for ADAR2 and the unedited transcripts that become MDA5 substrates in the absence of ADAR1-mediated editing. We have amended the text to make this conclusion/interpretation clearer.*

*We acknowledge the reviewer’s comments regarding the possibility of functions for recoding sites – we stated this in the original text on line 346-348 and lines 361-363. The reviewer’s statement is correct that we have tested the essentiality of recoding at all sites outside of GRIA2 using the most robust genetic analysis possible. The in vivo genetic experiment has yielded an unexpectedly unambiguous result, indicating that completely editing deficient mice are largely normal (within the parameters assessed). We believe that our interpretations as presented are a reflection of the definitive nature of the genetic result. The relative numbers of editing events between species is a relevant point that we have now included within the discussion. It is equally relevant that the consequences of loss of ADAR1, for example, are the same in mouse and human (ie an MDA5 dependent activation of the innate immune response) despite the large difference in editing levels. We have addressed the comments as outlined below and in the main text.*

Additional reviewer comments:

Line 114: "The physiological contribution of other protein recoding events outside of Gria2 and the reasons for the evolutionary conservation of a subset of them is unknown". I don't sure I fully agree to this statement, several sites such as NEIL1, FLNA, AZIN1 has some very important function, as revealed in the last few years.

*Author Reply: We have added additional text as requested.*

Line 120: "inosine is most abundant in brain mRNA (43)." Recent literature show that artery is probably the most edited tissue (Jain et al EMBO J. 2018).

*Author Reply: We have modified the text.*

Line 268: "Such a phenomena has previously been reported in C.elegans"- it was reported in the past for few cases in mouse as well by the group of M. Jantsch (PMID: 18430892).

*Author Reply: We have included the additional reference.*

Line 286: "We then defined the genotype specific editing events from the entire dataset and assessed the characteristics of these sites". One needs to compare current results to previous studies who also reported the ADAR responsible for editing of sites (e.g Tan, Jantsch) - I think there are some conflicts (e.g BLCAP) between current results and the published dada.

*Author Reply: There are always going to be differences between datasets when any single example is assessed (such as any one of the several sites that are edited in Blcap). The normality of the completely editing deficient animals unambiguously indicates that any difference in editing rates as defined from RNA-seq (in any form) ultimately is not physiologically relevant under standard housing conditions. It is also important to differentiate between drawing conclusions for an individual transcript as a whole rather than considering the individual sites themselves.*

*We believe that we have the most complete genetically controlled dataset that has been developed to discriminate these sites. The analysis of human GTEx data (Tan et al., Nature 2017) is limited in its ability to discriminate ADAR1 from ADAR2 specificity as there are no human samples with the different mutants. The murine data in this publication have single ADAR1 editing deficient mutants and single ADAR2 knock-out animals at a young age (3-4 weeks of age) but no compound mutants. The recent work from Licht et al., (Genome Res. 2019 Sep;29(9):1453-1463. doi: 10.1101/gr.242636.118) reports analysis of 14 day old mutants but lacks ADAR1 single mutants. We have assessed healthy 12 week old, sex, littermate matched whole brains. Our definition of sites is based on a high threshold – 50 reads or more per genotype and >0.01 editing (1% or more editing).*

*Differences in ascribed editing frequency arise from the computational analysis used to define editing frequency and the experimental method used to generate the underlying datasets (poly-A RNA-seq vs ribosome depleted RNA-seq vs mmPCR-seq vs Nascent-seq; the age and sex of the animals; the depth of dataset which has a very significant influence on the ability to identify sites; and the specific tissue/cells assessed). mmPCR-seq as applied by Tan et al., on the murine samples is significantly more sensitivity that standard RNA-seq due to the extremely high coverage per site that can be achieved. The conclusions from the recent publication by Licht et al., (Genome Res. 2019 Sep;29(9):1453-1463. doi: 10.1101/gr.242636.118) are independently supportive of the main conclusions we have reached.*

*Regarding the specific example provided by the reviewer – Blcap – we find 5 editing sites in total whereas the Licht et al., dataset reported 10. The three conserved sites provide an illustrative example of the characteristics of each dataset and the strength of conclusion that can be drawn from them. For the conserved sites: chr2:157558140 and chr2:157558149 are defined in our dataset as shared ADAR1/ADAR2 targets while chr2:157558110 is an ADAR1 specific editing site. From the Licht et al., dataset chr2:157558110 is an ADAR1 specific site (in agreement with our designation) whereas chr2:157558140 and chr2:157558149 are defined as ADAR1 specific as well in the Licht et al., dataset. This is called based on the presence of editing in the ADAR2 KO. However, the Licht et al., dataset lacks an ADAR1 mutant control (it has a WT, ADAR2 and double KO) and therefore could not detect the shared ADAR1/ADAR2 sites defined in our dataset. Read coverage also differs significantly between genotypes and across the respective datasets and this significantly impacts the confidence of called editing sites.*

Line 278: "Using definitive genetic controls, we conclude that Adar1 and Adar2 have similar editing site distributions" - The data show clearly that there are set of conserved site that edited only by ADAR2- how this fit to the said conclusion?

*Author Reply: We thank the reviewer for this comment. There are clearly examples of ADAR1 specific, ADAR2 specific and sites that can be edited by both enzymes– as presented in Figure 5B ~18% of sites in the whole brain are ADAR1 specific, ~25% are ADAR2 specific and the remainder (~47%) can be edited by either ADAR1 or ADAR2 interchangeably. The conserved sites represent a minor fraction of all possible editing in the mouse (~100 sites from >50-100,000 possible sites). As stated above, for any specific editing site/substrate in isolation there can be evidence for ADAR1 vs ADAR2 alone editing; however our conclusion is drawn from the analysis of the dataset as a whole (Figure 5c) and is supported by the recent independent analysis by Licht et al., (Genome Res. 2019 Sep;29(9):1453-1463. doi: 10.1101/gr.242636.118).*

The author can use their data to evaluate how accurate the RNA editing databases are, and how many of the deposited sites are indeed real editing events. This will be of high importance for the community, specifically about the coding sites.

*Author Reply: We agree that this will be a very useful outcome from the datasets we have generated.*

-The nomenclature is not consistent - the authors use both names of the enzyme, (adarb1 and adar2) throughout the manuscript. In order to avoid confusion- one name should be selected.

*Author Reply: We thank the reviewer for highlighting this and we have made all text and figures consistent. consistent. We have referred to the gene as Adarb1 (the official gene symbol) and when referencing the protein as ADAR2.*

Reviewer #2:

This work is an important contribution to our understanding of the role of editing in mammalian physiology. I would like very much to see it published, and believe it is going to be a well-cited reference for all the RNA editing community.

Therefore, I think it is of utmost importance to be very careful about the results and their interpretation. In addition, the data (which will be certainly further used by many) should be presented as fully as possible.

*Author Reply: We thank the reviewer for their positive endorsement of the work.*

Accordingly, I would like to make the following comments/requests (in no particular order):

1. A general comment: the authors show that ADAR-deficient animals seem to live and age normally under standard housing conditions. This shows that there are no essential editing sites (except for the GluRb Q/R one). However, this does not mean that "the majority of A-to-I editing is stochastic" (as stated in the conclusion), certainly not for recoding sites. It is very well possible that the phenotype of editing are subtle or required only under specific consequences. In fact, Horsch et al (Ref. 26) have identified some changes due to the absence of ADAR2, Jain et al (EMBO J 2018) have shown the effect of impaired FLNA editing, and so forth. Please discuss your results in the context of the above results, as well as the conservation of multiple recoding sites AND their ECS, and the enrichment of non-synonymous conserved recoding sites, both being a strong signal for positive selection.

*Author Reply: We present direct evidence that ADAR-deficient animals DO live and age normally, not that they “seem to”. The oldest animals at time of resubmission of the revision are 87 weeks of age. Our choice of the term “stochastic” was deliberate and is a conclusion from the analysis as a whole and consistent with the generally low levels of editing (<20%) for most sites in vivo. This has been demonstrated by multiple analysis methods and in many laboratories worldwide. As correctly noted by the reviewer there is strong evidence for specific and selective editing, particularly at a subset of recoding sites. It is worthwhile bearing in mind that these represent a minor fraction (<100 defined sites) of all possible editing in vivo. We agree with the evidence for positive selection and conservation of multiple recoding sites and the editing complementary sequences associated with these. However, our results unambiguously demonstrate that having no editing outside of GRIA2 is not only tolerated but results in viable, apparently normal animals (assuming no self-sensing by MDA5). Therefore, despite the strong conservation and selection of such sites the genetic evidence is that they do not have an essential physiological function under the conditions assessed.*

*We acknowledge the reviewer’s comments regarding the possibility of functions for recoding sites – we state this clearly in the original text on line 346-348 and lines 361-363. We included text (lines 361-364) clearly stating that our conclusion are drawn from standard housing conditions and that there may be subtle phenotypes or more profound phenotypes apparent under specific testing conditions. The recent studies of these genes in mice, such as FLNA referred to by the reviewer, generally utilises either 100% editing or 0% editing as this is what can be genetically engineered. This does not generally reflect the editing levels of these transcripts in vivo and so this caveat related to these studies has been included in our added comments. Our study has addressed this not from the perspective of a single editing site in a transcript, but by eliminating all but a single recoding site known to be essential. Therefore, we have not manipulated editing of a single site to non-physiological levels, but utilised a clean genetic system to test the entire editome in vivo. We have now added additional text in the discussion about the functional roles of editing in FLNA, NEIL1 and AZIN1.*

Similar statements are scattered throughout the paper (e.g. "the global absence of protein recoding does not have a deleterious physiological effect in vivo"). Please rephrase your conclusions and interpretations to avoid over-interpretation.

*Author Reply: We do not agree these statements are an over-interpretation but are an accurate reflection of the genetic result. The only potential caveat is that there is a single protein recoding event genetically engineered in the rescued mice (Gria2R/R) and the evidence is definitive that this is the only protein recoding event in the completely editing deficient animals.*

2. " Editing can occur at specific adenosines within a transcript, termed site selective editing, or at many sites within clusters, termed hyperediting or editing enriched regions" -- "clusters of sites" may mean different things. Most noncoding editing loci harbor clusters of sites, in the sense that there are multiple potentially-edited sites within a small region. However, the typical RNA molecule transcribed from these regions is edited in only one (or none) of these locations. The term hyper-editing usually refers to cases where a single molecule harbors multiple inosines. Anyway, please define clearly the terms you use.

*Author Reply: We have added the requested text. We detailed how we defined hyper-editing in lines 517-520 of the submitted manuscript (Methods section). This is based on the definition provided in the original publication from the Levanon lab (Porath et al., Nat Commun. 2014 Aug 27;5:4726. doi: 10.1038/ncomms5726). We have used 10 or more editing sites per 100bp to define hypereditied sites and this is now included in the text.*

3. Fig 1a-b shows the Mendelian ratios for ADAR1-het; ADAR2-null mice against themselves, or against ADAR1-null; ADAR2-null. The reader would like to see the full picture, including crossing ADAR1-het; ADAR2-het with themselves and all other combinations for which data is available. The methods should provide more information on the route through which the dKO mice were produced, beyond the general statement "we crossed the viable rescued Adar1-editing deficient animals (Adar1E861A/E861AIfih1-/-) (30, 38) with the Adar2-/-Gria2R/R animals (25)".

*Author Reply: We do not have breeding data outside of one or two pairs for intermediate crosses. Our animal ethics mandate that we use the most efficient strategies to generate mouse lines to reduce overall wastage in generating mutant mouse models. The heterozygous animals for Adar1E861A/+, Adarb1+/- and homozygous Ifih1-/- and Gria2R/R are all described to be normal in multiple independent data sets and with experience from many laboratories worldwide. The methods state how the line was generated – the Adar1-editing deficient animals (Adar1E861A/E861AIfih1-/-) were mated with the Adarb1-/-Gria2R/R animals – then the compound heterozygous progeny were inbred (Adar1E861A/+Ifih1+/-Adarb1+/-Gria2R/+; probability of generating dKO 1:64 (not considering the effect of the close genomic location of Adar1 and Gria2 so statistical frequency actually greater that this)). The major hurdle in these crosses is the close genomic locations of Adar (chr3:89,715,022-89,753,446) and Gria2 (UCSC mm10 chr3:80,681,450-80,802,835) on Chromosome 3 (~9MB apart), which required a miotic crossover event to get them on the same arm of the same chromosome. We therefore had to breed more than 300 pups to get the required mice. Preference was given to establishing Adar1E861A/+Ifih1-/-Adarb1+/-Gria2R/R (dKO frequency 1:8) animals for further inbreeding.*

4. Fig 1c - data for control mice (Ifih-/-, but having both ADARs functional) is missing. The triangular showing p-values should be rearranged to make the clustering into two groups more obvious. The same ordering should then be used in the legend above (to my color-vision challenged eyes, it is not so easy to connect the colors to the descriptions).

*Author Reply: We presented all data we have for completeness. The Ifih1-/- mice have been extensively described in many laboratories to be normal (PMID: 16714379) and in our previous paper we included analysis of these (Heraud-Farlow et al, Genome Biology 2017). We utilised a colour palette recommended for all colour-vision deficiencies (as described in Okabe and Ito 2008 - https://jfly.uni-koeln.de/color/ and on https://serialmentor.com/dataviz/) for the figures. We have included a supplemental file (Figure S2) with the genotypes labelled directly on the same dataset.*

5. Figure 1E - What is the p-value? (it might be low but NS, suggesting a bigger sample size could show a difference). Again, add data for Ifih-/-ADAR+/+ADAR2+/+ (if available), or at least for WT mice.

*Author Reply: P=0.25; from 31 control and 68 dKO (in Panel 1c which we now appreciate was difficult for the reviewer due to the labelling/colour choices). WT mice are not a good comparator as the effects of the Ifih1-/-Gria2R/R genotype need to be controlled for. We have utilised the Adar1E861A/+Ifih1-/-Adarb1+/-Gria2R/R (compound het) as the comparator as we demonstrate that it has normal editing (Figure 4 and 5) and histology.*

6. Figure 3 - again, various interesting comparisons are missing. Can you show the diff. expression of dKO against E861A (indirectly shown in 1E) ? This could highlight the effect of ADAR2 editing over the background of immune system changes. Other combinations could be interesting too. Four of the ten comparisons (each of the 5 genotypes against all others) appear in Figure 3, please calculate the others and then choose which one should appear in main text and the put the others as supp. figure.

*Author Reply: We have utilised two different datasets for these analyses. We used our previously published dataset (Heraud-Farlow et al., Genome Biology 2017) for the Adar1+/+Ifih1-/- and Adar1E861A/E861AIfih1-/- dataset (75bp paired end data) and the newly generated dataset from the Adar1E861A/+Ifih1-/-Adarb1+/-Gria2R/R (dHet), Adar1+/+Ifih1-/-Adarb1-/-Gria2R/R (Adar2-/-) and Adar1E861A/E861AIfih1-/-Adarb1-/-Gria2R/R (dKO) (150bp paired end). We attempted to compare all datasets but there is a significant batch effect between the two datasets which confounds the integration for direct comparisons. We have assessed the data against the controls from the same sequencing batches. The effect of ADAR2 deficiency in isolation can be assessed against the dHet. All data were provided in Supplemental Dataset 2 with all comparisons possible provided.*

7. The above comparisons do show a small number of genes that change substantially (in each case). Please elaborate.

*Author Reply: The few genes that are different between the Adar2-/- and dHet are largely non-coding RNAs of no known function. The vast majority of genes differentially expressed in any of the Adar1-editing deficient comparisons are related to the innate immune response. All data were provided in Supplemental Dataset 2 with all comparisons possible provided.*

8. Figure 4b: a few dots show (apparent) editing for the dKO. Please identify the sites and explain.

*Author Reply: We have assessed these when we obtained these results. They are likely SNPs specific to our strain of mice, due to sequence context of the adenosine which means there is likely errors from either sequencing or alignment, or are from batch effects of the sequencing. The list of sites and examples are now provided in Supp Figure 3.*

9. Please explain (in Methods or elsewhere) how you decide a site to be selective or hyper-edited (see also point #2 above)

*Author Reply: We have added the requested text. We detailed how we defined hyper-editing in lines 517-520 of the submitted manuscript (Methods section). This is based on the definition provided in the original publication from the Levanon lab (Porath et al., Nat Commun. 2014 Aug 27;5:4726. doi: 10.1038/ncomms5726). We have used 10 or more editing sites per 100bp to define hypereditied sites and this is now included in the text. Anything not within this criteria is defined as site selective.*

10. "Despite the single mutant phenotypes, there was no clear trend toward editing preference for repetitive elements for sites edited by Adar1 nor toward site-selective/recoding for Adar2 (Fig 5b)" However, Figure 5b and 5c do not provide data on repetitive elements. Please add numbers for B1 and B2 regions, known to harbor much of the mouse editing sites.

*Author Reply: We have added the editing at B1 and B2 elements as requested.*

11. "This same activity is now being exploited for targeted reprogramming of transcripts for potential therapeutic benefit (24, 42)." This sentence seems out of place. I don't see how it connects to what follows.

*Author Reply: We have removed this text.*

12. " Although one third of editing sites in brain were shared by Adar1 and Adar2, including many that are evolutionarily conserved and within coding regions, mice completely lacking A-to-I editing developed and aged normally. This demonstrates that Adar1 and Adar2 do not physiologically compensate in vivo." I think I understand what these sentences try to say: one could have thought that there are some essential sites among the many sites edited by both ADARs, but we now see there aren't. However, the sentences are not clear, please rephrase. In particular, what you show is not that "Adar1 and Adar2 do not physiologically compensate" (actually they do, in the sense of editing a list of shared substrates), but rather that there is no essential need in compensation. So either the shared substrates have no function at all, and then there is no need in compensation, or they do have a function that is not essential under normal housing, and then they do compensate.

*Author Reply: We thank the reviewer for seeking clarification of this text and we have rephrased.*

13. "Physiologically essential A-to-I editing comprises a small subset of the editome and that the majority of editing is dispensable for mammalian homeostasis. Moreover, in vivo biologically essential protein recoding mediated by A-to-I editing is an exception in mammals." -- if by "a small subset" you refer to the GluRB Q/R site only, I think you should be more specific. In addition, some caution should be taken when extrapolating from mouse to all mammals.

*Author Reply: We intended “small subset” to capture the editing of the essential substrates – for ADAR2 this is GRIA2 whilst for ADAR1 it is currently the prevailing view that a small subset of ADAR1 specific editing events are required to prevent MDA5 mediated innate immune response. The nature of the essential ADAR1 substrates is still unclear and an area of intense interest. We have extended this text to hopefully make this clear.*

**Second round of review**

**Reviewer 2**

The authors have answered satisfactorily many of my questions and comments (or explained why the additional data requested is not available). However, there are some issues that, in my opinion, still deserve some attention.

My main criticism is that (despite changes made in the present version) the interpretation of the results is still too strong at times, in my opinion. The experimental data shows that (accounting for the GRIA2 Q/R site and the immune response) complete lack of editing is tolerated with no detectable effect on lifespan or even expression profile. These are truly remarkable results. However, these results do not suggest that editing (in particular, recoding) is not physiological advantageous. Clearly, there are many ways in which a regulation mechanism can benefit an organism even if its absence does not lead to a shortened lifespan in the lab (not to mention the possibility that the slight decrease in lifespan which is actually seen in the present study is not just a random fluctuation but a true effect but statistically insignificant within the available sample size). In fact, this obvious observation was actually demonstrated by Horsch et al in the very same context, as I mentioned in my previous report.

In most places, the authors are careful to make this distinction, and talk about *essential* recoding events, etc. However, there are still some statements in the paper that may lead the reader to ignore the above distinction. For example, the manuscript reads (l. 255-258) "Evolutionarily conserved editing events, except the genomically engineered Gria2R/R, were absent in the Adar1E861A/E861AAdarb1-/- samples indicating that the global absence of protein recoding does not have a deleterious physiological effect in vivo (Fig 5a)". The usual meaning of "deleterious" in the context of genetics/evolution does include subtle effects that the present experiment is not sensitive too and cannot rule out. Clearly, individuals (as well as whole populations) may carry deleterious alleles but still breed and age normally, with zero or very small effect on their lifespan. Given the strong conservation seen for dozens of recoding sites (some of which are conserved across metazoa, see Porath et al 2019), and in some cases their ECSs, it is highly unlikely that deletion of their editing is non-deleterious, even if one takes into account the present results showing it does not shorten lifespan in lab.

For another example, lines 402-405 read "One consequence of the apparent normality of the Adar1E861A/E861AAdarb1-/- animals is that therapeutic targeting of ADAR1 activity, as proposed in cancer, is unlikely to cause unexpected on-target consequences outside of innate immune activation (20-22)". While I agree that a reasonable conclusion from the study is that therapeutic inactivation is not going to kill the patient (setting aside the question of extrapolating from mouse to human, which the authors have addressed), the conclusion that it is unlikely to cause any consequences whatsoever does not follow from the data, in my opinion.

Finally, the concluding sentence of the paper concludes that "An implication of these findings is that a significant proportion of A-to-I editing may be stochastic and its global absence does not result in profound phenotypic consequence to a whole organism in vivo" (a similar text also appears in l. 413-416). I agree that the majority of sites may be edited stochastically. This is a well accepted view, which is largely based on the fact that these sites are very weakly edited (few percent or less) and the assumption that such low level of editing is unlikely to have a functional role. Although this is a widely-held view, the possibility still remains that the weak sites do have some phenotypic effect. If this is the case, the effect is most likely very mild. Accordingly, the present study does not advance much our confidence in the above worldview. The fact that most editing sites are weak and the likely interpretation are long known. The present results do rule out the (extremely unlikely) possibility of weak sites having a major effect on lifespan, but leave open the possibility (less unlikely, thus leading alternative hypothesis) that the weak sites have a weak phenotypic effect, as already demonstrated in some cases. The importance of the present results is mainly in showing that lack of editing at the strongly edited sites, including many conserved recoding ones, stills allows for apparently normal life. This does not mean that these sites are stochastically edited.

Let me reiterate that I find the results presented in this manuscript truly thought-provoking, and I believe they should have a major impact on the way we should think about editing. That being said, I think it is very important that they are interpreted conservatively.

Minor issues:

The authors have clarified that by clusters of sites they refer to hyper-edited reads, in the sense defined by Porath et al 2014. The manuscript now reads (l. 86-88) "Editing can occur at

specific adenosines within a transcript, termed site selective editing, or at many sites

within an extended region, termed hyper-editing or editing enriched regions (3, 10-12)." It is worth mentioning that both of the above categories (selective editing, e.g. recoding, and hyper-editing) combined account for a minute fraction of the global editing activity. In fact, the vast majority of mRNA editing occurs within repetitive elements (e.g. Alu elements) that are weakly edited, so that each RNA molecule is edited unselectively, but in a few sites at most (not hyper-edited).

Official gene names are now used, so ADAR3 should be changed to Adarb2 for consistency.

The use of "preponderance" in l. 415 seems a bit strange.

I thank the authors for clarifying that by critical/essential sites they include the ones critical to avoids MDA5 response. However, that being the case I do not understand how they can deduce from the data that the essential sites are a small subset? After all, as the authors say, the number and identity of these immunogenic sites is currently unknown. Does that also follows from the distribution of editing levels?

**Authors Response**

**Point-by-point responses to the reviewers’ comments:**

Reviewer #2: The authors have answered satisfactorily many of my questions and comments (or explained why the additional data requested is not available). However, there are some issues that, in my opinion, still deserve some attention.

My main criticism is that (despite changes made in the present version) the interpretation of the results is still too strong at times, in my opinion. The experimental data shows that (accounting for the GRIA2 Q/R site and the immune response) complete lack of editing is tolerated with no detectable effect on lifespan or even expression profile. These are truly remarkable results. However, these results do not suggest that editing (in particular, recoding) is not physiological advantageous. Clearly, there are many ways in which a regulation mechanism can benefit an organism even if its absence does not lead to a shortened lifespan in the lab (not to mention the possibility that the slight decrease in lifespan which is actually seen in the present study is not just a random fluctuation but a true effect but statistically insignificant within the available sample size). In fact, this obvious observation was actually demonstrated by Horsch et al in the very same context, as I mentioned in my previous report.

*Response: We agree with the reviewer and believe that editing may be physiologically advantageous under certain circumstances and have stated this several times throughout the manuscript. We have made additional changes, however, to further emphasise this.*

*Although we have a large cohort of mice we acknowledge that subtle differences in survival may be missed and have added additional text to reflect this.*

In most places, the authors are careful to make this distinction, and talk about *essential* recoding events, etc. However, there are still some statements in the paper that may lead the reader to ignore the above distinction. For example, the manuscript reads (l. 255-258) "Evolutionarily conserved editing events, except the genomically engineered Gria2R/R, were absent in the Adar1E861A/E861AAdarb1-/- samples indicating that the global absence of protein recoding does not have a deleterious physiological effect in vivo (Fig 5a)". The usual meaning of "deleterious" in the context of genetics/evolution does include subtle effects that the present experiment is not sensitive too and cannot rule out. Clearly, individuals (as well as whole populations) may carry deleterious alleles but still breed and age normally, with zero or very small effect on their lifespan. Given the strong conservation seen for dozens of recoding sites (some of which are conserved across metazoa, see

Porath et al 2019), and in some cases their ECSs, it is highly unlikely that deletion of their editing is non-deleterious, even if one takes into account the present results showing it does not shorten lifespan in lab.

*Response: We thank the reviewer for the detailed assessment of our work and appreciate their position on interpretation of the results. We have now amended the sentence, changing “deleterious” to “pathogenic” and to include the caveat that this is under standard housing conditions:*

*"Evolutionarily conserved editing events, except the genomically engineered Gria2R/R, were absent in the Adar1E861A/E861AAdarb1-/- samples indicating that the global absence of protein recoding does not have a pathogenic effect in vivo, under standard housing conditions (Fig 5a)".*

For another example, lines 402-405 read "One consequence of the apparent normality of the Adar1E861A/E861AAdarb1-/- animals is that therapeutic targeting of ADAR1 activity, as proposed in cancer, is unlikely to cause unexpected on-target consequences outside of innate immune activation (20-22)". While I agree that a reasonable conclusion from the study is that therapeutic inactivation is not going to kill the patient (setting aside the question of extrapolating from mouse to human, which the authors have addressed), the conclusion that it is unlikely to cause any consequences whatsoever does not follow from the data, in my opinion.

*Response: We have deleted this sentence.*

Finally, the concluding sentence of the paper concludes that "An implication of these findings is that a significant proportion of A-to-I editing may be stochastic and its global absence does not result in profound phenotypic consequence to a whole organism in vivo" (a similar text also appears in l. 413-416). I agree that the majority of sites may be edited stochastically. This is a well accepted view, which is largely based on the fact that these sites are very weakly edited (few percent or less) and the assumption that such low level of editing is unlikely to have a functional role. Although this is a widely-held view, the possibility still remains that the weak sites do have some phenotypic effect. If this is the case, the effect is most likely very mild. Accordingly, the present study does not advance much our confidence in the above worldview. The fact that most editing sites are weak and the likely interpretation are long known. The present results do rule out the (extremely unlikely) possibility of weak sites having a major effect on lifespan, but leave open the possibility (less unlikely, thus leading alternative hypothesis) that the weak sites have a weak phenotypic effect, as already demonstrated in some cases. The importance of the present results is mainly in showing that lack of editing at the strongly edited sites, including many conserved recoding ones, stills allows for apparently normal life. This does not mean that these sites are stochastically edited.

*Response: We thank the reviewer for these comments and agree with this summation of previous assumptions within the field and the importance of this result. We have added a more nuanced discussion of these possibilities in the text and clarified that we are not suggesting that strongly edited conserved sites are stochastically edited. However, given that these are a small proportion of total editing, we do not think it is incorrect to state that the majority of sites are weakly edited in a stochastic manner and seemingly don’t have a profound impact. Even if that was the previous assumption based purely on editing level, it is important to definitively show that. We have again reiterated that our results do not rule out the possibility of more subtle phenotypic effects of these editing sites.*

Let me reiterate that I find the results presented in this manuscript truly thought-provoking, and I believe they should have a major impact on the way we should think about editing. That being said, I think it is very important that they are interpreted conservatively.

*Response: We thank the reviewer for the positive assessment of our work and their commitment to ensuring the manuscript represents a conservative interpretation of the results.*

Minor issues:

The authors have clarified that by clusters of sites they refer to hyper-edited reads, in the sense defined by Porath et al 2014. The manuscript now reads (l. 86-88) "Editing can occur at

specific adenosines within a transcript, termed site selective editing, or at many sites

within an extended region, termed hyper-editing or editing enriched regions (3, 10-12)." It is worth mentioning that both of the above categories (selective editing, e.g. recoding, and hyper-editing) combined account for a minute fraction of the global editing activity. In fact, the vast majority of mRNA editing occurs within repetitive elements (e.g. Alu elements) that are weakly edited, so that each RNA molecule is edited unselectively, but in a few sites at most (not hyper-edited).

*Response: We have added the following sentence “The vast majority of editing is weak and occurs within repetitive elements (eg Alu elements in humans, SINEs in mice).”*

Official gene names are now used, so ADAR3 should be changed to Adarb2 for consistency.

*Response: We have changed the text accordingly*

The use of "preponderance" in l. 415 seems a bit strange.

*Response: We have changed the text to the more commonly used synonym “majority”*

*I thank the authors for clarifying that by critical/essential sites they include the ones critical to avoids MDA5 response. However, that being the case I do not understand how they can deduce from the data that the essential sites are a small subset? After all, as the authors say, the number and identity of these immunogenic sites is currently unknown. Does that also follows from the distribution of editing levels?*

*Response: The reviewer is correct that from our data we cannot be sure how many of the ADAR1 editing sites are necessary to suppress MDA5 activation on an as yet undefined set of transcripts. However, the general consensus is that they are represented within the hyperedited regions. As the reviewer stated in the introduction, the vast majority of sites are weakly edited non-selectively in repetitive elements, which are therefore very unlikely to be the immunogenic RNAs that activate MDA5 given the known biochemistry of MDA5 substrate preference and structure.*
